# Sensitivity comparison of longitudinal cognitive function indicators of Alzheimer’s disease after mild cognitive impairment: a prospective cohort study

**DOI:** 10.1038/s41598-026-44192-2

**Published:** 2026-03-22

**Authors:** Guiya Guo, Wangchen Song, Aimin Wang, Qingxia Cui, Xinyu Yang, Yanxia Wang, Yonghua Ma, Hairui Han, Zihui Li, Zhaoxue Zhang, Weijing Meng, Suzhen Wang, Fuyan Shi

**Affiliations:** 1 Department of Health Statistics, School of Public Health, Shandong Second Medical University, Weifang, Shandong China; 2 Department of Mathematical Statistics, School of Public Health, Shandong Second Medical University, Weifang, Shandong China; 3 Experimental Teaching Center for Public Health and Management, School of Public Health, Shandong Second Medical University, Weifang, Shandong China

**Keywords:** 3 Alzheimer’s disease, 4 CDR-SB, 5 FAQ, 6 Mild cognitive impairment, 7 Risk prediction, 8 Semi-parametric joint model, Biomarkers, Diseases, Medical research, Neurology, Neuroscience

## Abstract

Accurate prediction of progression from mild cognitive impairment (MCI) to Alzheimer’s disease (AD) is critical for early intervention. Many existing models lack the ability to capture the nonlinear nature of cognitive decline and individual heterogeneity. This study employed a semi‑parametric joint model to analyze longitudinal cognitive trajectories and identify robust predictors of conversion. Data from 596 participants (184 AD converters, 412 stable MCI) were obtained from the Alzheimer’s Disease Neuroimaging Initiative (ADNI). Longitudinal assessments included ADAS‑Cog13, ADAS‑Cog11, CDR‑SB, FAQ, RAVLT‑IR, RAVLT‑L, and MMSE. A semi‑parametric joint model combining B‑splines for the longitudinal process with a Cox survival submodel was fitted for each cognitive measure. Model performance was evaluated using AIC, BIC, intraclass correlation coefficient (ICC), time‑dependent C‑index, dynamic AUC, and calibration curves. Subgroup analyses were conducted by APOE‑ε4 carrier status. In multivariable joint models, APOE‑ε4 carriage was a consistent risk factor (HR range: 1.38–1.77). Higher scores on ADAS‑Cog13 (HR = 3.71 per SD), ADAS‑Cog11 (HR = 2.71), CDR‑SB (HR = 3.79), and FAQ (HR = 2.85) increased the hazard of conversion, whereas higher scores on RAVLT‑IR (HR = 0.23), RAVLT‑L (HR = 0.14), and MMSE (HR = 0.53) were protective. All models showed high ICCs (0.94–0.98) and moderate‑to‑good predictive accuracy over 2, 5, and 8 year horizons (C‑index: 0.585–0.668). CDR‑SB and FAQ exhibited the strongest effect sizes and clearest dose‑dependent trajectories across APOE‑ε4 subgroups. Calibration curves demonstrated good agreement between predicted and observed survival. The semi‑parametric joint model effectively captures nonlinear cognitive‑functional decline and provides validated predictions of AD risk. APOE‑ε4 genotype combined with longitudinal monitoring of CDR‑SB and FAQ offers a robust framework for stratifying progression risk in clinical MCI management.

**Supplementary Information:**

The online version contains supplementary material available at 10.1038/s41598-026-44192-2.

## Introduction

Alzheimer’s disease (AD) is the most common neurodegenerative disorder and poses a substantial public health burden worldwide^1^. In China alone, more than 15 million individuals are projected to be affected by 2025, with prevalence notably higher in rural than urban regions^2^. Pathologically, AD is defined by amyloid‑β(Aβ) deposition and hyperphosphorylated tau accumulation, yet its clinical manifestation—especially during the prodromal mild cognitive impairment (MCI) stage—is highly heterogeneous^3,4^. While 10%–15% of MCI patients progress to AD annually, individual risk varies widely, underscoring the need for accurate, personalized prediction tools to guide early intervention and clinical management^5^.

Current approaches to predicting MCI‑to‑AD conversion often integrate biomarkers (e.g., CSF Aβ_42_/Aβ_40_, plasma neurofilament light), neuroimaging measures (e.g., hippocampal volume), and cognitive assessments^6^. Among cognitive tools, the Alzheimer’s Disease Assessment Scale–Cognitive subscale (ADAS‑Cog), Clinical Dementia Rating Sum of Boxes (CDR‑SB), Functional Activities Questionnaire (FAQ), Rey Auditory Verbal Learning Test (RAVLT), and Mini‑Mental State Examination (MMSE) are widely used, each reflecting distinct cognitive or functional domains^7,8^. However, two critical methodological gaps remain. First, most predictive models treat cognitive scores as baseline or linearly changing variables, failing to capture their frequently nonlinear, phase‑specific trajectories over time^9^. Second, although genetic factors such as APOE‑ε4 carriage markedly modulate progression risk, few studies systematically compare how longitudinal cognitive trajectories differ across APOE‑ε4 genotype subgroups, limiting personalized risk stratification^10,11^.

To address these limitations, we employed a semi‑parametric joint modeling framework that combines B‑spline‑based longitudinal submodels with a Cox survival submodel. This approach allows flexible estimation of nonlinear temporal trends in each cognitive measure while directly linking these trajectories to the hazard of AD conversion^12,13^. Using updated data from the Alzheimer’s Disease Neuroimaging Initiative (ADNI), we aimed to (1) quantify the dynamic, nonlinear effects of seven key cognitive/functional measures on conversion risk^14,15^; (2) identify consistent risk factors, including APOE‑ε4 genotype and demographic variables ^16^; (3) compare longitudinal trajectories across APOE‑ε4 carrier subgroups to elucidate genotype‑dependent patterns of decline; and (4) comprehensively evaluate model fit, discriminative accuracy, and calibration to support the clinical utility of the most informative measures^17,18^.

## Materials and methods

### Data source

Data used in the preparation of this article were obtained from the Alzheimer’s Disease Neuroimaging Initiative (ADNI) database (adni.loni.usc.edu)^19^. ADNI is a longitudinal multicenter study designed to develop clinical, imaging, genetic, and biochemical biomarkers for the early detection and tracking of Alzheimer’s disease (AD)^20^ The present analysis primarily utilizes data from the ADNI 1, Grand Opportunity (GO), and ADNI 2 phases, covering clinical assessments conducted between May 2006 and October 2017. The study includes participants across the United States and Canada who are cognitively normal, have mild cognitive impairment (MCI), or have AD. Further details regarding protocols, diagnostic criteria, and assessments are available in the primary ADNI publications^21^.

### Study participants

Participants were included if they had a baseline diagnosis of MCI and at least three follow-up visits with complete longitudinal cognitive assessments. Exclusion criteria were: (1) fewer than three follow-up visits before AD diagnosis or by the last follow-up; (2) missing scores on key longitudinal cognitive scales. After screening, 596 participants were included in the final analytical sample^22^. Among them, 184 progressed to AD (case group) and 412 remained stable MCI (control group). Follow-up time was calculated from the date of the first measurement of longitudinal cognitive variable (ADAS-Cog13, ADAS-Cog11, CDR-SB, FAQ, RAVLT-IR, RAVLT-L, or MMSE) until AD diagnosis, loss to follow-up, or the end of the study period. For 43 participants whose last cognitive assessment occurred more than 6 months before AD diagnosis, the event was treated as censored at the last assessment date. The median interval between consecutive cognitive assessments was approximately 354 days (about 11.7 months).

### Variable definitions

Baseline covariates included sex (male/female), age, years of education, and APOE ε4 allele status (non carrier, heterozygous, homozygous). Longitudinal cognitive measures comprised:(1) ADAS-Cog13: A 13 item scale assessing memory, language, praxis, and orientation (range 0–85, higher scores indicate worse function).(2) ADAS-Cog11: An 11 item version excluding delayed recall and number cancellation items (range 0–70).(3) CDR SB: Sum of boxes from the Clinical Dementia Rating scale, evaluating six functional domains (range 0–18, higher scores indicate greater impairment).(4) FAQ: Functional Activities Questionnaire assessing instrumental activities of daily living (range 0–30, higher scores indicate worse function).(5) RAVLT IR: Rey Auditory Verbal Learning Test immediate recall (range 0–75, lower scores indicate poorer memory).(6) RAVLT L: RAVLT delayed recall (range 0–15, lower scores indicate poorer memory).(7) MMSE: Mini Mental State Examination, a global cognitive screening tool (range 0–30, lower scores indicate worse function).All longitudinal cognitive measures were standardized using Z score normalization before analysis to improve interpretability and model stability^23,24^.

### Statistical analysis

Descriptive statistics for baseline characteristics were calculated according to data distribution. The categorical variable, sex, is presented as frequencies and percentages, and group comparisons were performed using the chi‑square (χ^2^) test. Ordinal or continuous variables that were not normally distributed, including APOE‑ε4 allele status, age, years of education, and all cognitive measures (ADAS‑Cog13, ADAS‑Cog11, CDR‑SB, FAQ, RAVLT‑IR, RAVLT‑L, MMSE), are summarized as median and interquartile range (IQR). Between‑group differences for these variables were evaluated using the non‑parametric Mann‑Whitney U test. All statistical tests were two‑sided, with a significance level of *P* < 0.05.

### Joint model

*Longitudinal Submodel :*A nonparametric multiplicative random effects model was used to capture the trajectories of each cognitive measure over time:

$$  Y_{i} \left( t \right) = m_{i} \left( t \right) + \varepsilon_{i} \left( t \right) = b_{i} \times{\bf {B}}^{{ \top }} \left( t \right){\bf\gamma} + \varepsilon_{i} \left( t \right)$$

where ${Y}_{i}$ is the observed cognitive score for subject i at time t, $\mathbf{B}\left(t\right)$ is a vector of cubic B spline basis functions constructed using the bs() function in R, $\gamma$ is the corresponding coefficient vector, and ${b}_{i}$ is a subject specific random effect following $N\left(1,{\sigma }_{b}^{2}\right)$. The measurement error ${\varepsilon }_{i}\left(t\right)$ is assumed i.i.d. normal with mean zero and variance ${\sigma }_{e}^{2}$. The B spline was specified with boundary knots at 0 and 10.6 years, covering the entire follow up period. The number of internal knots was determined by the degrees of freedom (df). Based on AIC and BIC comparisons across df = 2–5, we selected df = 3 (equivalent to one internal knot) for all cognitive measures. The random effects structure included only a subject specific random intercept (random =  ~ 1 | PTID). This choice of a multiplicative random intercept, rather than an additive random slope, implies that individual trajectories share a common shape determined by the B‑spline basis but are scaled by a subject‑specific factor. While this does not directly model heterogeneity in the rate of decline, the flexibility of the B‑splines allows the population‑level curve to capture nonlinear trends, and the scalar random effect accounts for overall differences in level and progression among subjects. Model diagnostics (e.g., high intraclass correlation coefficients > 0.94) indicated that between‑subject heterogeneity is the dominant source of variation, supporting the adequacy of this parsimonious structure. In preliminary analyses, alternative specifications with random slopes were also considered; however, the NMRE model was ultimately selected due to its computational stability, compatibility with the JSM package, and satisfactory goodness‑of‑fit based on AIC/BIC comparisons.

*Survival Submodel:* The time to AD conversion was modeled using a Cox proportional hazards model with the entire longitudinal trajectory (excluding measurement error) included as a time dependent covariate:

$$ \lambda \left( {t{ \mid }b_{i} ,m_{i} \left( t \right),{\bf{W}}_{i} } \right) = \lambda_{0} \left( t \right)\exp \left\{ {{\bf{W}}_{i}^{{ \top }} {\bf{\phi}} + \alpha \;m_{i} \left( t \right)} \right\}$$

where ${{\boldsymbol{W}}}_{i}$ contains baseline covariates (sex, age, education, APOE ε4 status), *ϕ* is the vector of their coefficients, α quantifies the association between the longitudinal trajectory and the hazard of AD, and ${\lambda }_{0}(t)$ is the unspecified baseline hazard function.

*Estimation and Model Details:* The joint model was fitted using the jmodelMult() function in the JSM package, which implements an expectation maximization (EM) algorithm. The longitudinal and survival submodels were linked by assuming that the entire underlying trajectory ${m}_{i}\left(t\right)$ enters the survival model (i.e., model = 1). Convergence criteria were set as tolP = 1e-3 (parameter change) and tolL = 1e-6 (log likelihood change), with a maximum of 300 EM iterations. Standard errors were estimated via the profile likelihood forward difference method (SE.method = “PLFD”), and integrals over the random effects were approximated using Gauss–Hermite quadrature with 3 knots.

*Model Validation and Diagnostics:*We validated the proportional hazards assumption using Schoenfeld residual tests and examined residual versus time and residual versus fitted plots for the longitudinal submodel to check for systematic patterns. The normality of random effects was assessed using Shapiro–Wilk tests and Q-Q plots. Missing longitudinal scores were imputed using multiple imputation (m = 20) under the missing at random assumption, validated by logistic regression and sensitivity analysis.

### Subgroup analysis

Participants were stratified into three subgroups based on APOE ε4 allele status: non carriers (n = 318), heterozygous carriers (n = 216), and homozygous carriers (n = 62). For each cognitive measure, we fitted separate nonparametric multiplicative random effects models within each subgroup to compare cognitive trajectories. Cubic B spline curves with df = 3 and boundary knots at 0 and 10.6 years were used. The fitted trajectories were visualized along with pointwise 95% confidence bands, and each subgroup’s curve was annotated with the P values for the three B spline basis functions to indicate the significance of linear and nonlinear components.

### Model evaluation

*Model Comparison:* Each joint model (one for each cognitive indicator) was evaluated using:(1) Akaike Information Criterion (AIC) and Bayesian Information Criterion (BIC): lower values indicate better model parsimony. (2) Log likelihood (logLik): higher values reflect greater goodness of fit (3) Intraclass correlation coefficient (ICC): calculated as the proportion of total variance attributable to between subject heterogeneity.

*Predictive Accuracy Assessment:*To validate the joint models’ predictive performance, we computed: (1) Time dependent C index at 2, 5, and 8 years. (2) Dynamic area under the ROC curve (AUC) at the same time points. (3) Calibration curves comparing predicted vs. observed survival probabilities^25,26^.

### Quality control

All statistical analyses were performed using R version 4.3.0. Categorical variables were analyzed with χ^2^ tests. Continuous variables were compared using Mann Whitney U tests. A two tailed *P* value < 0.05 was considered statistically significant. Joint modeling was conducted with the JSM package (version 1.0.1), and survival plots were generated using the survminer package.

## Results

### Baseline characteristics and univariate analysis

**Fig. 1:**
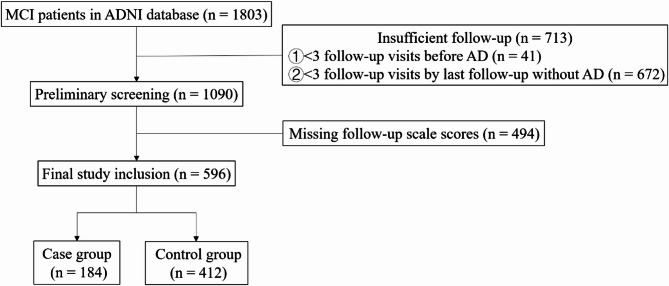
Screening Process of Research Subjects.

A total of 596 participants were included in this study, comprising 184 AD converters and 412 stable MCI individuals (Fig. 1). The median follow‑up time for the entire cohort was 2.50 years (IQR 1.60–2.90). The AD group had a median follow‑up of 2.40 years (IQR 1.48–2.60), while the MCI group was followed for a median of 2.65 years (IQR 2.50–3.60).

Baseline characteristics and univariate comparisons are presented in Table 1. Sex distribution did not differ significantly between the two groups (*P* = 0.649). APOE‑ε4 allele frequency was significantly higher in the AD group (*P* < 0.001): non‑carriers 36.95% (68 / 184) vs. 60.68% (250 / 412) in MCI; heterozygous 46.20% (85 / 184) vs. 31.80% (131 / 412); homozygous 16.85% (31 / 184) vs. 7.52% (31 / 412). The AD group was older than the MCI group [median (IQR): 74.50 (69.90–79.60) vs. 72.50 (66.50–78.30) years, *P* = 0.003], while years of education did not differ (*P* = 0.897).

**Table 1:** Baseline Characteristics of Study Subjects and Univariate Analysis.

Factor	AD (n = 227)	MCI (n = 369)	χ2/ W*	P
Sex^a^			0.21	0.649^c^
Female	72(39.13)	171(41.50)		
Male	112(60.87)	241(58.50)		
APOE-ε4 allele^a^			28,199.00	< 0.001 ^d^
Non	68(36.95)	250(60.68)		
Heterozygous	85(46.20)	131(31.80)		
Homozygous	31(16.85)	31(7.52)		
Age^b^	74.50(69.90–79.60)	72.50(66.50–78.30)	32,032.00	0.003^d^
Education years^b^	16.10(14.00–18.00)	16.10(14.00–18.00)	38,153.00	0.897^d^
ADAS-Cog13^b^	20.10(16.20–24.70)	13.20(8.67–17.30)	16,433.50	< 0.001^d^
ADAS-Cog11^b^	12.40(9.33–15.10)	8.39(5.33–11.00)	18,603.50	< 0.001^d^
CDR-SB^b^	2.01(1.38–2.50)	1.21(0.50–1.50)	20,676.50	< 0.001^d^
FAQ^b^	11.80(3.00–18.20)	4.86(0.00–7.00)	19,997.00	< 0.001^d^
RAVLT-IR^b^	28.40(24.00–33.00)	36.10(28.00–43.00)	54,235.50	< 0.001^d^
RAVLT-L^b^	1.60(0.00–2.00)	4.67(1.00–7.00)	57,163.50	< 0.001^d^
MMSE^b^	26.50(25.00–28.00)	28.10(27.00–29.00)	54,925.00	< 0.001^d^

* a: n(%); b: M(IQR) ; c: χ^2^ test​; d:Mann–Whitney U test ​.

Regarding cognitive measures, the AD group showed significantly higher scores on ADAS‑Cog13 [20.10 (16.20–24.70) vs. 13.20 (8.67–17.30), *P* < 0.001], ADAS‑Cog11 [12.40 (9.33–15.10) vs. 8.39 (5.33–11.00), *P* < 0.001], CDR‑SB [2.01 (1.38–2.50) vs. 1.21 (0.50–1.50), *P* < 0.001], and FAQ [11.80 (3.00–18.20) vs. 4.86 (0.00–7.00), *P* < 0.001]. Conversely, the AD group had lower scores on RAVLT‑IR [28.40 (24.00–33.00) vs. 36.10 (28.00–43.00), P < 0.001], RAVLT‑L [1.60 (0.00–2.00) vs. 4.67 (1.00–7.00), *P* < 0.001], and MMSE [26.50 (25.00–28.00) vs. 28.10 (27.00–29.00), *P* < 0.001].

In summary, APOE‑ε4 carriage (especially homozygous status), older age, and higher scores on ADAS‑Cog, CDR‑SB, and FAQ were risk factors for AD conversion, whereas higher scores on RAVLT‑IR, RAVLT‑L, and MMSE appeared protective.

### Multivariable analysis

Joint models incorporating seven longitudinal cognitive measures consistently demonstrated significant associations between cognitive trajectories and the risk of AD conversion. In the longitudinal submodels, several B‑spline basis functions (bs1, bs2, bs3) showed significant time‑dependent effects, indicating nonlinear progression over the follow‑up period. For example, the third basis function (bs3) was highly significant for ADAS‑Cog13 (β = 0.344, *P* < 0.001), CDR‑SB (β = 0.287, *P* = 0.005), and FAQ (β = 0.356, *P* < 0.001), confirming distinct nonlinear patterns in these measures.

In the survival submodels, each cognitive measure was a strong and independent predictor of AD risk after adjustment for sex, age, education, and APOE‑ε4 status. Higher scores on ADAS‑Cog13 (HR = 3.705 per SD increase, *P* < 0.001), ADAS‑Cog11 (HR = 2.707, *P* < 0.001), CDR‑SB (HR = 3.794, *P* < 0.001), and FAQ (HR = 2.853, *P* < 0.001) were associated with increased hazard of conversion. Conversely, higher scores on RAVLT‑IR (HR = 0.234, *P* < 0.001), RAVLT‑L (HR = 0.143, *P* < 0.001), and MMSE (HR = 0.531, *P* < 0.001) were protective. APOE‑ε4 carriage was a consistent risk factor across all models (all *P* < 0.01), with hazard ratios ranging from 1.377 (ADAS‑Cog13) to 1.773 (MMSE). Female sex was associated with higher risk in models including FAQ (HR = 1.553, *P* = 0.006) and RAVLT‑IR (HR = 1.441, *P* = 0.005). Age and education showed variable significance depending on the cognitive measure included (Table 2).

**Table 2:** Joint Model Analysis for Alzheimer’s Disease Risk Prediction Based on Seven Longitudinal Cognitive Assessment Measures.

Factor	ADAS-Cog13			ADAS-Cog11			CDR-SB			FAQ		
	β/HR	Z	P	β/HR	Z	P	β/HR	Z	P	β/HR	Z	P
*Longitudinal Submodel*												
bs_1_	0.032	0.032	0.310	0.233	0.050	< 0.001	0.242	0.048	< 0.001	0.096	0.032	0.003
bs_2_	0.037	0.067	0.581	0.018	0.106	0.865	0.188	0.093	0.044	0.054	0.061	0.383
bs_3_	0.344	0.073	< 0.001	-0.052	0.152	0.731	0.287	0.102	0.005	0.356	0.080	< 0.001
*Survival Submodel*												
Female	0.970	(0.738–1.276)	0.829	0.925	(0.715–1.197)	0.555	1.182	(0.923–1.514)	0.185	1.553	(1.134–2.124)	0.006
Age	1.012	(0.989–1.036)	0.309	1.022	(0.999–1.046)	0.057	1.021	(0.997–1.046)	0.084	1.034	(1.011–1.057)	0.004
Education (yrs)	1.063	(1.008–1.120)	0.024	1.032	(0.979–1.087)	0.240	1.049	(0.992–1.109)	0.095	1.054	(0.997–1.114)	0.063
APOE-ε4	1.377	(1.100–1.723)	0.005	1.538	(1.244–1.901)	< 0.001	1.605	(1.293–1.992)	< 0.001	1.748	(1.387–2.202)	< 0.001
Cognitive	3.705	(2.973–4.617)	< 0.001	2.707	(2.273–3.224)	< 0.001	3.794	(3.083–4.669)	< 0.001	2.853	(2.379–3.421)	< 0.001

Factor	RAVLT-IR			RAVLT-L			MMSE		
	β/HR	Z	P	β/HR	Z	P	β/HR	Z	P
*Longitudinal Submodel*									
bs1	0.038	0.030	0.203	0.019	0.027	0.498	-0.167	0.049	0.001
bs2	-0.065	0.052	0.213	-0.072	0.058	0.217	-0.136	0.093	0.145
bs3	-0.099	0.057	0.084	0.240	0.084	0.004	-0.127	0.109	0.245
*Survival Submodel*									
Female	1.441	(1.116–1.860)	0.005	1.247	(0.927–1.677)	0.143	0.940	(0.721–1.225)	0.646
Age	1.021	(0.999–1.044)	0.060	1.023	(1.000–1.047)	0.046	1.047	(1.025–1.071)	< 0.001
Education (yrs)	1.096	(1.038–1.157)	0.001	1.061	(1.003–1.122)	0.038	1.045	(0.992–1.101)	0.098
APOE-ε4	1.724	(1.427–2.083)	< 0.001	1.459	(1.191–1.789)	< 0.001	1.773	(1.447–2.172)	< 0.001
Cognitive	0.234	(0.184–0.297)	< 0.001	0.143	(0.102–0.200)	< 0.001	0.531	(0.474–0.597)	< 0.001

*Hazard ratios (HRs) represent the change in risk per 1‑standard‑deviation (SD) increase in each cognitive measure. The mean (SD) of raw scores were: ADAS‑Cog13, 15.54 (8.02); ADAS‑Cog11, 9.76 (5.37); CDR‑SB, 1.66 (1.36); FAQ, 8.49 (9.91); RAVLT‑IR, 34.92 (11.94); RAVLT‑L, 3.96 (4.20); MMSE, 27.38 (2.52).

### Subgroup analysis

**Fig. 2:**
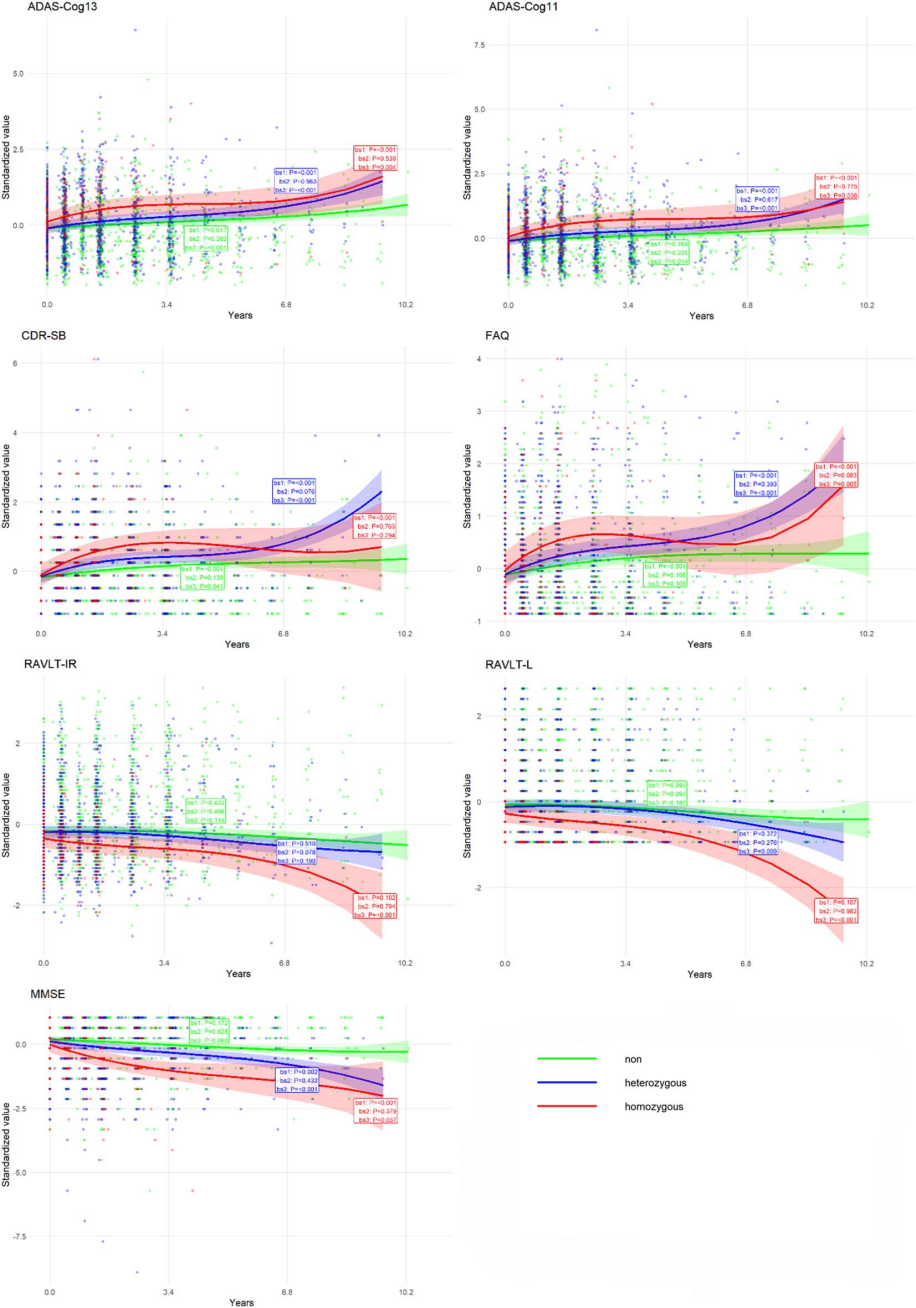
APOE-ε4 Stratified Cognitive Trajectories via Cubic B-Splines.

Based on APOE‑ε4 carrier status (non‑carriers, heterozygous, homozygous), we examined longitudinal trajectories of cognitive measures across subgroups. Each trajectory is annotated with P‑values for the three B‑spline basis functions (bs₁, bs₂, bs₃), which reflect the significance of both linear and nonlinear time components (Fig. 2).

A clear dose‑response relationship was observed: a higher APOE‑ε4 allele count was associated with earlier AD conversion. Among all cognitive measures, CDR‑SB and FAQ showed the most pronounced inter‑group divergence in longitudinal slopes, with homozygous carriers exhibiting the steepest functional decline over time. For other measures, trajectory patterns varied by subgroup, and the annotated P‑values help distinguish periods of significant linear or nonlinear change. Together, these visualizations highlight how genetic risk modulates the pace and pattern of cognitive deterioration in MCI patients.

### Model evaluation

Model performance metrics for the seven joint models are summarized in Table 3. All models exhibited high intraclass correlation coefficients (ICC range: 0.94–0.98), indicating that the majority of variance in longitudinal cognitive scores was attributable to stable between‑subject differences. Akaike and Bayesian information criteria (AIC, BIC) and log‑likelihood values were comparable across models, suggesting similar overall fit and parsimony.

**Table 3:** Evaluation of Joint Models Based on Seven Longitudinal Cognitive Assessment Measures.

Model	ICC	AIC	BIC	logLik
ADAS-Cog13	0.97	8439.20	8505.05	-4204.60
ADAS-Cog11	0.95	8895.38	8961.23	-4432.69
CDR-SB	0.95	9108.94	9174.79	-4539.47
FAQ	0.98	9053.54	9119.40	-4511.77
RAVLT-IR	0.97	7911.46	7977.32	-3940.73
RAVLT-L	0.98	8326.28	8392.13	-4148.14
MMSE	0.94	9211.02	9276.87	-4590.51

**Fig. 3:**
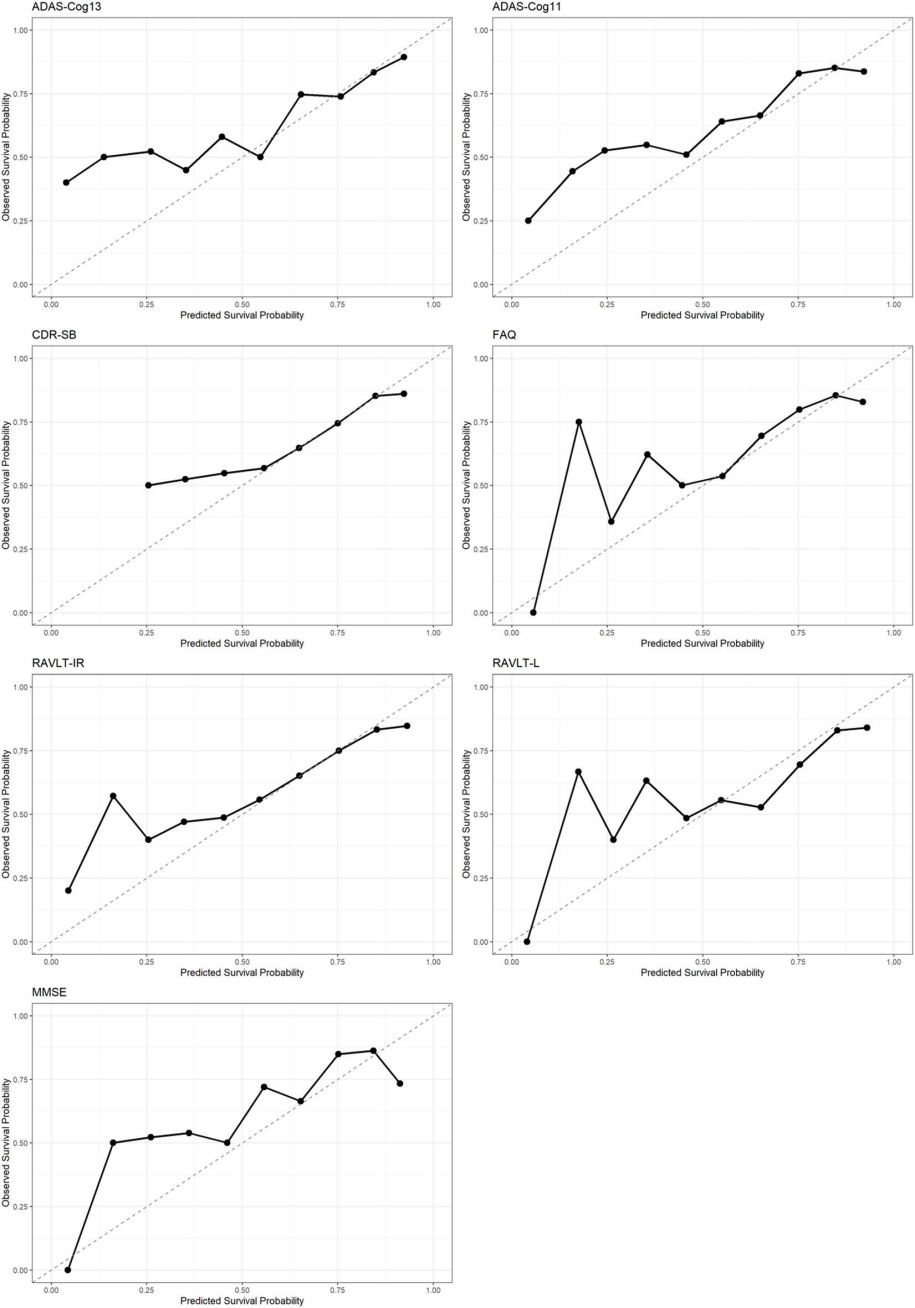
Calibration of Seven Joint Models at 5 Years.

**Fig. 4:**
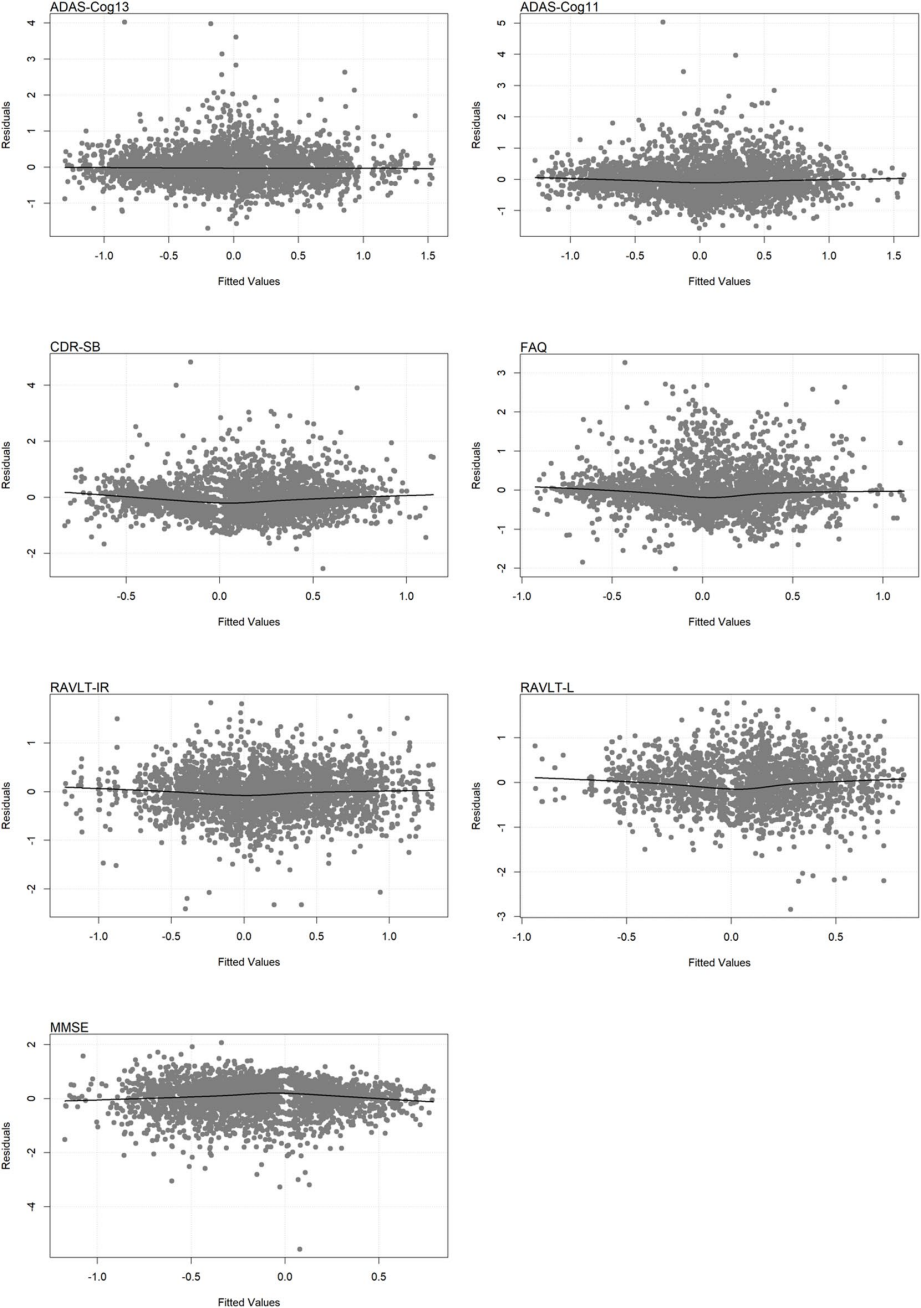
Residuals vs. Fitted Values for the Seven Joint Models.

**Fig. 5:**
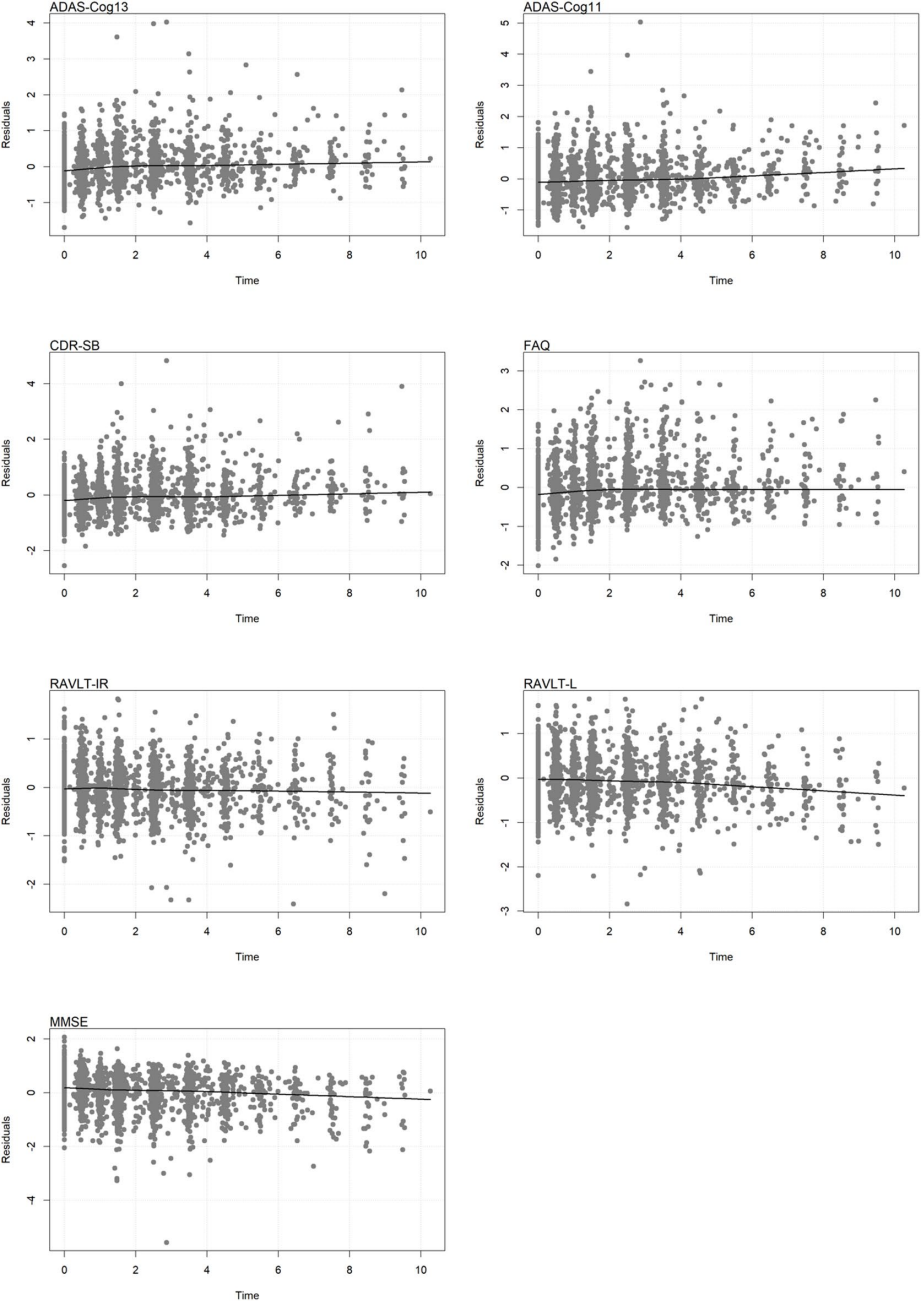
Residuals vs. time for the Seven Joint Models.

Predictive accuracy was assessed using time‑dependent C‑indices and dynamic AUCs at 2, 5, and 8 years (Table 4). All models demonstrated moderate to good discriminative ability, with C‑indices ranging from 0.585 to 0.668 and AUCs from 0.605 to 0.684 across time points. Performance tended to improve over time, with the highest C‑indices observed at 8 years (e.g., 0.667 for ADAS‑Cog13, 0.668 for ADAS‑Cog11 and RAVLT‑IR). Five-Year Calibration curves (Fig. 3) showed close alignment between predicted and observed survival probabilities for all cognitive measures, supporting good calibration. Furthermore, residual‑versus‑fitted plots (Fig. 4 and 5) displayed no systematic patterns, indicating that model assumptions were adequately met and estimates were stable.

**Table 4:** Predictive performance of seven joint models.

Model	2 year		5 year		8 year	
	C-index	AUC	C-index	AUC	C-index	AUC
ADAS-Cog13	0.587	0.607	0.655	0.669	0.667	0.626
ADAS-Cog11	0.595	0.609	0.654	0.684	0.668	0.633
CDR-SB	0.594	0.607	0.653	0.684	0.667	0.631
FAQ	0.592	0.609	0.653	0.682	0.667	0.631
RAVLT-IR	0.585	0.605	0.654	0.677	0.668	0.629
RAVLT-L	0.596	0.615	0.653	0.676	0.666	0.638
MMSE	0.593	0.610	0.654	0.683	0.668	0.634

## Discussion

Using an updated ADNI cohort and a comprehensive joint modeling framework, this study identified key factors associated with the risk of progression from MCI to AD. Univariate and multivariate analyses consistently showed that APOE‑ε4 carrier status, older age, and longitudinal trajectories of poorer performance on functional and global cognitive measures (CDR‑SB, FAQ, ADAS‑Cog) significantly increased the hazard of conversion. In contrast, higher scores on memory (RAVLT) and global cognition (MMSE) were protective. Notably, the strength of association varied across cognitive domains, with CDR‑SB and FAQ exhibiting the highest hazard ratios per standard‑deviation change, underscoring their sensitivity to clinically meaningful decline^27,28^.

Our subgroup analysis further highlighted the dose‑dependent effect of APOE‑ε4 allele count on both conversion timing and the slope of cognitive decline, particularly for CDR‑SB and FAQ^29,30^. The incorporation of B‑splines allowed us to capture nonlinear temporal dynamics in these trajectories, and the explicit annotation of significance levels in the figures provides a transparent view of when and how these measures change across disease stages.

Methodologically, the joint models demonstrated high reliability, with intraclass correlation coefficients above 0.94 for all measures, indicating that most variance stemmed from stable between‑subject differences. Predictive accuracy, assessed via time‑dependent C‑indices and AUCs, was moderate to good over 2, 5, and 8 year horizons, and calibration curves showed close agreement between predicted and observed survival. These results support the robustness and clinical applicability of the modeling approach^31,32^.

Several limitations should be acknowledged. First, the analysis relied solely on neuropsychological and functional assessments; incorporation of neuroimaging or fluid biomarkers (e.g., amyloid‑PET, plasma p‑tau) could further enhance predictive precision^33,34^. Second, although the sample size was substantially increased relative to our initial analysis, the number of homozygous APOE‑ε4 carriers remains relatively small, and estimates in this subgroup should be interpreted with caution^35,36^. Finally, the models were developed and validated within the ADNI cohort; external validation in independent, more diverse populations is needed to ensure generalizability.

Future studies should aim to integrate multimodal data—combining cognitive, functional, imaging, and biomarker measures—into composite indices that may improve risk stratification and enable more personalized prognostic assessments^37,38^. Furthermore, applying similar joint modeling techniques in interventional cohorts could help evaluate whether changes in longitudinal trajectories serve as sensitive endpoints for detecting treatment effects^39,40^.

## Conclusion

In this updated analysis of a well‑characterized MCI cohort, we demonstrated that a semi‑parametric joint modeling approach effectively captures the nonlinear longitudinal trajectories of cognitive and functional measures and reliably predicts the risk of progression to AD. CDR‑SB and FAQ consistently showed the strongest associations with conversion hazard, outperforming other cognitive scales in both effect size and inter‑subgroup discrimination. APOE‑ε4 allele count exhibited a clear dose‑dependent relationship with earlier conversion and steeper cognitive decline. The models displayed robust internal validity, with good discriminative accuracy over follow‑up periods up to 8 years and excellent calibration. These findings support the prioritization of APOE‑ε4 genotyping, together with longitudinal monitoring of CDR‑SB and FAQ, in clinical risk stratification and personalized management of patients with MCI.

## Supplementary Information

Below is the link to the electronic supplementary material.

Supplementary Material 1

Supplementary Material 2

Supplementary Material 3

Supplementary Material 4

Supplementary Material 5

Supplementary Material 6

Supplementary Material 7

## Data Availability

The data supporting the findings of this study are openly available in the [ADNI] database. https://adni.loni.usc.edu/

## Abbreviations

Aβ: Amyloid-β
AD: Alzheimer’s disease
ADAS-Cog: Alzheimer’s disease assessment scale—cognitive
ADNI: Alzheimer’s disease neuroimaging initiative
AIC: Akaike information criterion
BIC: Bayesian information criterion
CDR-SB: Clinical dementia rating sum of boxes
CSF: Cerebrospinal fluid
FAQ: Functional activities questionnaire
ICC: Intraclass correlation coefficient
IQR: Interquartile range
logLik: Log-likelihood
MCI: Mild cognitive impairment
MMSE: Mini-mental state examination
RAVLT: Rey auditory verbal learning test
