# Supplementary figures and images for: Sensitivity comparison of longitudinal cognitive function indicators of Alzheimer’s disease after mild cognitive impairment: a prospective cohort study
Source: Sci Rep. 2026 Mar 22;16:14503. doi: 10.1038/s41598-026-44192-2 (PMC13149985; doi:10.1038/s41598-026-44192-2)

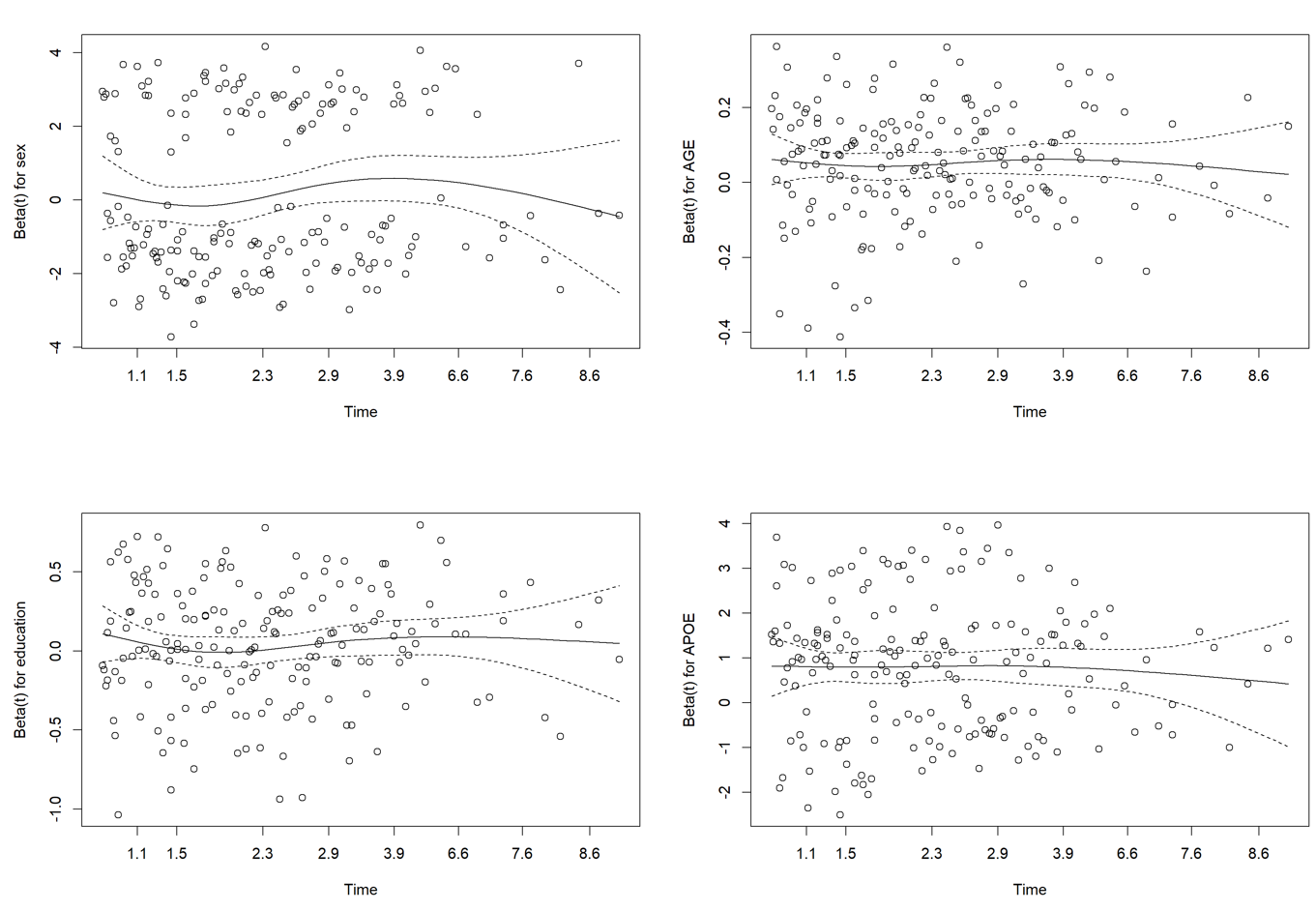

Supplement: Supplementary file 2 — Supplementary Material 2 [file 41598_2026_44192_MOESM2_ESM.png]

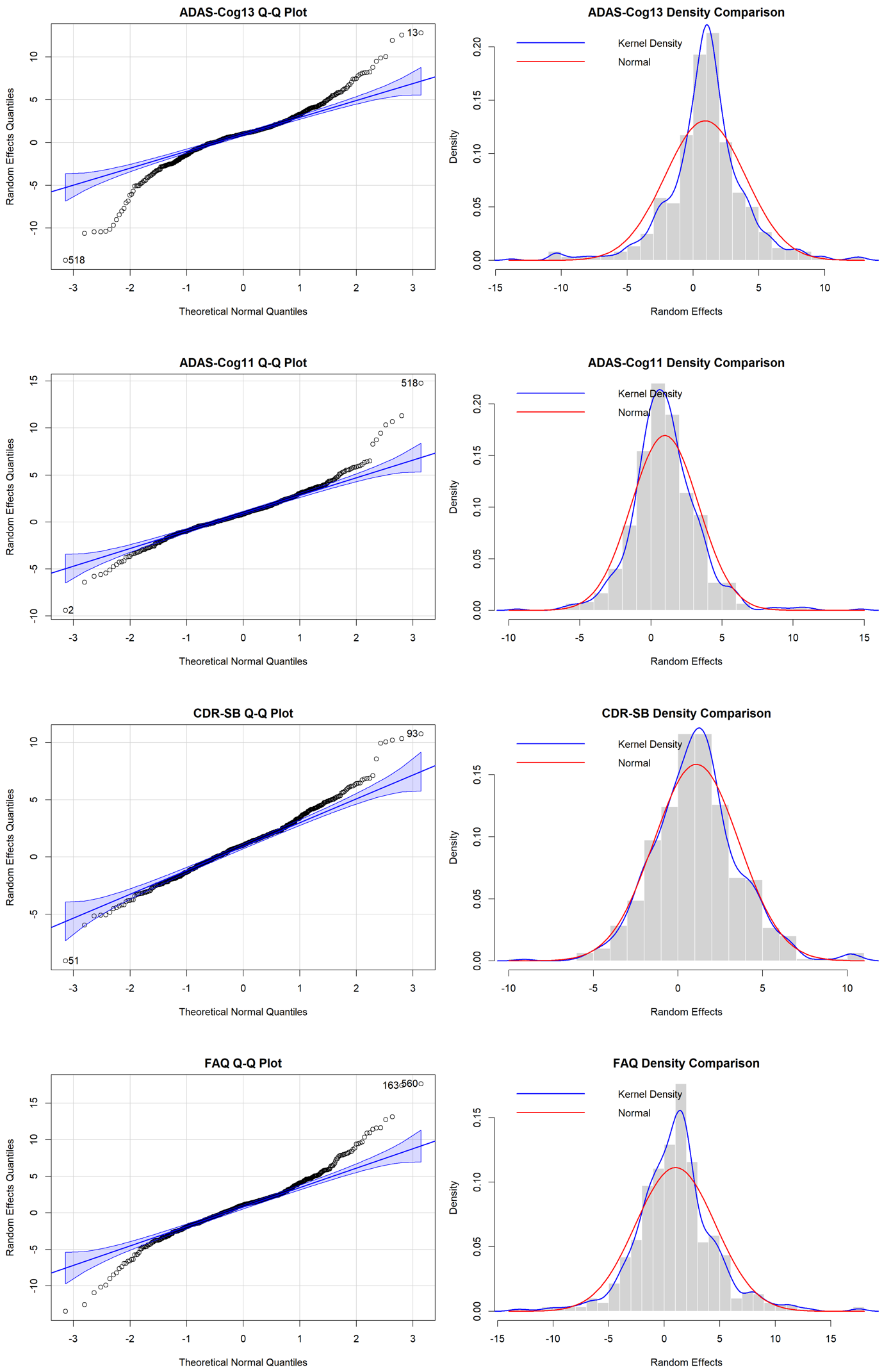

Supplement: Supplementary file 3 — Supplementary Material 3 [file 41598_2026_44192_MOESM3_ESM.png]

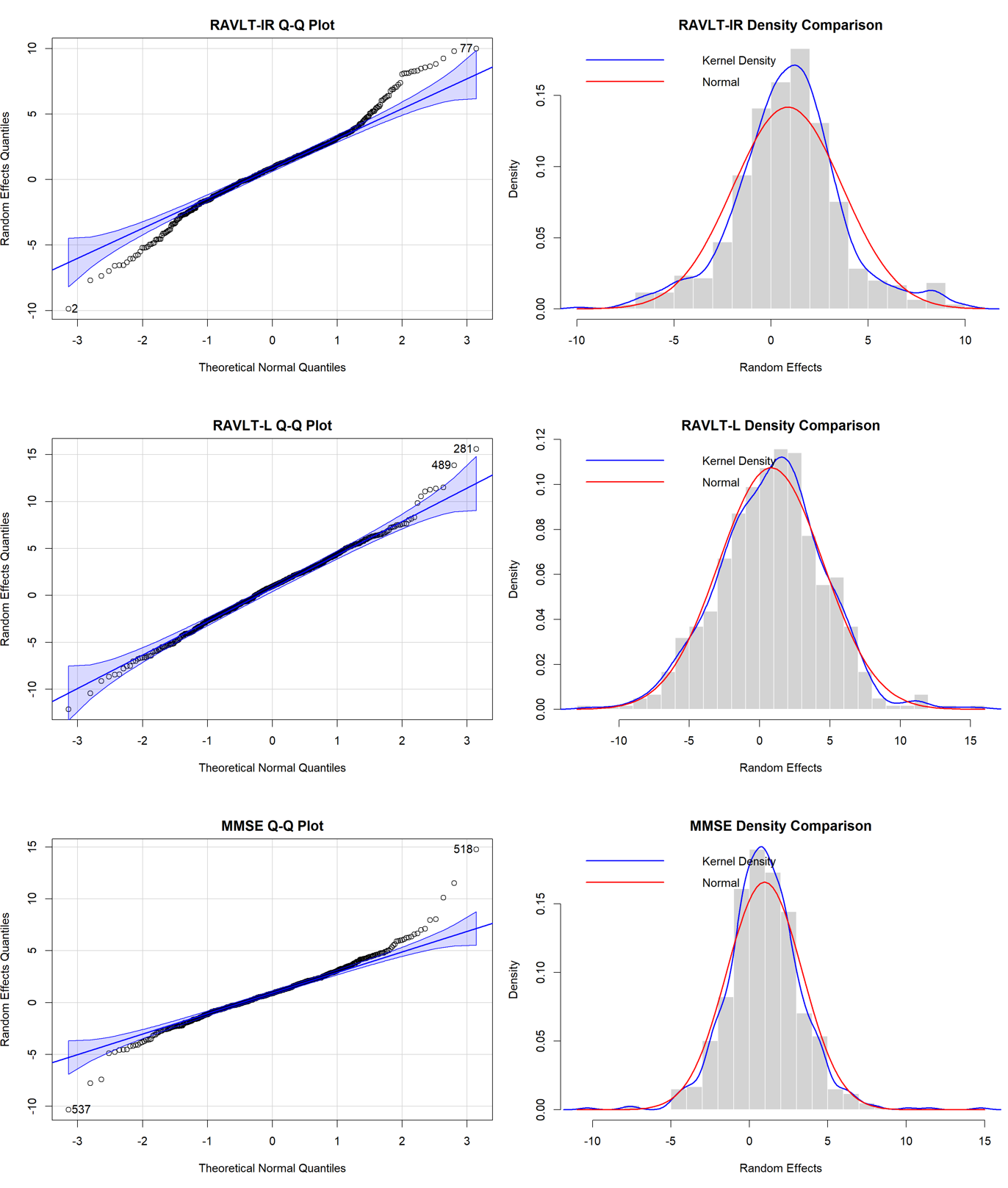

Supplement: Supplementary file 4 — Supplementary Material 4 [file 41598_2026_44192_MOESM4_ESM.png]
