# Supplementary material for: Sensitivity comparison of longitudinal cognitive function indicators of Alzheimer’s disease after mild cognitive impairment: a prospective cohort study
Source: Sci Rep. 2026 Mar 22;16:14503. doi: 10.1038/s41598-026-44192-2 (PMC13149985; doi:10.1038/s41598-026-44192-2)
Supplement: Supplementary file 7 — Supplementary Material 7 [file 41598_2026_44192_MOESM7_ESM.pdf]

Received: 24 April 2023 | Revised: 27 July 2023 | Accepted: 1 August 2023

DOI: 10.1002/alz.13449

Alzheimer's & Dementia®
THE JOURNAL OF THE ALZHEIMER'S ASSOCIATION

REVIEW ARTICLE

The Alzheimer's Disease Neuroimaging Initiative in the era of Alzheimer's disease treatment: A review of ADNI studies from 2021 to 2022

Dallas P. Veitch[1,2] | Michael W. Weiner[2,3,4,5,6] | Melanie Miller[1,2] | Paul S. Aisen[7] | Miriam A. Ashford[1] | Laurel A. Beckett[8] | Robert C. Green[9] | Danielle Harvey[8] | Clifford R. Jack Jr.[10] | William Jagust[11] | Susan M. Landau[11] | John C. Morris[12,13,14] | Kwangsik T. Nho[15,16] | Rachel Nosheny[2,5] | Ozioma Okonkwo[17] | Richard J. Perrin[12,13,14] | Ronald C. Petersen[18] | Monica Rivera Mindt[19,20] | Andrew Saykin[15,21] | Leslie M. Shaw[22] | Arthur W. Toga[23] | Duygu Tosun[2,3] | for the Alzheimer's Disease Neuroimaging Initiative

Correspondence
Michael W. Weiner, 4150 Clement St., San Francisco, CA 94121, USA.
Email: michael.weiner@ucsf.edu

Funding information
NIH, Grant/Award Number: U19 -AG 024904; National Institute on Aging

Abstract

The Alzheimer's Disease Neuroimaging Initiative (ADNI) aims to improve Alzheimer's disease (AD) clinical trials. Since 2006, ADNI has shared clinical, neuroimaging, and cognitive data, and biofluid samples. We used conventional search methods to identify 1459 publications from 2021 to 2022 using ADNI data/samples and reviewed 291 impactful studies. This review details how ADNI studies improved disease progression understanding and clinical trial efficiency. Advances in subject selection, detection of treatment effects, harmonization, and modeling improved clinical trials and plasma biomarkers like phosphorylated tau showed promise for clinical use. Biomarkers of amyloid beta, tau, neurodegeneration, inflammation, and others were prognostic with individualized prediction algorithms available online. Studies supported the amyloid cascade, emphasized the importance of neuroinflammation, and detailed widespread heterogeneity in disease, linked to genetic and vascular risk, co-pathologies, sex, and resilience. Biological subtypes were consistently observed. Generalizability of ADNI results is limited by lack of cohort diversity, an issue ADNI-4 aims to address by enrolling a diverse cohort.

KEYWORDS
Alzheimer's disease clinical trials, Alzheimer's Disease Neuroimaging Initiative, Alzheimer's disease subtypes, amyloid, cerebrovascular disease, co-pathologies, diagnosis, disease progression, generalizability, neurodegeneration, neuroinflammation, plasma biomarkers, prediction, resilience, tau

This is an open access article under the terms of the Creative Commons Attribution-NonCommercial License, which permits use, distribution and reproduction in any medium, provided the original work is properly cited and is not used for commercial purposes.
© 2023 The Authors. *Alzheimer's & Dementia* published by Wiley Periodicals LLC on behalf of Alzheimer's Association.

1 | INTRODUCTION

As the Alzheimer's Disease Neuroimaging Initiative (ADNI) enters its fifth phase and 19th year, the field of Alzheimer's disease (AD), for the first time, stands on the cusp of a treatment era, finally offering hope to millions of individuals and families suffering from AD. The contribution of ADNI to reaching this point cannot be overstated. ADNI is an observational study designed like a longitudinal clinical trial, and since its founding in 2004[1] has enrolled and followed 868 cognitively unimpaired (CU) participants, 1090 with mild cognitive impairment (MCI), and 408 with dementia diagnosed as AD. ADNI's overall goal has been to provide data for the design of clinical AD trials and to validate biomarkers for such trials. Pharmaceutical companies that have used ADNI data to help design and statistically power their trials include Biogen (aducanumab), Eisai (lecanemab), Merck (verubecestat), Lilly (solanezumab, donanemab), Genentech (crenezumab), and Roche (gantenerumab).

Many of ADNI's contributions to the development of these therapies have resulted from studies explicitly designed to enhance clinical trials. However, the availability of ADNI data to scientists worldwide without embargo (http://adni.loni.usc.edu/data-samples/access-data/), together with sharing of biospecimens (cerebrospinal fluid [CSF], blood, urine, and brain tissue), has enabled many studies that have augmented our understanding of the complexities of disease progression, and identified additional potential therapeutic targets. Additionally, ADNI data have been increasingly used in fields outside of AD, contributing to studies of Parkinson's disease (PD),[2] primary tauopathies,[3] coronary artery disease,[4] traumatic brain injury,[5] depression,[6] and brain changes over the human lifespan,[7] among others. One limitation of the open availability of ADNI data is that multiple statistical analyses may be performed on the same data, which raises concerns regarding multiplicity. However, there is no obvious solution to this problem.

As of the end of 2022, there have been > 4.8 million downloads of ADNI clinical data and > 300 million downloads of ADNI imaging data. More than 5000 "ADNI studies" (defined as those using ADNI data and/or samples, either as a primary cohort, part of a larger consortium, or as a replication cohort) have been published thus far and reviewed to the end of 2020.[8–13] All ADNI studies are searchable at https://adni.loni.usc.edu/news-publications/publications/.

In 2021 and 2022, 1459 ADNI studies were published (see supporting information for a complete list), representing a period of unprecedented productivity and impact spanning the COVID-19 pandemic. During this time, the ADNI-3 study[14] drew to a close, and ADNI transitioned to the latest study, ADNI-4,[15] funded entirely by the National Institutes of Health. This review covers 291 selected impactful ADNI studies from this time frame. Studies in which ADNI data and/or samples have been used as part of a larger cohort or meta-analysis, or in which ADNI serves as a discovery or replication cohort, are noted. The review is divided into three sections that reflect ADNI's overarching goals. Section 2 examines how ADNI data have directly contributed to the development of AD therapies, describes important data harmonization efforts, outlines progress in the development of plasma biomarkers for clinical use, and reviews diagnostic and prognostic models. Section 3 describes how ADNI studies have contributed to an increasingly nuanced understanding of mechanisms underlying AD disease progression. While the National Institute on Aging–Alzheimer's Association (NIA–AA) AT(N) research framework for the biological definition of AD[16] is based on biomarkers for amyloid beta (Aβ) deposition (A), pathologic tau (T), and neurodegeneration (N), these studies paint a picture of AD as a complex multifactorial disease that is not part of normal aging, and that commonly coexists with multiple pathologies, resulting in the observed heterogeneity in disease course. The deeper understanding of the biology of AD resulting from recent ADNI studies reveals more potential therapeutic targets and suggests the need for multiple simultaneous therapies. It is important to note that the generalizability of the studies may be limited by the lack of ethnocultural and educational diversity of the ADNI cohort. Section 4 addresses these limitations and describes how ADNI-4 plans to overcome them.

RESEARCH IN CONTEXT

1. **Systematic review**: The authors identified 1459 journal publications using Alzheimer's Disease Neuroimaging Initiative (ADNI) data/samples from 2021 to 2022 using traditional search methods.
2. **Interpretation**: ADNI studies improved subject selection, modeling, and detection of treatment effects for clinical trials, and described harmonization methods. ADNI samples contributed to the development of plasma biomarkers such as phosphorylated tau for clinical use, and described prognostic abilities of amyloid beta, tau, neurodegeneration, and inflammation biomarkers. ADNI studies supported the amyloid cascade sequence of disease progression and detailed how genetic and vascular risk, co-pathologies, resilience, and sex contribute to heterogeneity and biological subtypes. Results may not be generalizable due to the limited cohort diversity.
3. **Future directions**: The ADNI-4 cohort, currently enrolling, will be more diverse to ensure generalizability of results. In the age of Alzheimer's disease (AD) treatment, ADNI will continue to improve AD clinical trials and provide data for the development of personalized medicine approaches.

2 | ADNI'S CONTRIBUTIONS TO THE TREATMENT, DIAGNOSIS, AND PREDICTION OF AD

2.1 | Studies of existing and developing therapies

Even though ADNI is a non-treatment, observational study, ADNI data have been used to assess the effects of established medications for AD, the use of medications targeting risk factors for AD,

and disease-modifying therapies under development. The following reports should be interpreted with caution because they are not from randomized placebo-controlled trials. Cholinesterase inhibitors that enhance cholinergic neurotransmission (donepezil, rivastigmine, and galantamine) were for many years the only treatment option for AD symptoms. Lower longitudinal tau deposition in early Braak stages was observed in Aβ positive (A+) participants taking cholinesterase inhibitors. The authors suggested a neuroprotective effect of these medications.[17] Non-demented users of anticholinergic medications, which are used to treat a variety of other conditions and have the opposite effect to cholinesterase inhibitors, suffered more cognitive impairment than non-users.[18] This was partially mediated by an effect of gray matter (GM) density and functional connectivity in the nucleus basalis of Meynert.[18] The authors suggested that the results support a hypocholinergic mechanism underlying cognitive decline. In contrast, a third study reported that long-term donepezil treatment in MCI participants was associated with greater regional Aβ and tau load with concomitant worse cognitive performance.[19] This result could be due to the selection of more impaired patients with greater Aβ and tau load for treatment with donepezil long term. The greater atrophy and hypometabolism observed in acetylcholinesterase inhibitor users compared to non-users did not survive adjustment for baseline differences, likely due to a greater rate of prescription of these medications to more impaired patients rather than a drug effect.[20]

There has been recent interest in repurposing drugs for treatment of AD risk factors such as hypertension, inflammation, and type II diabetes mellitus (T2DM) as therapies for AD. Several ADNI studies have investigated the effects of these drugs on AD disease progression. The use of angiotensin receptor blockers for the treatment of hypertension was associated with a lower risk of progression to AD dementia in MCI participants compared to other or no anti-hypertensive medications.[21] Similarly, aspirin use was associated with slower decline in the Mini-Mental State Examination (MMSE) in participants with AD dementia[22] suggesting, to the authors, a neuroprotective effect. T2DM in MCI participants was associated with worse cognition and lower brain volumes but treatment with metformin attenuated these effects, likely via a glycemia-independent mechanism.[23] Finally, selective serotonin reuptake inhibitors for the treatment of depression were not found to have any beneficial effects on cognition or Aβ load in ADNI participants.[24] Results from these observational, non-randomized studies may suggest future randomized trials to assess the benefits of these treatments.

With the lecanemab phase 3 trial results[25] and subsequent accelerated US Food and Drug Administration (FDA) approval, in addition to prior accelerated FDA approval of aducanumab in 2021, anti-Aβ therapies have been demonstrated to have a significant effect in not only clearing Aβ plaques, but in slowing cognitive decline. Knowledge of relationships between AD biomarkers and clinical presentation determined from ADNI data formed the basis of a model of the long-term health outcomes of lecanemab.[26] The model, when applied to phase 2 lecanemab trial data, estimated that long-term use of lecanemab in MCI participants would delay time to progression to AD dementia by $\approx$ 2.5 years, and increased time in mild AD while decreasing time in moderate and severe AD and in institutional care (Figure S1 in supporting information).

2.2 | Methodological improvements to clinical trials

To date, the effects on cognition of Aβ-modifying disease therapies have been at best modest.[25] ADNI studies have pointed to potential explanations for the discrepancy between these results and the body of evidence supporting a central role for Aβ in disease progression. Targeting of CSF Aβ42 alone may miss the contribution of different Aβ isoforms.[27] Higher levels of CSF Aβ38 were associated with a lower risk of conversion from MCI to AD dementia and slower cognitive decline in both the ADNI and Biomarkers for Identifying Neurodegenerative Disorders Early and Reliably (BioFINDER) cohorts, suggesting that other Aβ isoforms resulting from differential processing of the amyloid precursor protein (APP) also influence disease progression.[27]

Selection of participants for clinical trials based on Aβ status is increasingly common, but Aβ positivity does not necessarily identify those most likely to progress to biomarker-defined AD (A+T+ as per NIA-AA guidelines[28]). A study of A+ ADNI participants identified a substantial proportion of individuals whose tau positron emission tomography (PET) scans remained normal over 5 years.[29] These individuals declined more slowly and were characterized by having lower frequencies of the apolipoprotein E (*APOE*) ε4 allele, larger hippocampal volume, and lower Aβ PET Centiloid (CL) units,[30] suggesting that they can be identified from baseline characteristics. An alternative approach to identifying individuals likely to decline used a machine learning classifier developed from early post-injection 18F-florbetapir image frames. This was highly correlated with a [^{18}F] fluorodeoxyglucose (FDG) PET AD progression biomarker and with decline in global cognitive measures, outperforming region of interest (ROI) standardized uptake value ratio (SUVR).[31] Slow and fast decliners may also be detected using a "run-in period" without treatment to estimate rates of disease progression.[32]

Subject stratification may be improved by leveraging knowledge of MCI heterogeneity. Four distinct MCI clusters were detected using neuropsychological subscores.[33] These differed in impairments in specific cognitive domains and rate of progression to AD dementia. A subtype with the most impaired memory and worst orientation had the fastest progression to AD. The addition of comorbidities to non-comorbidity data (demographics, *APOE* ε4, magnetic resonance imaging [MRI] volumes, cognitive and functional tests, and plasma biomarkers) in cluster analysis improved the differential prognoses of identified subtypes. The comorbidities that contributed the most to defined subtypes were obesity, cardiovascular issues, hearing loss, and hypertension. In the subtype with the worst prognosis, no patients reverted to CU, and 60% converted to AD dementia over 5 years. Consideration of comorbidities may therefore enhance the probability of selecting participants who will progress to dementia within the trial time frame.

Detection of a treatment effect using cognitive decline as a primary endpoint may be made more difficult by heterogeneity in cognitive trajectories that results in unequal rates of cognitive decline between treatment and placebo groups. This may lead to underestimation or overestimation of the treatment effect.[32] A simulated clinical trial in ADNI[32] using inclusion criteria from recent anti-amyloid trials of MCI and mild AD confirmed heterogeneity in the rate of change of three cognitive endpoints (MMSE, Alzheimer's Disease Assessment Score–Cognitive subscale [ADAS-Cog], and Clinical Dementia Rating–Sum of Boxes [CDR-SB]) over up to 9 years. At 18 months, there was a wide range in effect sizes of simulated group differences with those from recent trials largely falling within the 95% range for all cognitive outcomes (Figure 1). Stratification based on tau positivity and *APOE* ε4 genotype resulted in a steeper decline in cognition in high-risk groups but even greater variability in progression. These results illustrate the difficulty in detecting a statistically significant treatment effect using cognitive outcomes and may help select a trial duration at which the treatment effect overcomes variability in disease progression.

Another source of heterogeneity in disease course is the differences in MCI diagnostic criteria between cohorts. ADNI data from MCI participants form the basis of the Placebo Group Simulation Approach, which aimed to decrease the number of study participants and placebo groups in clinical trials.[34] However, the development of more generalizable algorithms for this approach was precluded by differing MCI criteria, which resulted in variable disease progression across cohort studies, convenience samples, and a clinical drug trial.[34] Likewise, ADNI MCI participants differed from those from the National Alzheimer's Coordinating Center (NACC) Uniform Data Set in cognitive measures, most critically in prose memory tests (Wechsler Memory Scale Revised Story A in ADNI and Craft Story immediate recall in NACC).[35] Standardized MCI psychometric criteria would facilitate comparison of patient data from different sources by minimizing diagnostic heterogeneity, and would allow assessment of approaches such as the Placebo Group Simulation.

ADNI data have formed the basis of a modeling tool, SimulAD, designed to simulate interventions with disease-modifying drugs.[36] This tool uses relationships between ADNI multimodal imaging and clinical data to describe disease progression, forming the basis for simulation of a variety of anti-amyloid therapy intervention scenarios in preclinical patients. Modeling using SimulAD estimated that for an intervention that lowers Aβ load by 100%, > 80% power can be obtained with 1000 subjects per arm around 7 years before conversion from MCI to AD using a variety of clinical outcomes for equivalent power with fewer subjects per arm. For a goal of greater efficacy of Aβ removal, earlier intervention is needed (Figure S2 in supporting information). These results suggest that intervention in the preclinical phase before the pathological cascade is entrenched is critical to the success of anti-amyloid trials, and that recent anti-amyloid trials may have been consistently underpowered. However, the large sample sizes predicted to be required may not be practical. SimulAD may have utility as a multimodal enrichment tool and has recently been validated in a memory clinic sample.[37]

2.3 | Harmonization efforts

External validation of studies in independent cohorts is complicated by lack of harmonization of methods, lack of universal cut-points for biomarkers, and issues of data set accessibility. Harmonization of methods across different data sets is crucial for enabling comparison of data obtained using different methodologies, replicating results in research and clinical trials, and pooling data for increased statistical power. Quantitative Aβ or tau load determined by PET requires harmonization across sites, scanners, and tracers for comparison of data from multisite studies. A study of ADNI Aβ PET scans[38] determined transformation equations for the conversion of cross-sectional and longitudinal SUVRs from 18F-florbetapir or 18F-florbetaben PET scans to CL units to promote data harmonization. Additionally, it derived CL thresholds from both a young control sample (18 CL) and from ADNI (21 CL) that were consistent with the existing 18F-florbetaben threshold for Aβ positivity (20 CL). Using florbetaben, cut-points around this region, 13.5 CL and 35.7 CL, were found to bracket a "gray zone" of emerging Aβ pathology that is predictive of faster subsequent Aβ accumulation in a five-cohort study.[39] An alternative MRI-free index of Aβ load, AMYQ, was developed using Aβ PET scans and neuropathological data from ADNI and was in high agreement with CL measures across four different PET tracers.[40] The determination of tau positivity from PET scans differs between cohorts and radiotracers and depends on both the quantity and location of tracer retention. This variability can result in differing tau positivity rates that impact inclusion into clinical trials, staging methods, and more. A systematic review of 23 cohort studies[41] derived a tau PET cut-point that differentiated tau positive and negative groups on CSF phosphorylated tau (p-tau)181 and cognitive measures.

Determination of Aβ and tau status from CSF biomarkers is also complicated by variability in assay methods that precludes the use of universal cut-points. A method to standardize the procedure for cut-point determination in different cohorts was developed that relies on CSF p-tau181 levels to determine the cut-point values for CSF Aβ42 and CSF Aβ40.[42] The method was validated against Aβ and tau PET and tested across 11 cohorts including ADNI and may make selection of cut-points across cohorts a more transparent process. Neuropsychological batteries also vary between cohorts, which may complicate efforts to combine data in the quest for larger sample sizes. A novel approach developed in ADNI and the Australian Imaging, Biomarker & Lifestyle Flagship Study of Ageing (AIBL) used a machine learning algorithm to impute longitudinal neuropsychological test scores not used in one cohort to align with the other cohort, facilitating harmonization of the data sets.[43] A harmonized version of the widely used Preclinical Alzheimer Cognitive Composite (PACC) was developed using confirmatory factor analysis across ADNI, AIBL, and the Harvard Aging Brain Study (HABS), and outperformed the common standardized version in combined cohort analyses.[44]

Another barrier to external validation in independent cohorts is accessibility of the data set and compatibility of the variables within the data sets. An interactive tool that allows exploration of features

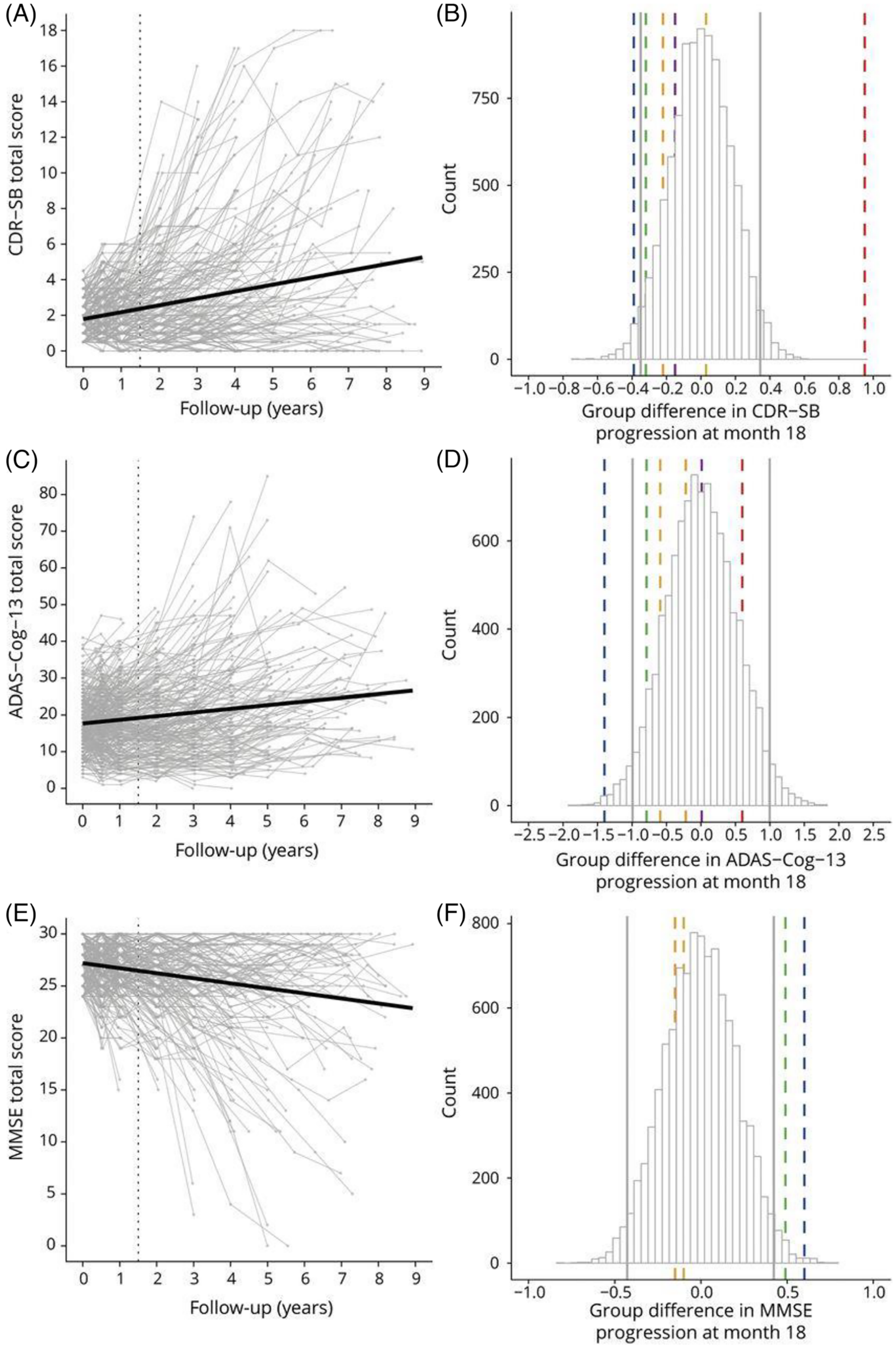

FIGURE 1 Rates of change of three cognitive outcomes in a simulated clinical trial in ADNI using inclusion criteria from anti-amyloid trials. Left column: individual trajectories on the (A) CDR-SB, (C) ADAS-Cog, and (E) MMSE. The vertical dotted lines represent scores at 18 months. Right: simulated group differences in change from baseline to month 18 based on the total sample ($n = 302$) on (B) CDR-SB, (D) ADAS-Cog, and (F) MMSE, including 95% range of effect sizes as indicated by vertical gray lines and effect sizes reported for recent clinical trials as indicated by vertical dashed colored lines (blue = EMERGE; yellow = ENGAGE; green = EXPEDITION-3; orange = DAYBREAK-ALZ; red = IDENTITY-2; magenta = BAPINEZUMAP). ADAS-Cog, Alzheimer's Disease Assessment Scale-Cognitive subscale; ADNI, Alzheimer's Disease Neuroimaging Initiative; CDR-SB, Clinical Dementia Rating Sum of Boxes; MMSE, Mini-Mental State Examination. Reproduced under open access from Jutten et al.[32]

of different data sets, AdataViewer, aims to increase the findability and interoperability of cohort data sets, saving time and effort for researchers in the field.[45] It allows researchers to access metadata from 20 cohort studies including ADNI, to select cohorts that contain their variables of interest, and to apply for data access (Figure S3 in supporting information).

2.4 | Blood biomarkers for AD

There has been a recent rapid development of blood-based biomarkers that reflect AD pathobiology for use in primary care and clinical trials. These biomarkers, assessed using ultrasensitive assay techniques, circumvent the issues of cost, invasiveness, and accessibility of PET and CSF biomarkers. Recent ADNI studies have focused on clinical assay development and validation, and retrospective longitudinal studies, two key steps toward clinical implementation.[46] These studies have primarily assessed AT(N) plasma biomarkers but also have reported novel blood-based biomarkers reflecting different aspects of disease progression. There has been extensive work in this area (recently reviewed in Balogun et al.[47]). The following covers only blood biomarker studies that either used ADNI samples or analyzed existing data from the ADNI database.

2.4.1 | Plasma Aβ

Two head-to-head studies[48,49] compared six candidate plasma Aβ assays, three immunoassays (Roche Elecsys Cobas e601, Adx NeuroSciences Simoa Neuro 4-plex E Kit, and Quanterix Simoa Aβ40 and Aβ42 Advantage Kit), and three mass spectrometry (MS)-based assays (Washington University immunoprecipitation [IP]-MS, Shimadzu IP-matrix-assisted laser desorption ionization time-of-flight-MS, and University of Gothenburg IP-MS assays). The first study,[48] which aimed to prioritize assays for more extensive study, identified the Roche, Washington University, and Shimadzu assays as able, in combination with age and *APOE* ε4 status, to improve prediction of Aβ PET status beyond a base model (age and *APOE* ε4 status) in participants across the AD spectrum. However, none of these assays reached the prespecified threshold for a clinical prescreening tool of an area under the receiver operating curve (AUC) of 0.90 with an increase of 0.15 in AUC over the base model. The best assay, the Washington University IP-MS, achieved an AUC of 0.842 compared to an AUC of 0.75 for the base model. The second study[49] also identified the Washington University and Roche assays as the best performing for predicting Aβ PET status in CU and MCI participants, and for discriminating between AD and CU participants. The Washington University IP-MS plasma Aβ42/40 assay, now available commercially as Precivity AD (C_2N Diagnostics), was validated in ADNI, AIBL, and BioFINDER.[50] Combined with *APOE* ε4 status, it predicted Aβ PET status with an AUC of 0.88 and CSF Aβ status with an AUC of 0.93 in the combined cohort. Prescreening with this assay was estimated to decrease the number of Aβ PET scans by up to 62% and 19% for the enrollment of CU and MCI participants, respectively, into a clinical trial, potentially reducing screening costs 10-fold.[50]

2.4.2 | Plasma phosphorylated tau

Recent ADNI studies have explored the ability of plasma p-tau to predict not only Aβ status but also markers of disease progression. A replication study of plasma p-tau181[51] reported that a longitudinal increase in levels of this biomarker was associated with worse AD biomarkers (cortical Aβ accumulation, hypometabolism, atrophy, cognitive decline, and CSF Aβ42, p-tau181, and total tau [t-tau]) in ADNI participants across the AD spectrum and that the association was strongest in A+ participants (Figure S4 in supporting information). In similar studies, baseline plasma p-tau181 was associated with CSF Aβ42,[52] CSF p-tau181,[52,53] and CSF t-tau;[52] 18F-florbetapir PET;[53,54] 18F-flortaucipir PET;[54] regional hypometabolism;[53,54] gray matter (GM) atrophy in CU and cognitively impaired (CI) participants;[55] white matter (WM) volume in MCI participants;[52,55] cognitive measures;[52,56,57] progression from CU and MCI;[52,56] time to dementia diagnosis;[57] and Braak neurofibrillary tangle (NFT) stages at autopsy.[58] Plasma p-tau181 was additionally associated with a polygenic risk score (PRS) containing *APOE* in all diagnostic groups and in both A+ and A– participants, and with a non-*APOE* PRS in MCI and A+ participants only.[59]

These studies provide strong evidence for the utility of plasma p-tau181 as a biomarker of the full panoply of AD characteristics. Levels of this biomarker increased across diagnostic categories,[60,52,53] and differed significantly between CU and AD participants across all age ranges,[60] indicating that it may be a clinically useful diagnostic test. Plasma p-tau181 additionally distinguished between pathology-confirmed AD and A– CU[57,58] and between pathology-confirmed AD and non-AD dementia with comparable performance to CSF p-tau181 (Figure 2).[57,58] Combined with *APOE* ε4 status, memory, and executive function, plasma p-tau181 predicted progression from MCI to AD dementia within 2 years in an ADNI validation cohort with an AUC of 0.90 achieving very similar results to the model developed in BioFINDER using plasma p-tau217.[61] These models performed similarly to those using CSF biomarkers and outperformed clinical predictions by memory clinic physicians (AUC 0.72), illustrating the vast potential of plasma biomarkers to transform clinical diagnosis and prediction.

Investigators are beginning to focus attention on the definition and utilities of cut-point determination. For example, in one study, a cut-point based on Youdin balanced sensitivity and specificity was determined in AD dementia (autopsy diagnosis) versus CU (A– by PET) in the University of Pennsylvania (UPenn) Alzheimer's Disease Research Center (ADRC) cohort and applied to an ADNI MCI replication sample showing that values above cut-point were associated with faster rate of decline in MMSE, shorter time to functional decline, and conversion to dementia.[57] Much more work in this area is needed especially across underserved minorities and those with comorbidities.

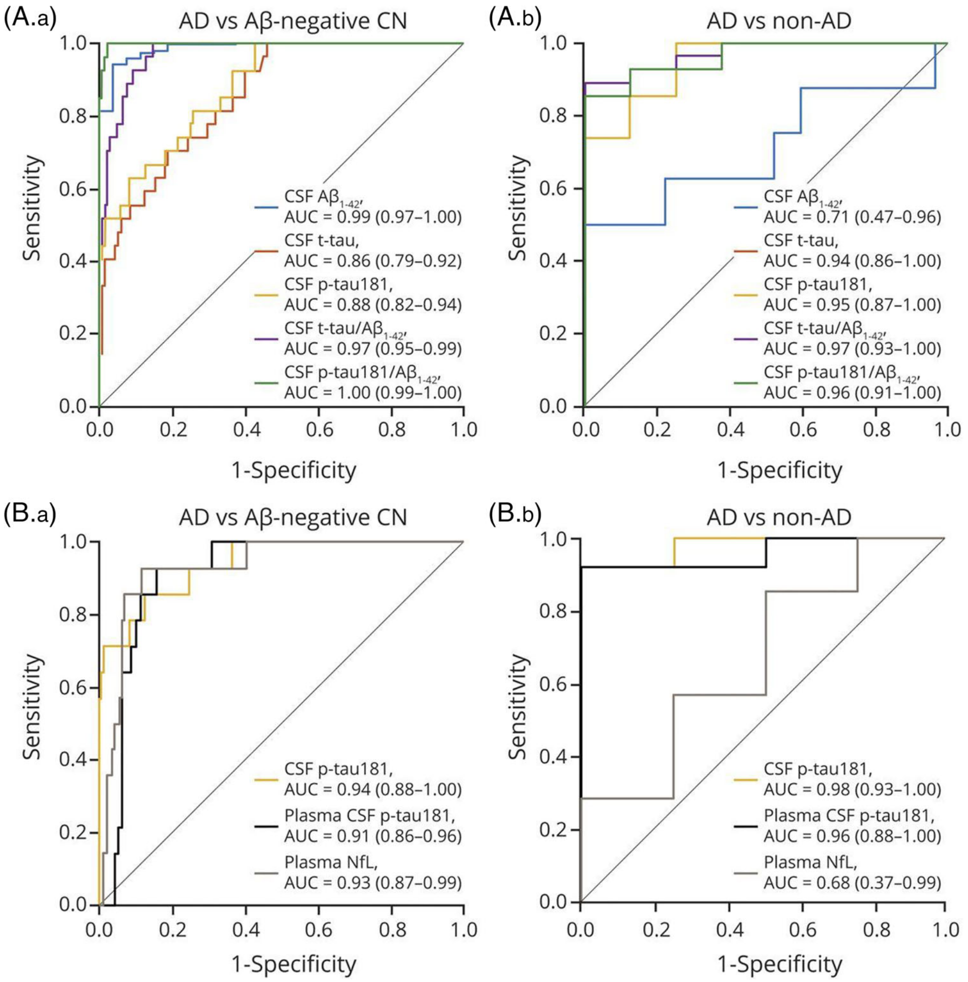

FIGURE 2 ROC curves for distinguishing pathology-confirmed AD dementia from non-AD dementia and Aβ-PET–negative healthy controls. ROC curves showing the performance of (A) Elecsys CSF biomarkers and (B) plasma biomarkers compared to CSF tau phosphorylated at threonine 181 (p-tau181) for the discrimination of pathology-confirmed AD dementia from (A.a and B.a) Aβ-PET–negative healthy controls and (A.b and B.b) non-AD dementia. AUC and 95% confidence interval are reported in the inset of each panel. Aβ, amyloid beta; AD, Alzheimer disease; AUC, areas under the curve; CN, cognitively normal; CSF, cerebrospinal fluid; NfL, neurofilament light; PET, positron emission tomography; ROC, receiver operating characteristic; T-tau, total tau. Reproduced under open access from Grothe et al.[58]

Although p-tau181 provides very useful information, reports during the past 2 years suggest that p-tau217 has a greater dynamic range than p-tau181 and greater accuracy to predict Aβ positivity and cognitive decline.[62–64] At the time of this review there have been no p-tau217 analyses of ADNI samples.

2.4.3 | Plasma neurofilament light

Plasma biomarkers of neurodegeneration may have utility in AT(N) studies, for predicting cognitive decline, and for assessing the rate of disease progression. Plasma neurofilament light (NfL), considered a non-specific marker of neuronal injury, is the most studied plasma biomarker in this category. It outperformed plasma t-tau in the prediction of atrophy and cognitive decline in a head-to-head comparison in the Mayo Clinic Study of Aging (MCSA) with replication in ADNI.[65] In ADNI CU and MCI participants, plasma NfL was cross-sectionally associated with hippocampal volume and a range of cognitive measures, and longitudinally with hippocampal atrophy and decline in Logical Memory-Immediate Recall and ADAS-Cog13; no associations were found cross-sectionally and longitudinally for plasma tau.[65] Similarly, elevated plasma NfL at baseline predicted greater decline in function as well as cognition in ADNI MCI participants, and also predicted greater decline in the PACC scores in CU participants with subjective subtle cognitive decline[66] (Figure S5 in supporting information). In CU participants, elevated baseline plasma NfL was associated with worse measures of cerebral small vessel disease burden (lacunar infarcts, WM hyperintensities [WMH], and cerebral microbleeds), and rate of change in this measure predicted progression in cerebral small vessel disease burden.[67] A similar association was found with lacunar infarcts in the MCSA cohort but this was not replicated in ADNI,[65] so the utility of plasma NfL as a biomarker of cerebral small vessel disease has yet to be confirmed.

How does the predictive ability of plasma NfL as a non-specific marker of neurodegeneration compare to that of plasma p-tau181 as a marker of AD pathology across the AD spectrum? A head-to-head study using ADNI longitudinal data[68] reported that while both biomarkers predicted glucose hypometabolism, atrophy, and cognitive decline, plasma p-tau181 was associated with hypometabolism and atrophy in AD-typical regions in A+ participants only, whereas plasma NfL was associated with non-specific neurodegeneration in an Aβ-independent manner (Figure S6 in supporting information). The associations were stronger in CI than CU individuals. Similarly, elevated baseline plasma p-tau181 predicted memory decline only in A+ ADNI participants whereas plasma NfL predicted memory decline regardless of Aβ status.[69]

2.4.4 | Other blood-based biomarkers

The current AT(N) plasma biomarkers do not reflect elements of the disease process occurring between tau deposition and neurodegeneration (described in Section 3.2). Synaptic dysfunction, reflecting the loss of communication across the synapses or actual loss of neuronal synapses, occurs before neuronal death, and represents a common point at which pathological processes in AD and other dementias converge before neurodegeneration and cognitive decline. The importance of synaptic dysfunction in the disease process is reflected in the number of therapies under development aimed at preserving synaptic function, such as those targeting glutamate receptors.[70] A marker of synaptic dysfunction is therefore desirable for assessing target engagement of these medications in addition to more finely tracking disease progression. The N-methyl D aspartate receptor 2A, the therapeutic target of memantine, is involved in synaptic function, and can be measured in blood extracellular vesicles (EVs) derived from the brain.[71] Numbers of EVs carrying this receptor were lower in AD compared to CU, and also compared to PD in samples from the Pacific Northwest Udall Center of Excellence in PD Research, suggesting specificity for AD. A model containing these EVs discriminated between AD and CU with AUCs of 0.91 in the exploratory cohort and 0.81 in the ADNI external validation cohort. The N-methyl D aspartate receptor 2A measured in brain-derived EVs may therefore be a useful biomarker of neuronal synaptic loss.[71]

An alternative blood biomarker for AD is based on consistent differences in DNA methylation between AD and CU participants. Distinguishing epigenetic marks were identified by meta-analysis of epigenome-wide association studies from AIBL and ADNI.[72] Five significant CpG sites of methylation were identified, including one in the *FKBP5* gene involved in the promotion of tau protein aggregation, and several biologically relevant promoter-associated CpG island regions. A model comprising age, sex, immune cell type proportions, and a methylation risk score based on significant CpG sites discriminated between AD and CU participants with an AUC of 0.696 in an external validation cohort (AddNeuroMed). As age is the greatest risk factor for AD, and DNA methylation changes with age, the study highlights the potential of methylated DNA as a source of AD biomarkers.

2.4.5 | Blood biomarkers conclusions

Ultrasensitive assay techniques have allowed development of plasma biomarkers that predict disease progression with accuracy comparable to CSF biomarkers. They therefore have the potential to revolutionize clinical diagnosis and prediction. However further hurdles remain before implementation in primary care: (1) validation in large, ethnically diverse primary care populations; (2) development of clinical grade assays; (3) standardization of protocols; (4) determination of cut-points and application in prospective studies; and (5) determination of the impact of chronic comorbid conditions. ADNI will continue to play a part in the development and validation of plasma biomarkers.

2.5 | Diagnosis and prediction approaches

ADNI data, freely available to qualified researchers worldwide, have been used for the development of many of the diagnostic and prognostic methods in AD. For instance, 56% of studies included in a review of the use of artificial neural networks to diagnose AD from brain imaging scans used ADNI data.[73] A number of systematic reviews of models predicting MCI progression to AD dementia reported that ADNI data were used in 67%,[74] 85%,[75,76] and 92%[77] of included studies. The number of articles, the number of participants per article, and AUCs have all increased over time[76] (Figure S7 in supporting information). This can be considered both a resounding success of data-sharing in ADNI, but also as a huge limitation to the interpretation of these studies, given the lack of diversity of ADNI's participants and its strict inclusion and exclusion criteria thus far. Furthermore, few diagnostic and prognostic methods have been externally validated in independent cohorts to ensure generalizability, and there is considerable variability in statistical methodologies and data presentation among studies.[74,76,77] This review will therefore highlight only studies of diagnostic and prognostic approaches notable for their thoroughness, novelty, and potential for clinical use, and otherwise present general findings from systematic reviews.

2.5.1 | Systematic reviews of prediction of progression from MCI to AD dementia

Prediction of progression from MCI to AD dementia is a much greater classification challenge than discriminating between AD and CU patients. Prediction of clinical trajectories of MCI participants is complicated by the heterogeneity in rates of progression, even within those who are A+. This hampers the ability of secondary prevention trials to detect cognitive outcomes within reasonable time frames; therefore, the development of methods to circumvent this difficulty is paramount. Many ADNI studies have used machine learning to select variables and construct predictive models. A systematic review[77] of 116 such studies from 2010 to 2021, the majority (92%) using ADNI data, reported that the most common features used were whole brain volumes (60%), glucose metabolism (27%), neuropsychological tests

(16%), *APOE* ε4 genotype (14%), and demographics (13%). Around half of studies used support vector machines (SVM) as their machine learning method, with the remainder using other methods such as random forest and neural networks. Models were most commonly cross-validated (72/116), but only three were externally validated.

A second systematic review of 111 studies from 2010 to 2018 (comprising 85% ADNI studies)[76] also reported structural MRI measures were the most common features (69%), followed by neuropsychological variables (43%), demographics (34%), and FDG PET (20%; it should be noted that few studies used Aβ and tau PET within the time frame of these systematic reviews, but methods using these modalities have since been developed). In a performance analysis of ADNI cohort experiments, only T1 MRI ROIs, FDG PET features, and domain targeted cognitive features significantly impacted AUC, with FDG PET features outperforming MRI features. The most common machine learning algorithm was SVM, but the use of neural networks increased from 2016 to 2018.

These reviews found little difference between the accuracies achieved using different machine learning methods, although convolutional neural networks (CNN) have a slight performance advantage and are more frequently used in recent studies.[77] Both reviews reported that the mean accuracy of predictive models converged at ≈ 75% as the number of participants increased.

2.5.2 | Aβ-based approaches

Although baseline CSF Aβ42 was not a strong predictor of cognitive decline in A+ subjects,[78] other Aβ measures performed better. Regional patterns of Aβ burden, particularly in the precuneus and parietal cortex, together with sex and *APOE* ε4 status, better predicted progression from CU to MCI, and from MCI to AD dementia over 1 and 3 years than global Aβ burden or CSF biomarkers.[79] Subthreshold Aβ deposition in similar regions also predicted conversion of amnestic MCI participants to AD dementia.[80] CL thresholds for predicting future decline in CU participants in the PACC score calculated in three independent cohorts including ADNI ranged from 15 to 18.5 CL compared to cut-points for distinguishing between A+ and A– CU individuals, which ranged from 19.0 CL to 25.7 CL.[81] This suggests there is an inflection point below established thresholds for Aβ positivity at which cognitive decline increases significantly. Accumulating regional Aβ below positivity thresholds may therefore be meaningful in disease progression and should be taken into consideration in clinical trial design.

2.5.3 | Inflammation-based approaches

Microglial and T cell–mediated inflammation in response to Aβ deposition is an early feature of AD progression (see Section 3.2.3), and therefore biomarkers of neuroinflammation may have prognostic value. A group of CSF inflammation-related proteins comprising soluble tumor necrosis factor receptors 1 (sTNFR1) and 2 (sTNFR2), and soluble vascular adhesion molecule 1 identified using principal component analysis, was associated with a halving of risk of progression of MCI participants to AD dementia.[82] The addition of these inflammation markers to other AD biomarkers improved the prediction of cognitive decline over 5 years; those with worst AD biomarkers and lowest levels of sTNFR1 had the fastest decline and progression to AD dementia (Figure S8 in supporting information). In comparison, higher levels of CSF soluble TREM2 (sTREM2), a marker of microglial activation indicative of neuroinflammation, were associated with slower decline in AD dementia.[82] Biomarkers of neuroinflammation may therefore provide complementary information to canonical AD biomarkers.

2.5.4 | Tau-based approaches

Both global and Braak stage tau PET deposition outperformed global Aβ PET deposition (CL) in the prediction of decline in global cognition and episodic memory in ADNI participants across the AD spectrum[83] (Figure S9 in supporting information). More advanced Braak stages were associated with accelerated cognitive decline, independently of global Aβ burden, but global Aβ PET did not remain a significant predictor of future cognitive decline after controlling for tau PET.[83] Given that tau PET scans are expensive and not readily available, several studies investigated whether fluid biomarkers of tau offer similar predictive value. While an increased tau PET temporal meta-ROI was most strongly associated with worse cognition and reduced cortical thickness in ADNI and BioFINDER-2 participants across the AD spectrum, increased CSF and plasma p-tau181 and p-tau217 were most strongly associated with old age, *APOE* ε4 status, and Aβ positivity[84] (Figure S10 in supporting information). Similarly, plasma and CSF p-tau181 were more closely associated with Aβ PET than tau PET (Figure S11 in supporting information).[85] In CU and MCI participants, these biomarkers were associated with Aβ PET whereas tau PET positivity was associated with worse cognition.[86] Tau PET ligands are believed to bind insoluble NFTs, whereas soluble p-tau measures the concentration of tau phosphorylated at specific amino acids that has leaked into the CSF or blood compartments from the extracellular space.[85] Fluid and PET tau biomarkers may therefore reflect different stages in disease progression with fluid tau biomarkers being more related to earlier AD pathology and tau PET to later AD pathology and cognitive symptoms. These differences may be reflected in the discordance between fluid and PET biomarkers.[84,86] When ADNI CU and MCI participants were characterized by plasma p-tau181, CSF p-tau181, and tau PET status, discordance between measures ranged from 6.1% (plasma–/PET+) to 22.4% (plasma+/PET), with ≈ 15% of participants being discordant between CSF and plasma measures.[86] Comparison of tau status determined by CSF p-tau181, tau PET SUVR, and tau PET visual read found that all three modalities were concordant in only 59% of participants across three cohorts including ADNI.[87]

2.5.5 | Neurodegeneration-based approaches

Neurodegeneration, measured using MRI, FDG PET, and fluid biomarkers such as t-tau and NfL, occurs later in disease progression and so

is commonly used to predict cognitive decline or progression. MRI-based classifiers based on modulated GM maps using both a CNN and SVM were tested for generalizability to an external multicenter memory clinic cohort.[88] The classifiers achieved an AUC of 0.756 (SVM) and 0.742 (CNN) for the prediction of MCI progression to AD dementia in the ADNI development cohort, and AUCs from 0.665 to 0.702 in external validation cohorts. The performances in ADNI are consistent with the systematic reviews[76,77] suggesting convergence of prediction methods around AUC 0.75. However, the generally lower performance in the external validation cohorts demonstrates the challenge of generalizing classifiers across diverse cohorts.

A classifier based on the specific organization (small worldness) of GM covariance networks that typify MCI progression was developed in the Amsterdam Dementia Cohort and validated in ADNI.[89] Combined with CSF p-tau181 and hippocampal volume, the classifier identified MCI participants who progressed to AD dementia within 2 years with an AUC of 0.67 in the ADNI validation cohort. Selection of participants for a 2-year randomized controlled trial using the classifier was estimated to nearly halve sample sizes required to detect a 25% slowing in the rate of decline in MMSE and CDR-SB. Consideration of *APOE* ε4 status improved the prediction of the development of AD dementia in presymptomatic patients using structural MRI measures.[90] Disease timelines estimated using a discriminative event-based model differed between *APOE* ε4 carriers and non-carriers in ADNI and were generalizable to the population-based Rotterdam Study cohort. This approach achieved an AUC of 0.81 and 0.88 in *APOE* ε4 non-carriers and carriers, respectively.

Patterns of hypometabolism on FDG PET are also predictive of MCI progression to AD dementia and may outperform MRI-based methods.[76] In an ADNI validation study, an AD progression-related pattern based on hypometabolism derived from FDG PET[91] predicted progression to AD dementia within 3 years with an AUC of 0.796, outperforming the neurodegeneration biomarkers, plasma NfL, and CSF t-tau.[92] However, in a second ADNI validation study,[93] FDG PET and MRI regions selected using radiomics each provided comparable predictive performance. When one modality was added to the other, performance was enhanced only minimally, possibly due to the large overlap of regions selected.

These imaging-based models predict a change in clinical status, but one study suggests that these features may also reflect underlying disease processes. A model derived from structural MRI features that predicted progression from MCI to AD dementia was also associated with early metabolic changes such as insulin resistance and dyslipidemia, and with genetic variants in candidate genes involved in AD-related processes such as Aβ degradation, microglial activation and inflammatory response, synaptic loss, and tau phosphorylation.[94]

Several data-driven approaches have used the ADNI data set to test prediction of decline in cognitive scores rather than diagnostic status. An MRI brain signature region model was associated with baseline and longitudinal episodic memory in ADNI-1, ADNI-2/GO, and the University of California Davis Aging and Diversity Cohort.[95] A data-driven MRI biomarker of dementia risk, the AD-PS score,[96] was trained in ADNI and tested in the Atherosclerosis Risk in Communities (ARIC) data set for its ability to predict incipient cognitive decline in CU participants.[97] The ARIC cohort is more diverse than ADNI, comprised of approximately one third Black American participants, and 12% participants with < 12 years of education. In the full cohort, the AD-PS score outperformed a hypothesis-driven MRI composite ROI score, achieving an AUC of 0.692. However, significantly higher AUCs were achieved in White compared to Black participants, in females, and in *APOE* ε4 carriers, illustrating the need for more comprehensive assessment of prognostic algorithms in diverse populations.

A complementary MRI-based approach used deviation from the norm rather than AD-typical features to detect disease progression in MCI and AD dementia participants from five data sets including ADNI.[98] Normative models were constructed from structural MRI data obtained from UK Biobank CU participants, against which the degree of deviation would indicate disease progression. This measure, most highly significant in medial temporal lobe (MTL) regions in the ventricular system, increased across diagnostic classes and was consistent over differing data sets. This approach achieved AUCs for discriminating between CU and AD dementia of between 0.74 and 0.91, and for discriminating between CU and MCI of between 0.60 and 0.64, demonstrating cross-cohort generalizability.

As MRI scans are often available in the clinic, there has been a recent emphasis on developing medical grade MRI biomarkers to assist in AD dementia diagnosis. Notably, two CNN-based models have been extensively validated for clinical applications.[99,100] The first,[100] developed in a pooled MRI data set of > 85,000 scans including ADNI, and validated in three independent data sets, achieved accuracies of > 90% for diagnosis of AD dementia, and predicted 65% of MCI converters as having AD dementia compared to 20.6% of non-converters. The second[99] not only validated the classification model in multiple cohorts, but in three different MRI vendors, over different protocols, and in low-resolution MRI scans typically commonly available in the clinic. In the ADNI external validation cohort, the classifier achieved a classification accuracy of 0.88 and 0.83 for high- and low-resolution MRI, respectively.

2.5.6 | Combinations and comparisons of AT(N) biomarkers

Combinations of AT(N) biomarkers may better predict disease progression than single biomarkers. However, their predictive ability in part depends on the type of measure used and the cut-point chosen. Fluid and imaging biomarkers are often discordant,[86,101,102] which results in considerable variability in AT(N) classification[101,102] (Figure S12 in supporting information). Tau PET positivity predicted short-term episodic memory better than CSF p-tau181 (or any Aβ biomarker), likely as it becomes positive at a later stage in disease progression than CSF p-tau181.[101,102] In CI participants, tau PET positivity combined with cortical thickness best predicted 12 year longitudinal cognition whereas in CU participants, the best predictors were MRI measures (temporal cortical thickness and hippocampal volume).[102] These studies illustrate that different AT(N) biomarkers cannot be used

interchangeably, and that their ability to predict future decline differs by diagnostic stage.

Biomarkers of neurodegeneration are the least concordant.[101] Although CSF t-tau putatively reflects axonal loss, normal CSF t-tau in the presence of neuronal loss and vice versa is not uncommon.[103] A proteomics study in participants with evidence of neurodegeneration[103] found that those with normal CSF t-tau had increased concentrations of proteins involved in blood–brain barrier (BBB) and blood–CSF barrier dysfunction whereas those with high CSF t-tau had alterations in proteins involved in neuronal plasticity. This biomarker may therefore reflect not only neurodegeneration but leakiness of the brain barriers. Alternative CSF biomarkers reflecting synaptic dysfunction (neuronal pentraxin, 14-3-3 protein ζ/δ) or synaptic plasticity (VGF) enhanced prediction of conversion of late MCI participants to AD dementia over CSF Aβ42 and p-tau181 alone.[104] Moreover, low CSF VGF combined with normal CSF Aβ42 and p-tau181 predicted conversion in early MCI participants, suggesting that it may reflect processes occurring earlier in disease progression than other markers of neurodegeneration.

The choice of cut-point of dichotomized AT(N) biomarkers influences their predictive value. Data-driven methods for the selection of cut-points for Aβ PET, CSF p-tau181, and FDG PET improved the prediction of MCI progression to AD dementia by 30% to 35% over 2 years and ≈ 45% over 4 years over established cut-points.[105] Most cut-points are determined by their ability to discriminate between CU and AD dementia diagnostic groups. However, with the exception of plasma p-tau181,[57] cut-points have not been determined against the gold standard of autopsy to reflect actual neuropathological changes. Using established cut-points for CSF biomarkers, between 50% and 73% of participants across three cohorts who were designated as A+ but T– and were assessed as having an intermediate or high degree of AD neuropathologic change at autopsy[106] (Figure S13 in supporting information). CSF p-tau181 may therefore not accurately reflect tau neuropathology and may instead represent other disease-related processes that occur in response to Aβ deposition. Finally, a dichotomous system for determining biomarker positivity may classify as negative participants with subthreshold but increasing levels of biomarkers who may be on a trajectory of cognitive decline. A lower and a higher cut-point for the CSF Aβ42 ElecSys assay was defined using two-graph receiver operating characteristics of CSF p-tau181/Aβ42 at 90% sensitivity and 90% specificity and delineated three ranges.[107] These cut-points applied to the p-tau181/Aβ40 ratio identified not only fast and slow CU and MCI decliners, but a group with intermediate trajectories of Aβ deposition and cognitive decline that would not have been identified using a dichotomous system (Figure S14 in supporting information). Identification of this intermediate group allows both its exclusion from recruitment to clinical trials, which may reduce required sample sizes, and monitoring of participants who would otherwise have been predicted not to decline under the dichotomous system.

The ability of *APOE* genotype, fluid biomarkers, tau PET, cortical thickness, and baseline cognition to predict decline in MMSE over 2 years in CI participants was tested systematically in BioFINDER 2 and replicated in ADNI.[108] Tau PET outperformed all other biomarkers, and the most parsimonious model combined tau PET with baseline cognition (Figure 3). The prediction of functional decline by AT(N) biomarkers was enhanced by the addition of a baseline cognitive measure.[109] In preclinical AD and MCI participants, the previously validated Discrepancy-Based Evidence for the Loss of Thinking Abilities (DELTA) score improved the ability of Aβ PET SUVR, CSF p-tau181, and hippocampal volume to predict scores on the Functional Activities Questionnaire (FAQ) and CDR-SB.

2.5.7 | Cognitive tests in CU participants

A particular focus in recent ADNI studies of prediction has been identifying and operationalizing for remote use cognitive tests associated with future decline in CU participants. The first signs of subtle cognitive decline can be measured using several tests and scales such as the Everyday Cognition Scale (ECog), Cognitive Change Index (CCI), and the PACC. ECog measures subjective decline in instrumental activities of daily living that map to six cognitive domains, reported by participants themselves or their study partners. A comparison of these methods in ADNI CU participants found that different assessments predicted different measures.[110] Only ECog was associated with future decline in the Montréal Cognitive Assessment (MoCA), whereas only CCI was associated with greater decline in ADAS-Cog. Different assessments also predicted different measures of atrophy, suggesting that measurements of decline are not interchangeable.

Implementation of these tests and assessments online holds great potential for remote screening and monitoring of participants in clinical studies. The online version of self-reported ECog was highly correlated with the in-clinic version, suggesting that remote assessments gather as much information on cognitive and functional abilities as supervised in-person assessments.[111] Another digital cognitive biomarker generated from baseline Rey Auditory Verbal Learning Test (RAVLT) word list memory was developed in the Mayo Clinic, shown to identify CU participants at risk of cognitive decline within 1 to 3 years, and validated in ADNI.[112] Finally, an unsupervised version of the Cogstate Brief Battery taken at home on any device had good concordance with the supervised in-clinic version in CU and MCI ADNI participants, although compliance with test completion diminished over time, suggesting the need for additional long-term support.[113] These studies demonstrate the feasibility and potential of remote monitoring of CU participants and highlight some of the challenges involved in this approach.

2.5.8 | Polygenic risk approaches

ADNI studies have assessed the potential of PRS, calculated from genome-wide association study (GWAS) genotyping data, to predict progression. Ten of twelve publications included in a systematic review of machine learning models to predict lifetime AD risk based on single nucleotide polymorphisms (SNPs) used ADNI data.[75] A PRS including the *APOE* region was more highly associated with an increased risk of progression from MCI to AD dementia than a PRS without *APOE*

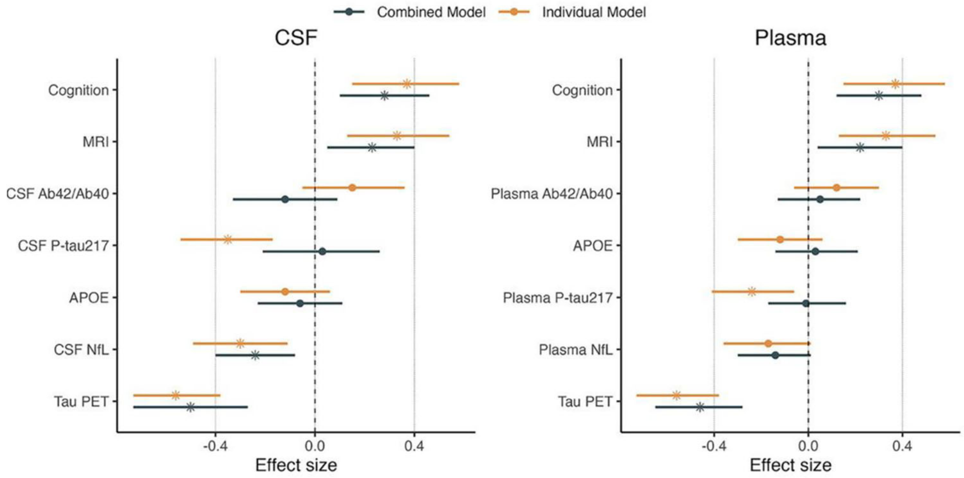

FIGURE 3 Prediction of cognitive decline using biomarkers individually or in combination. Effect sizes for each biomarker in predicting future cognitive decline either alone (orange bars, on top) or in a combined model (black bars, below). Significant biomarkers are represented with a star. The models using CSF biomarkers are shown on the left panel and the models using plasma markers on the right panel. Bars represent 95% confidence intervals. *APOE*, apolipoprotein E; CSF, cerebrospinal fluid; MRI, magnetic resonance imaging; NfL, neurofilament light; PET, positron emission tomography; p-tau, phosphorylated tau. Reproduced under open access from Smith et al.[108]

(hazard ratio 1.468 with *APOE* and 1.293 without *APOE*).[114] Both PRS were better predictors in *APOE* ε4 non-carriers than carriers. A PRS constructed from 40 independent non-*APOE* genome-wide significant SNPs significantly predicted MCI to AD dementia progression in ADNI, which was replicated in the NACC cohort.[115] This PRS was also associated with increased cognitive decline, and longitudinal worsening in a range of other AD biomarkers. Genetic variants reflecting other contributors to disease progression may also be predictive. A PRS comprising genetic variants associated with T2DM explained almost 4% of the variance in MCI to AD dementia conversion, performing only slightly worse than an AD-specific PRS.[116] These studies, together with many others, suggest that genetic loci outside of *APOE* ε4 have small but statistically significant predictive ability.

An issue with the use of PRS is a lack of standardization of PRS calculations including the optimal *P*-value threshold for SNP selection. A comparison of PRS calculations across seven data sets including ADNI reported the best predictive ability was achieved using a *P*-value threshold of $\leq$ 0.1 with the contribution of *APOE* modeled separately.[117]

2.6 | Individualized prediction of future decline

The ability to predict the cognitive trajectory of a patient in routine clinical practice would address the patient's desire to know their personal risk and plan accordingly and may motivate them to modify their lifestyle in beneficial ways. Several studies have used ADNI multimodal data to develop tools to enable this goal. NeuroPM-box, an open access software toolbox, used multifactorial models of disease progression applied to multimodal data to biologically stratify patients.[118] This tool integrated not just imaging and cognitive data, but histopathology and molecular screening (epigenomics, transcriptomics, and proteomics) by taking into account gene-level and brain-level mechanisms of disease progression as well as the potential response for patients to therapeutic intervention (Figure S15 in supporting information). Another framework based on a multifactorial cascade model of disease progression was used to simulate a personalized response to anti-amyloid therapies using either aducanumab or donanemab.[119] The model simulated both short- (78 weeks) and long-term (10 years) individualized responses that took into account potential side effects such as amyloid-related imaging abnormalities (ARIA).

A dementia risk score, the Mild Cognitive Impairment to Dementia Risk (CIDER) score, designed to be used in primary care to predict time to all-cause dementia within 3 years of an MCI diagnosis, was developed in the NACC cohort and validated in two cohorts including ADNI.[120] CIDER was implemented as a nomogram with variables easily determined in clinic: age, sex, education, marital status, hypertension, mood disorder, and either MoCA or MMSE (Figure S16 in supporting information). It predicted dementia risk with a c-index of 0.72 (95% confidence interval: 0.69–0.75) in the ADNI external validation cohort. Knowing where a patient lies on a map of disease progression, in addition to knowing overall dementia risk, could enhance personalized medicine approaches. An AD Course Map of disease progression was developed using hippocampal shape deformations, patterns of progression of hypometabolism and cortical thinning, and neuropsychological assessments.[121] When applied to an individual, the model generates a trajectory that considers an individual's age of disease onset, speed of progression, and genetics and sex.

A multimodal model to predict individual future cognitive decline based on continuous rather than dichotomous biomarker measures was developed in pre-dementia patients and validated in ADNI.[122] The model, comprising age; education; AD signature cortical thickness; hippocampal volume; and CSF t-tau, Aβ42, and Aβ40, more accurately

predicted decline in cognitive scores than a base model of demographics alone. It reduced estimated clinical trial sample sizes over demographics variables alone and is available as a web-based application (https://disease-progression.shinyapps.io/disease_progression/). Similarly, a model predicting 3-year cognitive decline that was developed in BioFINDER 2 and replicated in ADNI comprised baseline tau PET, baseline cognition, CSF NfL, and cortical thickness was operationalized for individual prediction (https://brainapps.shinyapps.io/PredictMMSE).[108] This model reduced clinical trial assessment costs by approximately half compared to no enrichment.

Dementia risk was found to be influenced by sex, *APOE* ε4 status, and age of onset of A*β* positivity in a study of longitudinal biomarkers in ADNI and two additional cohorts.[123] The age of A*β* onset in *APOE* ε4 allele carriers was earlier than in non-carriers and was dose-dependent for the ε4 allele. *APOE* ε4 allele carriers, females, and those with a later age of A*β* onset had a shorter time until the onset of cognitive impairment. Survival curves from the study may form the basis for individualized prediction of time to cognitive impairment based on these factors.

Clinical diagnosis based on neuropsychological tests may not accurately reflect the disease process and therefore prediction of change in diagnostic state may be inherently flawed. To overcome this limitation, a metric, termed the pathology progression rate (PPR), was developed[124] based on a network diffusion model that spatially and temporally describes the spread of AD-related neurodegeneration along brain WM fibers.[125] PPR varied across diagnostic classes and *APOE* ε4 genotype and was associated with global atrophy rate and decline in MMSE. A model comprising a profile of baseline CSF biomarkers identified by hierarchical cluster analysis that represents an intermediate stage of AD neurodegeneration, combined with baseline MRI regional atrophy predicted PPR at the subject level with reasonable accuracy ($r^2 = 0.26$ in linear regression models). The model was less accurate in predicting a global atrophy rate. This approach may offer individualized prediction of biologically based disease progression using MRI scans commonly available in primary care, and CSF biomarkers.

The development of highly sensitive assays for plasma AT(N) biomarkers opens the possibility of their use in individualized prognosis. The ability of plasma A*β*42/40, p-tau (181 or 217), and NfL, alone and in combination, to predict conversion of MCI to AD dementia and longitudinal cognitive decline in individuals over 4 years was compared in BioFINDER and models were validated in ADNI.[126] A model comprising plasma p-tau217 (in BioFINDER) or p-tau181 (in ADNI) and NfL outperformed a base model of demographics and baseline MMSE for the prediction of clinical conversion (AUC of 0.89 compared to 0.74 for the base model in the ADNI validation cohort), achieving comparable accuracies to CSF biomarkers. A*β* biomarkers or *APOE* ε4 genotype did not contribute to prediction. Patient-level models for the conversion to AD dementia and change in MMSE and CDR-SB scores within 4 years were operationalized into an online tool available at predictprogression.com. Similarly, a model developed and validated in the same cohorts to predict individual conversion from MCI to AD dementia and comprising plasma p-tau, *APOE*, memory, and executive function was implemented online at http://predictAD.app.[61] As plasma biomarkers become approved for clinical use, individualized prediction algorithms have great potential, not only in primary care, but for recruitment into clinical trials, substituting for more invasive and/or expensive CSF biomarkers and PET scans. However, it must be emphasized that these studies have been performed in limited cohorts and that further validation in more diverse primary care populations is required.

2.7 | Prediction of AD pathological features

With the recent FDA accelerated approval of anti-amyloid monoclonal antibody therapies, prediction of A*β* status to select those likely to benefit from treatment has become an immediate challenge. As PET scans are expensive and not widely available, and lumbar puncture for CSF is invasive, recent ADNI studies have reported prediction models using modalities accessible in primary care. Such prediction models may also lower costs associated with participant selection for clinical trials.

A model for the prediction of A*β* status in CU participants based on data from the A4 study and optimized for both ADNI and Japanese ADNI consisted of information easily accessed in primary care: demographics, family history, CDR-SB, PACC, and *APOE* ε4 genotype.[127] Other approaches have used additional predictors such as MRI measures and plasma biomarkers.[128,129] Of single AT(N) plasma biomarkers, plasma A*β*42/40 best predicted A*β* PET status in all ADNI participants while plasma p-tau181 had moderate predictive ability in MCI participants.[128] The addition of clinical information (*APOE* ε4 status and education in CU, *APOE* ε4 status and age in MCI participants) and an MRI score to plasma A*β*42/40 improved prediction, but plasma p-tau181 and plasma NfL added little additional predictive ability over clinical information and MRI[128] (Figure S17 in supporting information). Hippocampal morphometry features also predicted A*β* PET burden in CU and MCI participants from ADNI and the Open Access Series of Imaging Studies (OASIS) cohort.[129]

Specific regional tau accumulation is strongly linked to future neurodegeneration and cognitive decline and therefore prediction of tau accumulation may be useful for identifying CU individuals at risk of future decline. A prognostic index able to distinguish between stable CU over 5 years, and declining CU or MCI based on baseline cortical A*β* and MTL volume as continuous variables together with *APOE* ε4 genotype was developed from baseline ADNI data and validated in the Berkeley Aging Cohort Study.[130] The clinically declining group defined by the index had significantly greater baseline global cortical tau and faster accumulation of cortical tau in Braak stages IV and V, and greater decline in PACC than the clinically stable group. This group also accumulated global tau faster than a sample defined by A*β* positivity. The index predicted individual regional variability in future tau accumulation and was estimated to reduce sample size required to detect a 25% decrease in the rate of future tau accumulation by 44%. Use of the prognostic index to define a more stringent threshold for future tau accumulation lowered sample heterogeneity in a simulated trial (Figure 4). Other methods for predicting tau accumulation have been based on the structural connectome;[131] PRS based on SNPs from

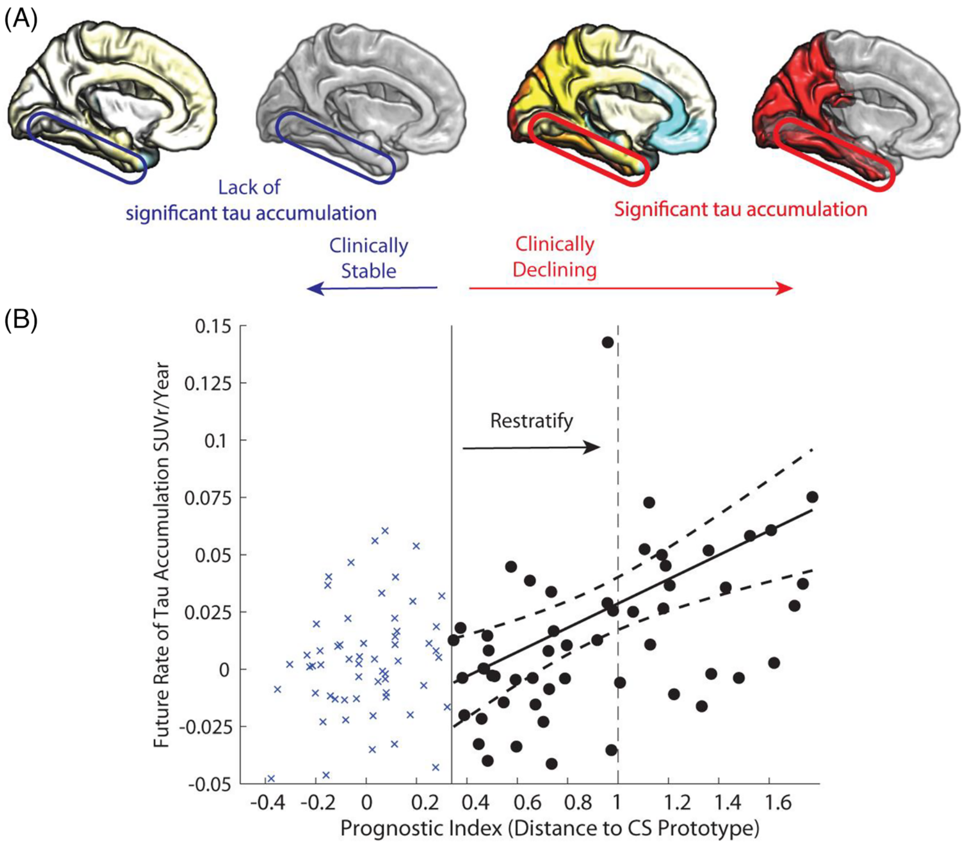

FIGURE 4 Tau prognostic index to predict future tau accumulation. (A), Cortical maps show average rate of tau accumulation for individuals classified as Clinically Stable (CS) versus Clinically Declining (CD). (B), Relationship of the scalar projection with future rate of tau accumulation within the fusiform gyrus (circled in cortical maps are shown in [A]). The solid black vertical line indicates the probabilistic boundary used to perform the binary stratification, blue crosses indicate rate of tau accumulation for the clinically stable group, black circles indicate future rate of tau accumulation for the clinically declining group. Restratification to a more stringent threshold, indicated by the dashed black vertical line, using the prognostic index allows a new sample to be selected with higher future rates of tau accumulation and lower heterogeneity within the sample. SUVR, standardized uptake value ratio. Reproduced under open access from Giorgio et al.[130]

the tau pathway;[132,133] and a combination of demographics, regional cortical thickness, and memory tests.[134]

2.8 | Conclusions

In 2021 and 2022, ADNI studies have contributed to the assessment, development, and implementation of AD therapies and blood biomarkers; described methodological improvements to clinical trials and AD research; and improved AD diagnosis and prediction methods. Of particular note were: (1) the use of ADNI data and methodologies underlying clinical trials that led to the FDA approval of lecanemab; (2) the emergence of plasma p-tau as a promising biomarker of many AD characteristics; (3) the predominance of the ADNI cohort in machine learning AD diagnosis and prediction studies, suggesting a lack of generalizability of these results; (4) the investigation of biomarkers beyond AT(N) such as those reflecting neuroinflammation as predictors of future decline; and (5) the provision of online software tools for individualized prediction of future decline.

3 | ADNI'S CONTRIBUTIONS TO UNDERSTANDING AD DISEASE PROGRESSION

Development of therapies for AD depends on a nuanced understanding of underlying biology and how this affects disease progression. It has become clear in recent years that AD is a complex, multifactorial disease with much heterogeneity in disease course and clinical manifestation. The breadth of ADNI data and availability of samples along with its longitudinal design has facilitated a wide range of approaches to untangle this complexity. In this section, we examine how AD causes cognitive decline and dementia and extensively discuss recent evidence supporting the cascade of events originally proposed in the amyloid hypothesis.[135] We then consider the influence of vascular risk factors on disease trajectory. We examine types and patterns of heterogeneity and factors that contribute to them such as co-pathologies, resilience and sex, and neuropsychiatric symptoms (NPS). By linking lines of evidence from multiple areas of research in a thematic structure, we hope to shed light on how the interaction of these complex factors may account for the observed cognitive

decline in patients and highlight possible novel therapeutic targets and strategies.

3.1 | The relationship of age to AD

In patients in a presymptomatic stage with family history of AD, author-termed "functional brain aging" measured using resting state functional MRI (rs-fMRI) accelerated near the age of parental onset but was not associated with either *APOE* ε4 or Aβ positivity.[136] "Accelerated functional brain aging" was also observed in ADNI MCI and AD participants, suggesting that it was AD specific.[136] A metric predicting brain age from MRI scans was associated with cognitive deficits and was higher in amnestic MCI than CU ADNI participants, and in *APOE* ε4 carriers and those who were A+.[137] Similarly, the brain age gap, the difference between individual brain age, assessed from a temporal pattern of hypometabolism and atrophy, and chronological age, was greater in MCI participants who progressed to AD compared to those who were stable or CU participants.[138] The brain age gap increased at a faster rate in MCI progressors and in females (Figure S18 in supporting information). These studies suggest that some features associated with brain aging such as changes in functional connectivity, glucose metabolism, and brain volume, are accelerated in AD.

Biological age, as opposed to chronological age, can also be assessed using "epigenetic clocks," which aggregate epigenetic changes, such as changes in CpG DNA methylation patterns associated with aging. Of five first-generation epigenetic clocks tested, only the Hannum epigenetic clock was associated with hippocampal volume in ADNI and AIBL.[139] However, ADNI AD participants had extremely high ages on a more sophisticated third-generation epigenetic clock, DunedinPACE. This clock is based on methylation patterns related to the rate of physiological change that characterizes the aging process rather than deviation from chronological age, and so reflects a range of age-related physiological processes from multiple organs.[140] Genetic susceptibility to AD progression and epigenetic age acceleration may be shared.[141] Systemic factors beyond AD pathology and AD genetic risk alleles may contribute to the observed accelerated pathology in AD patients. A range of neurological and systemic biological processes were implicated in a study that identified modules of coexpressed genes associated with accelerated biological aging.[142]

What might these factors be? A comprehensive study of CSF proteomics in CU participants ranging in age from 46 to 89 years from three cohorts including ADNI sheds some light on this question.[143] Of 1149 proteins tested, 911 differed with age; among these 194 were altered in participants older than 60 years; 172, 22, and 352 proteins differed by Aβ status, *APOE* ε4 status, and sex, respectively. Independent of these factors, 252 proteins showed age-related changes, and these were enriched for immune response, signal transduction, and cellular responses to external stimuli. At a genetic and pathway level, there was substantial overlap between healthy aging and AD in a study investigating causal relationships between RNA transcripts and neuroimaging.[144] A relatively small set of genes contributed stably to multimodal imaging and subsequent cognitive decline in healthy aging (Figure S19A in supporting information). In contrast, in AD, the study identified 111 genes and 65 functional pathways with stable causal alterations (Figure S19B), some of which were associated with healthy aging, but had exaggerated effects in different brain areas. Predominant pathways highlighted in AD patients were apoptosis, oxidative stress, and immune/inflammatory response. Leukocyte migration involved in systemic inflammation was associated with accelerated aging on epigenetic clocks.[142] Together, the authors suggested that the changes observed in AD are a result of accelerated aging and enhanced vulnerability of brain substrate to both known risk factors for the disease (Aβ positivity, *APOE* ε4 allele, and female sex), and other physiological processes.

Frailty, defined as "an age-related state of multisystem physiological decline increasing the risk of adverse outcomes,"[145] may modulate the relationship between neuropathology and dementia in AD. Frailty was associated with lower CSF Aβ42 and hippocampal volume, worse glucose metabolism, and greater cortical Aβ binding, and strengthened the association between glucose hypometabolism and dementia. As frailty encompasses a variety of health deficits, these indices may conglomerate diverse mechanisms contributing to dementia such as inflammation and immunosenescence. These associations reflect the complex multifactorial nature of AD.

3.2 | Recent ADNI evidence supporting the amyloid cascade hypothesis

Until recently anti-amyloid therapies, including immunotherapy (monoclonal antibodies and vaccines) and BACE inhibitors, failed to show a significant beneficial effect in clinical trials. However, in the past 2 years, several clinical trials have associated monoclonal antibodies that greatly reduce or eliminate Aβ plaques with significant slowing of cognitive decline.[25,146] These findings support the amyloid hypothesis[135] (Figure S20 in supporting information). Although ADNI is an observational study, numerous analyses of ADNI data have been performed to determine the association of brain Aβ, tau, neurodegeneration, and cognitive decline.

Much of the evidence to support the view that late-onset AD begins with the accumulation of Aβ (subsequently leading to the spread of tau and neurodegeneration) stems from studies of patients with autosomal dominant AD, resulting from a single gene mutation that causes overproduction of Aβ. A recent study explored the degree to which underlying pathophysiology is shared between autosomal dominant AD, in the Dominantly Inherited Alzheimer Network (DIAN) cohort and late onset AD in ADNI.[147] Levels of CSF Aβ42, p-tau181, and t-tau were similar in preclinical and early symptomatic participants from both cohorts when the disease trajectories of both autosomal-dominant and late-onset participants were anchored at the age of symptom onset for comparison. After symptom onset, the rates of change of cognitive impairment and regional atrophy accelerated in both groups, although this acceleration was greater in autosomal-dominant AD participants. The authors conclude that the two forms of AD share pathobiological underpinnings, providing support for the central role of Aβ accumulation in disease progression.

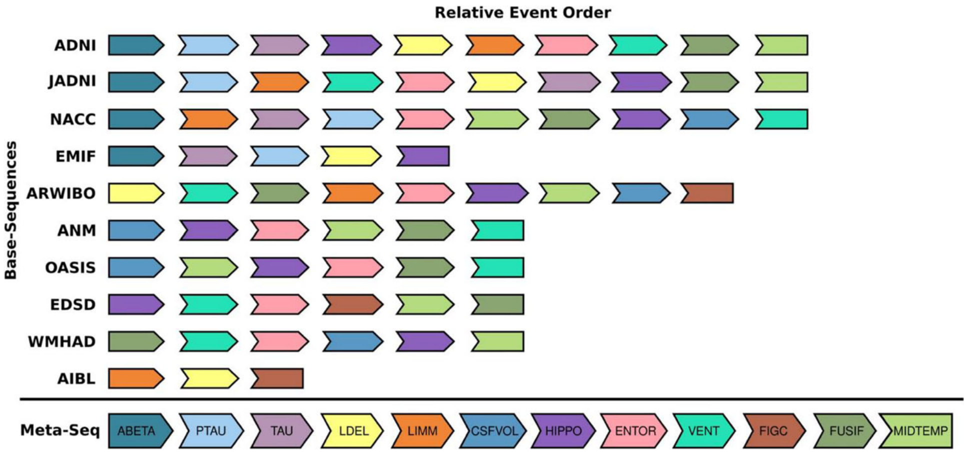

FIGURE 5 Consensus ordering of AD biomarkers from 10 cohorts. All base sequences from the 10 investigated cohorts and the resulting meta-sequence. Due to only partially overlapping lists, the determining factor for an event's position in the meta-sequence was not its absolute position in each base sequence (i.e., rank 1, 2, …, 11), but its relative position to other biomarkers in the same sequence. ABETA, amyloid beta; AD, Alzheimer's disease; AIBL, Australian Imaging, Biomarker, & Lifestyle Flagship Study of Ageing; ANM, AddNeuroMed; ARWIBO, Alzheimer's Disease Repository without Borders; JADNI, Japanese ADNI; EDSD, European DTI Study on Dementia; EMIF, European Medical Information Framework; CSFVOL, accumulated CSF volume in the brain; ENTOR, entorhinal volume; FICG, Figure Copy; FUSIF, fusiform volume; HIPPO, hippocampal volume; LDEL, Logical Memory–Delayed Recall; LIMM, Logical Memory-Immediate Recall; MIDTEMP, middle temporal lobe volume; NACC, National Alzheimer's Coordinating Center; OASIS, Open Access Series of Imaging Studies; PTAU, phosphorylated tau; VENT, ventricular volume; WMHAD, White Matter Hyperintensities in Alzheimer's Disease. Reproduced under open access from Golriz Khatami et al.[150] [Correction added on September 21, 2023, after first online publication: First author name has been corrected for reference 150.]

A neuroimaging study of ADNI CU and MCI participants grouped by AT(N) status (Aβ PET, tau PET, hippocampal volume)[148] provided both cross-sectional and longitudinal evidence for a unidirectional pathway beginning with Aβ deposition, followed by tau deposition and neurodegeneration. Whereas A+T–N– participants had higher tau than A–T–N– controls suggesting sub-threshold tau accumulation, there was no evidence of Aβ accumulation in either A–T–N+ or A–T+N+ participants. Likewise, high baseline Aβ was associated with subsequent tau accumulation, but neither higher baseline tau accumulation nor lower hippocampal volume was associated with subsequent elevated Aβ. Faster decline in PACC was observed only in A+T+N– and A+T+N+ groups, linking tau and neurodegeneration more tightly to cognitive decline. The association of suspected non-Alzheimer's disease pathology (SNAP; T+ and/or N+ in the absence of A+) with cognitive decline likely represents a different neurodegenerative pathway. In a second PET study of ADNI CU participants,[149] overlapping regions of Aβ and tau deposition in AD-typical regions were strongly associated with baseline and longitudinal cognition. Specifically, Aβ to tau but not tau to Aβ, interactions in the MTL were associated with cognitive impairment, supporting Aβ to tau to cognitive impairment directionality. It is important to note that these studies provide strong support for directionality, but do not establish causality of Aβ in initiating AD.

Several studies have investigated the sequence of events in the amyloid cascade using biomarkers. An event-based model fitted to data from 10 cohorts including ADNI[150] largely recapitulated the hypothetical ordering of biomarkers in AD proposed by Jack et al.[151] CSF Aβ42 consistently became abnormal first, followed by p-tau181 or t-tau (Figure 5). The order of these tau biomarkers varied but they were always positioned next to each other, indicating that they are directly linked. There was some variability in the positioning of neurodegeneration biomarkers but there was a consistent sequence of clinical assessments in which memory was impaired first, followed by language and visuospatial ability, and last, executive function (Figure 5).

This ordering of biomarkers is based on established thresholds for positivity, but meaningful changes in biomarkers may occur at sub-threshold levels. When the temporal ordering of CSF and PET biomarkers as well as PACC were aligned relative to time to Aβ positivity assessed by PET,[152] small increases in CSF p-tau181 and MTL tau PET binding were seen 5 to 8 years before the Aβ threshold was reached. Small changes in cognitive dysfunction were also observed in this time frame (Figure S21 in supporting information). CSF Aβ42 reached the positivity threshold before Aβ PET and plateaued approximately two decades later. Aβ accumulation assessed using serial florbetapir PET scans followed a sigmoidal curve that was consistent with hypothesized trajectories[151] and slowed $\approx$ 4 years after reaching the positivity threshold, $\approx$ 10 years before MCI diagnosis.[153] In CU participants below the positivity threshold, the rate of deposition was associated with subsequent tau deposition and decline in memory.

A study that used structural equation pathway modeling to investigate the direct and indirect effects of not only global Aβ PET uptake, regional tau PET uptake, and regional MRI atrophy, but demographics, *APOE* ε4, white matter lesions, and cognition in A+ ADNI CU and CI participants also supported an Aβ–tau–atrophy unidirectional pathway.[154] Importantly, the Aβ–tau–atrophy pathway accounted for only 50% to 56% of the longitudinal variance in cognition, with Aβ, tau, and atrophy individually accounting for 16%, 46% to 47%, and 25% to 29% of longitudinal variance in cognition, respectively. Age,

the *APOE* ε4 allele, and WM lesions all affected cognition but were insufficient to explain the total variance in cognition combined with the Aβ–tau–atrophy axis. These results suggest that other factors such as α-synuclein (α-syn), TAR DNA-binding protein 43 (TDP-43), vascular risk, inflammation, and genetics also contribute to cognitive decline and may help to explain why monoclonal antibodies that remove plaques "only" slow cognitive decline by 25% to 30%. If only 50% of decline is accounted for by the AT(N) pathway, and plaque removal is slowing AT(N)-related decline by 50%, the overall effect would be a 25% slowing of decline.

Together, these studies support a unidirectional sequence of events that occurs on a timeline in which abnormal Aβ rises years before reaching the threshold for positivity, continues on that trajectory for years after reaching the threshold, decelerates a decade before MCI diagnosis, and subsequently plateaus. Phosphorylated tau and t-tau become abnormal followed by neurodegeneration biomarkers and cognitive assessments. The limited success of anti-amyloid therapies to see a clinical benefit in MCI and early AD dementia patients may be partially attributable to timing (at a point at which Aβ deposition is markedly slowing after most of the deposition has already occurred) and target, given that Aβ explains only a small portion of variance in cognition.

In the following sections, we outline recent ADNI studies that have provided evidence for steps in the amyloid cascade, detailing how cellular pathways link Aβ and tau deposition and the cascade of events that lead to neurodegeneration and cognitive decline (Figure S20).

3.2.1 | Abnormal Aβ processing and Aβ accumulation

The earliest (which is not to say causative) step in the amyloid cascade is the disruption of homeostasis of Aβ production. Recent ADNI studies have identified genetic variants involved in this process beyond *APOE*. A large GWAS of plasma Aβ40, Aβ42, and Aβ42/40 levels in > 12,000 nondemented participants from eight studies including ADNI identified variants in amyloid pathway genes *BACE*, *APP*, and *PSEN2*, as well as in *APOE*.[155] Expression of 11 variants of the top 30 non-*APOE* ε4 AD risk alleles in ADNI blood samples, including novel variants in *ADAM10*, *IGHV1-68*, and *SLC24A4/RIN3*, were associated with brain amyloidosis.[156] A large case–control GWAS that included ADNI data as part of the Cohorts for Heart and Aging Research in Genomic Epidemiology (CHARGE) consortium identified six novel genes (*ICA1L*, *DGKQ*, *ICA1*, *DOC2A*, *WDR81*, and *LIME1*), all likely involved in modulation of APP metabolism, which is central to the production of Aβ.[157] Most variants included in a non-*APOE* PRS of Aβ status were involved in biological aspects of the Aβ pathway: regulation of APP catabolism, Aβ formation, or Aβ clearance.[158] This PRS predicted Aβ status even in *APOE* ε4 carriers, further supporting the regulatory role of these non-*APOE* variants.

The micro-RNA miR20b-5p negatively regulated APP expression in human cell lines, and an SNP (rs13897515) upstream of the *MIR20B* gene was associated with CSF Aβ42 and entorhinal cortical thickness.[159] Cleavage of APP by β-secretase (BACE1) may be regulated by a rare variant associated with AD.[160] A rare variant risk association study in two cohorts including ADNI and molecular docking simulation identified rs3120654 (S742Y) in a modulator of BACE1 activity, *FLG*, which encodes an intermediate filament-associated protein.[160] This study provides a molecular-level explanation for the effect of a rare variant associated with AD, via influencing APP processing by BACE1.

The association of the amyloidogenic pathway with downstream events has been documented in recent ADNI studies. A PRS based on variants involved in endocytosis of APP and extracellular aggregation of Aβ (*BIN1*, *CD2AP*, *EPHA1*, *PICALM*, *SORL1*, and *CD33*) correlated with baseline CSF t-tau.[161] Spatial patterns of hypometabolism measured by FDG PET were associated with patterns of expression of *BACE2* and *NDUFS4*, a mitochondrial protein that binds oligomeric Aβ and has been implicated in cognitive deficits.[162]

Finally, the minor allele (C) of rs10751647 in *IFITM3* was associated with not only decreased brain Aβ deposition, but also lower CSF p-tau181, greater entorhinal cortical thickness, and slower cognitive decline and progression to AD dementia.[163] *IFITM3* encodes interferon-induced transmembrane protein 3, which upregulates γ-secretase in response to viral infection, suggesting that an innate immune response to microbial infection may increase Aβ deposition.

3.2.2 | Impaired clearance of extracellular Aβ

The removal of extracellular Aβ from the perivascular space into CSF via the glymphatic system has been proposed to be disrupted in AD, leading to accumulation of extracellular Aβ and deposition into plaques. In the glymphatic system, polarized distribution of aquaporin 4 channels in astrocytic end feet promotes the rapid movement of CSF to the brain interstitial space. There it mixes with interstitial fluid and waste solutes such as Aβ before being drained out of the brain via the perivenous influx pathway[164] (Figure S22 in supporting information). Two SNPs in *AQP4*, the gene encoding aquaporin 4, were associated with in vivo brain Aβ independently of the *APOE* ε4 allele.[165] MRI measures reflecting the glymphatic system, such as the volume of and diffusivity into the perivascular space, were worst in participants with AD dementia, and intermediate in participants with MCI.[164] This indicated an enlargement of the perivascular space with disease progression and a concomitant decrease in diffusivity of CSF into that space. Changes in these measures in MCI and AD participants were significantly associated with CSF Aβ42, FDG PET, and cognition.[164] Enlargement of the perivascular space in the centrum semiovale was associated with WMH volume,[166] glucose hypometabolism in A+ participants,[167] brain tau,[168] diagnostic conversion and longitudinal cognitive decline,[169] and sTREM2.[166] sTREM2, in turn, mediated the association between enlarged perivascular space in the centrum semiovale and CSF p-tau181.[166] There are several possible explanations for these findings. These findings may implicate dysfunction of the glymphatic system, by sleep disturbances or small vessel disease (indicated by WMH volume), as a key causal event in pathogenesis. In this scenario, disruption

of Aβ clearance contributes to its accumulation, which may trigger a microglial inflammatory response that results in further enlargement of the perivascular space in a feedback loop via tau. A second possibility is that these changes are secondary to the neurodegeneration that occurs in AD and so may not reflect a cause–effect relationship.

As the glymphatic system operates primarily during sleep, several ADNI studies have investigated the role of sleep, derived from self-report assessments, in the clearance of Aβ. An rs-fMRI study that detected CSF movements indicative of glymphatic clearance linked this signal with cortical Aβ binding, diagnosis, cognition, symptom severity, and the *APOE* ε4 allele.[170] Disturbances to sleep appear to be associated with increased AD risk (i.e., a cause-and-effect relationship), and may offer a treatment target. A+ ADNI participants who reported insomnia had faster cognitive decline than those without insomnia.[171] Baseline informant-reported sleep disturbances interacted with the *APOE* ε4 allele alone, and CSF Aβ42 and p-tau181/Aβ42 together, to enhance regional atrophy rates.[172] Self-reported obstructive sleep apnea (OSA) was associated with a shorter time of progression from CU to MCI, and from MCI to AD.[173] The risk of progression was increased by 2- to 3-fold in A+ participants with OSA, and by 3- to 5-fold in (TN)+ participants with OSA, compared to participants with no OSA.[173]

These results may suggest a causal relationship in which sleep disturbances perturb the glymphatic system, resulting in a heightened risk of disease progression mediated by a synergistic interaction with Aβ and tau. Alternatively, it is possible that the neurodegeneration caused by AD pathology causes sleep disturbances or that both mechanisms occur.

Aβ metabolism in blood has been proposed to affect Aβ clearance from the brain.[174] A cis- expression quantitative trait loci (eQTL) analysis of 29 AD-associated SNPs identified elevated expression of *APH1B*, encoding a subunit of γ-secretase, in blood. This was associated with global Aβ burden, entorhinal cortex (EC) thickness, and MCI to AD progression, and the authors suggest that *APH1B* may represent a novel therapeutic target.[174]

3.2.3 | Immune response and inflammation

Chronic low-grade inflammation is a hallmark feature of AD, and the importance of immune response in AD pathogenesis has been increasingly recognized. An immune pathway–specific PRS constructed from top AD risk loci was associated with increased longitudinal Aβ deposition, regional atrophy and hypometabolism, and worse cognition.[161] In response to Aβ deposition, microglia and astrocytes are activated, causing a neuroinflammatory response that can be exacerbated by peripheral inflammation. This vital step in the cascade of disease progression links Aβ deposition with downstream events eventually leading to tau deposition. Recent ADNI studies have provided evidence for the importance of this step and helped identify potential normal therapeutic targets.

Several studies have investigated associations of a marker of disease-activated microglia, sTREM2 in CSF with AD biomarkers. In ADNI participants across the AD spectrum, elevated sTREM2 was associated with old age and CSF AD biomarkers (Aβ42, p-Tau181, and t-tau) as well as cytokines related to inflammation (chitinase-3-like protein 1 [YKL-40], progranulin, interleukin [IL]-10, transforming growth factor [TGF]-β1, tumor necrosis factor alpha [TNF-α], C3, factor H) and neurodegeneration (NfL, synaptosomal-associated protein 25, neurogranin, growth-associated protein 43) but not with brain metabolism, MRI volumes, or cognition.[175] In contrast, in another study, elevated CSF sTREM2 predicted cognitive decline in A+ but not A– MCI and AD ADNI participants with an effect size comparable to that of CSF p-tau181 but less than MRI volumes.[176]

The disparity between these results may reflect a stage-dependent effect of microglial activation. GWAS have identified *TREM2* as a protective gene in AD (reviewed in Veitch et al.[8,9] and Weiner et al.[13]) that promotes anti-inflammatory cytokine expression and reduces pro-inflammatory cytokine release, activating microglia to surround and endocytose Aβ plaques. However, this response may eventually become detrimental later in disease progression, causing neuronal damage and facilitating disease progression. Higher levels of baseline CSF sTREM2 were associated with lower regional atrophy and WM damage in A+T+ MCI participants but with increased regional WM damage in A+T+ AD participants.[177] The action of CSF sTREM2 at different disease stages may be further modulated by interaction with CSF tumor necrosis receptor 2.[178]

Microglia may also be activated by other upstream factors. Mediation analysis of CSF IL-3, a marker of astrocytic activation, sTREM2, and canonical AD biomarkers indicated that IL-3 modulated the levels of sTREM2 in response to Aβ deposition, and was associated with subsequent tau pathology and cognitive decline.[179] The authors suggest that cross-talk between astrocytes and microglia is an important link between Aβ and tau deposition in disease progression (Figure S23 in supporting information). Changes in iron homeostasis, critical for central nervous system function, may also affect CSF sTREM2. High levels of CSF ferritin, a biomarker of brain iron accumulation, were significantly associated with accelerated accumulation of CSF sTREM2 in participants stratified by AT(N) across the AD continuum as well as controls.[180] The authors suggested that this may indicate a role for iron-induced neuroinflammation. CSF ferritin levels increased across the AD continuum,[181] and higher CSF ferritin was associated with worse cognition,[181,182] worse CSF p-tau181,[181,182] and with other inflammatory proteins[181] but not Aβ.[181,182] The association between CSF ferritin and p-tau181 was mediated by *APOE*.[182] The contribution of iron metabolism to AD disease progression appears to be a complex process involving microglial activation, p-tau, and *APOE*.

Other markers of neuroinflammation have been associated with AD pathogenesis in recent studies. CSF IL-12p40, TNF-α, and IL-9, biomarkers involved in the nuclear factor kappa-light-chain-enhancer of activated B cells (NFκB) pathway that transcriptionally regulates many genes involved in immune response, were associated with clinical progression to MCI or AD.[183] CSF progranulin acted in a protective manner. Higher levels of proganulin were associated with lower Aβ burden in T+ and/or N+ participants only via interaction with neuroinflammatory markers (sTNFR1, sTNFR2, TGF-β1, intercellular

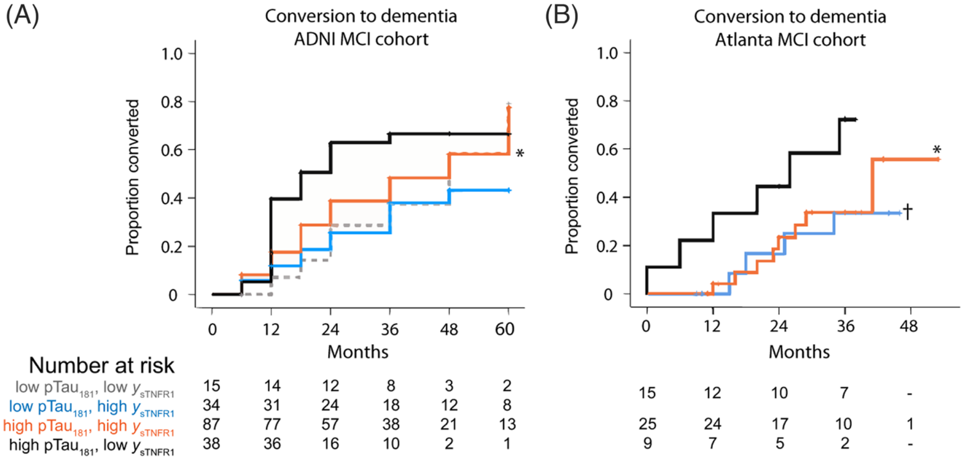

FIGURE 6 Conversion to dementia diagnosis by levels of CSF p-tau181 and sTNFR1. (A) ADNI. (B) Atlanta replication cohort. *Lower conversion compared to those with p-tau$_{181}$ ≥ 24.1 pg/mL but low yf_{sTNFR1} ($P = 0.049$ in ADNI and $P = 0.038$ in the Atlanta cohort). †Two subgroups with p-tau181 < 24.1 were combined due to small numbers, $P = 0.068$ versus high p-tau181 and low y_{sTNFR1}. ADNI, Alzheimer's Disease Neuroimaging Initiative; CSF, cerebrospinal fluid; MCI, mild cognitive impairment; p-tau, phosphorylated tau; sTNFR1, soluble tumor necrosis factor 1. Reproduced under open access from Hu et al.[82]

adhesion molecule 1, and vascular cell adhesion molecule 1).[184] This complex interplay of inflammatory proteins that are regulated by both pro- and anti-inflammatory processes makes selection of a single AD-relevant CSF biomarker of inflammation challenging. A study of 15 CSF inflammatory proteins involved in microglial- and T cell–mediated inflammation identified sTNFR1 as a prognostic biomarker in MCI that added predictive ability above and beyond CSF p-tau181.[82] MCI patients with the lowest levels of this biomarker in addition to the highest levels of CSF p-Tau181 had the worst prognosis over 5 years (Figure 6). Higher levels of sTNFR1 therefore appeared protective, and this biomarker may provide complementary information to core AD CSF biomarkers.

The detrimental effects of neuroinflammation appear to be exacerbated by various other factors such as sex, *APOE* status, and cerebrovascular disease. Female *APOE* ε4 allele carriers with high levels of CSF IL-12p40, TNF-α, and IL-9 had the greatest risk of progression,[183] and higher levels of CSF sTNFR2 were associated with worse cognition, mediated by CSF p-tau181, in women but not men.[185] Higher levels of eight proinflammatory CSF biomarkers combined with lower levels of cerebrovascular disease, measured by pulse pressure, were associated with higher levels of CSF Aβ42 in CU ADNI participants.[186] However, in MCI participants this combination was associated with higher levels of CSF p-tau181 and t-tau but not Aβ42.[186] These results again support the hypothesis of an initial protective function of neuroinflammation followed by a shift to detrimental effects at later disease stages. The contributions of vascular disease to AD will be further described in Section 3.3; sex differences in Section 3.7.

Peripheral inflammation is frequently associated with cardiovascular disease and T2DM, which are risk factors for dementia. Recent ADNI papers have suggested a role for peripheral inflammation in exacerbating disease progression. Pro-inflammatory cytokines, released by neuroinflammation in response to Aβ deposition, can recruit peripheral immune cells which can take advantage of changes in the permeability of the BBB and infiltrate the brain.[187] Genes associated with immune cell infiltration were identified from comparison of differentially expressed BBB- and immune-related genes in blood from ADNI participants with AD dementia and CU controls.[187] Five genes (*TNFRSF13C*, *CXCL3*, *CXCL12*, *CCL4L1*, and *CCL1*) overlapped between the two sets and were associated with CSF AD biomarkers.[187] Expression of a pro-inflammatory receptor (P2X purinoceptor 7) and two integrins (CD11b and CD11c) involved in phagocytosis on leukocytes was lower in A+ CU ADNI participants and associated with higher Aβ burden, more severe atrophy, and worse cognition.[188]

In a UK Biobank study with validation in ADNI, two SNPs in two AD risk alleles involved in neuroinflammation, *CLU* and *CD33*, were associated with faster MCI to AD progression in the presence of C-reactive protein (CRP), a marker of peripheral inflammation[189] (Figure S24 in supporting information). Elevated CRP was found only in *APOE* ε4 homozygotes and was associated with increased CSF p-tau181 and t-tau, and worse global cognition.[190] These results implicate dysfunction of the peripheral immune system in AD progression, which may exacerbate genetic vulnerability for AD.

A number of studies have associated immune-related variants with AD. Gene- and pathway-level mapping of rare eQTL in ADNI blood samples and brain tissue from the Religious Orders Study/Memory and Aging Project (ROSMAP) cohort identified rare and low-frequency variants involved in inflammation mediated by cytokines and chemokine signaling.[191] These included five genes previously linked to AD (*ALOX5AP*, *CXCR2*, *FPR2*, *GRB2*, *IFNAR1*). Rare variants in *NLRC3* were associated with neuroinflammation (CSF YKL-40).[192] These may act via counteracting the inhibitory action of NLRC3 on the NFκB and NLRP3 inflammasome signaling pathways.[192] Further evidence for the central role of these signaling pathways came from a large case–control GWAS that included ADNI data as part of the CHARGE consortium.[157] Of the 75 risk loci identified, most were expressed in microglia. Pathway enrichment analysis identified

immune-associated gene sets involved in the regulation of the TNF-α–mediated signaling pathway. Prioritized genes included *SHARPIN*, *RBCK1*, and *OTULIN*, which implicated the involvement of the linear ubiquitin chain assembly complex, essential for the activation of the NLRP3 inflammasome and involved in TNF-α–mediated signaling. A PRS constructed from these variants and others involved in APP metabolism that excluded *APOE* was associated with 1.9-fold increased risk of future conversion from MCI to AD.[157]

3.2.4 | Metabolic disturbances

Metabolic disturbances are an early feature of AD, resulting from the combined influence of AD neuropathology, genetic variations, microbiota, immune response, and lifestyle and diet.[193] Subtle changes in metabolites, predominantly amino acids and lipids, were observed in presymptomatic AD, preceding more pronounced changes later in disease progression in combined Knight ADRC and DIAN cohorts with replication in ADNI and ROSMAP.[194] A systems biology approach using data from the AD Metabolomics Consortium including ADNI data investigated metabolite coexpression networks and their genetic regulators in relation to AD progression.[193] Branched chain amino acids and short chain acylcarnitines decreased with disease progression with a concomitant increase in medium and long chain acylcarnitines over time correlating with NFTs in the frontal cortex. This disturbance of homeostasis was found to be regulated by *ABCA1* and *CPT1A*, and by adiponectin, a regulator of ABCA1. Expression of *ABCA1* and levels of adiponectin increased across diagnostic groups (Figure S25 in supporting information). A second study linked serum metabolites to global Aβ burden in addition to cognitive performance and MCI progression to AD dementia.[195] Seven phosphatidylcholines (PCs) were associated with increased cortical Aβ burden and an acylcarnitine (C3) and kynurenine were associated with decreased cortical Aβ burden. All differed in patterns of association with regional Aβ burden (Figure S26 in supporting information). Of these metabolites, C3, kynurenine, and several PCs were associated with worse memory and executive function, and one PC was associated with MCI conversion. A lipid metabolism PRS constructed from known AD risk loci including *CLU*, *SORL1*, and *ABCA7* was also associated with increased cortical Aβ burden and with neurodegeneration.[161] These studies support the role of a wide range of Aβ-dependent lipid perturbations in influencing neurodegeneration, cognitive decline, and diagnostic progression.

Perturbations of PC metabolism may underlie the vulnerability of cholinergic neurons of the basal forebrain as one of the first sites of neurodegeneration in response to Aβ and tau deposition. Cluster analysis of serum lipids identified unsaturated PCs associated with significantly higher GM atrophy in the basal forebrain nucleus basalis of Meynert in participants with AD pathology compared to those without.[196] Cholinergic neurons have high metabolic demands for cell membrane maintenance. These results support the hypothesis that AD pathology contributes to their heightened vulnerability via abnormal PC metabolism.

Beyond PCs and acylcarnitines, other lipid classes have also been implicated in AD. Levels of glycerophospholipids, cholesterol esters, and complex sphingolipids, as well as PCs and acylcarnitines, were altered in MCI and AD participants compared to CU, and associated with CSF p-tau181/Aβ42.[197] Clustering analysis identified two lipodomic endophenotypes with hazard ratios for MCI clinical progression greater than that for the *APOE* ε4 allele (1.97 and 1.99 compared to 1.48).[197] Metabolism of sphingomyelin, a type of sphingolipid found in the myelin sheath and in lipid rafts in microglia, astrocytes, and neurons, is dysregulated in early AD.[198] An integrated multi-omics study focused on the sphingomyelin pathway, in which increased activity of sphingomyelin phosphodiesterase in response to Aβ deposition results in cleavage of sphingomyelin to form neurotoxic ceramides.[198] The study found that genes in this pathway were differentially expressed in AD patients compared to controls and used multimodal neuroimaging to identify variants (*SPTLC3* and *SGMS1*) associated with AD pathogenesis. A modulator of sphingosine-1-phosphate, an inhibitor of sphingomyelin phosphodiesterase, was identified as a potential AD drug target that may preserve synaptic function.

These metabolic changes reflect different components of the cascade of disease progression. Membrane restructuring that leads to brain Aβ accumulation is reflected in altered lipid metabolism[195] and these changes can trigger downstream responses such as apoptosis, neuroinflammation, and APP processing within lipid rafts.[195] Thus, changes in lipid metabolism may mediate the relationship between Aβ deposition and neurodegeneration. Activation of the kynurenine pathway occurs in response to neuroinflammation, and increased levels of acylcarnitines result from elevated oxidation of fatty acids and amino acids, reflecting perturbation of mitochondrial function.[195]

3.2.5 | Mitochondrial abnormalities

Mitochondrial dysfunction, resulting in a loss of energy production (visible by FDG PET), synapses, and neuronal vitality and longevity, plays a key role in AD. The mitochondrial cascade hypothesis posits that declining mitochondrial function resulting from age and genetic and environmental factors is an upstream event that results in Aβ and tau pathology.[199] It is also possible that declining mitochondrial function is a byproduct of the amyloid cascade. Recent ADNI studies investigated the involvement of mitochondrial dysfunction in disease progression and identified novel therapeutic targets.

Several studies have investigated associations between genes involved in mitochondrial function and AD. Mitochondria have a very limited number of genes, and most genes involved in mitochondrial function are encoded in the nucleus. Mitochondrial contributions to pathogenesis are therefore likely attributable to nuclear genes. An AD PRS comprising nuclear-encoded mitochondrial genes, and three pathway PRS from genes involved in mitochondrial pathways, were significantly associated with AD with odds ratios ranging from 1.22 to 2.01.[200] Significant mitochondrial pathways were mitochondrial transport, hallmark oxidative phosphorylation, and response to oxidative stress. A GWAS of mitochondrion-associated genes and

subsequent epistasis analysis identified six modules of nuclear genes associated with AD pathogenesis that interacted with mitochondrial genes.[201] Top hub genes identified through network analysis were involved in synaptic function, signaling pathways, and neurodegenerative pathways, demonstrating how mitochondria-related biological processes may contribute to AD pathogenesis beyond reduced energy metabolism. The only gene identified from all investigative approaches was *APP*, consistent with its central role.

Spatial patterns of tau pathology were associated with genes involved in metabolism, as well as oxidative phosphorylation, mitochondrial respiration, and electron transport, implicating mitochondrial dysfunction in addition to metabolic disturbances in tau deposition.[162]

3.2.6 | Tau deposition

Tau deposition is a critical step in AD pathogenesis. Brain tauopathy was associated with 10 of the 20 top AD risk genes (*ABCA7*, *BIN1*, *CASS4*, *CLU*, *CR1*, *EPHA1*, *NME8*, *SORL1*, *DSG2*, and *ZCWPW1*)[202] and with expression of 7 of the 30 top non-*APOE* risk alleles.[156] Hyperphosphorylation of tau in microtubules occurs when signaling pathways are altered for a variety of reasons including the Aβ-induced, microglia-mediated inflammatory response. This causes tau deposition into NFTs with concomitant cytoskeletal abnormalities that result in synaptic dysfunction preceding neuronal death.[202] AD risk genes associated with tau deposition have a variety of cellular functions involved in this process such as tau phosphorylation (*BIN1*), lipid transport (*ABCA7*), cellular adhesion signaling (*CASS4*), and immune function (*CR1*).[202]

Recent ADNI studies have provided further support for the critical function of tau deposition in the chain of events ultimately resulting in cognitive impairment. The *BIN1* SNP rs744373 risk allele was associated with faster tau PET accumulation in ADNI and AIBL participants.[203] Carriers of the risk allele had greater global tau PET and greater cognitive decline than noncarriers. Tau accumulation was even greater in A+ risk allele carriers and mediated cognitive decline (Figure S27 in supporting information). This is in agreement with the modeling study that estimated tau to account for almost half of the variance in cognitive decline[154] and with a meta-analysis of 24 studies including three ADNI studies that estimated an overall weighted effect size of –0.46 (95% confidence interval [–0.73; –0.20], $P < 0.001$) for the association of tau protein biomarkers with episodic memory.[204] Additionally, three novel variants (minor alleles in *ZBTB20*, *EYA4*, and *VNN2*) identified in a GWAS of brain tauopathy were associated with cognitive decline mediated by tau PET.[205] ZBTB20 is a transcription factor expressed in dendritic cells previously identified in other neurological disorders, and expression of *VNN2* is regulated by the kinase, EYA4, which may be involved in tau hyperphosphorylation.[205]

A haplotype of *KLOTHO* (*KL*), encoding a transmembrane protein associated with brain health, was shown to be protective in combination with the *APOE* ε4 allele.[206,207] KL*VS heterozygosity (carrying only one copy of the functional haplotype encoding F352V and C370S) lowered risk of Aβ positivity only in *APOE* ε4 carriers.[206] At pathological levels of global Aβ PET, KL*VS heterozygosity was associated with lower increases in regional tau PET, especially in *APOE* ε4 allele carriers, which mediated better memory[207] (Figure S28 in supporting information). These results suggest that KL*VS heterozygosity counteracts the detrimental effects of the *APOE* ε4 allele on memory by protecting against Aβ-related increases in tau deposition, contributing to resilience.

3.2.7 | Axonal and synaptic dysfunction

Axonal and synaptic dysfunction prior to neuronal death is a crucial step in disease progression that has been difficult to measure, largely because well-established biomarkers to track this process are lacking.[71] This step represents the first common point at which different contributing factors to AD converge, not only hyperphosphorylated tau, but inflammation, oxidative stress, genetic contributions, altered CA^{2+} homeostasis, and more.[71] A compensatory response involving microglia and astrocytes, which engulfs and clears accumulated synaptic debris, is eventually overcome resulting in a net loss of synapses.[208] The development of CSF and, in some cases, plasma biomarkers of axonal and synaptic damage has allowed identification of additional genetic risk factors, better tracking of disease progression, and assessment of whether putative medications are engaging their target. ADNI's collection of CSF and blood samples has facilitated many of these analyses.

An exploratory analysis that linked densities of 15 neurotransmitter receptors derived from brain donors at the University of Düsseldorf with ADNI neuroimaging data (tau, Aβ, and FDG PET, and structural, functional, and arterial spin labeling MRI) suggested that much of the variability in cognitive decline can be attributed to receptor differences.[209] Differences in the interactions of three neurotransmitter receptors with neuroimaging modalities explained up to 70% of population variability in cognitive decline, primarily in executive function. Glutamatergic receptor interaction changes affected neuronal activity and tau accumulation, and GABAergic receptor changes additionally affected Aβ accumulation. Cholinergic receptor interaction changes affected tau accumulation. Dysfunction of these neurotransmitters may be involved in AD pathological neurodegeneration.

A marker of presynaptic dysfunction, growth associated protein 43 (GAP43), reflects the importance of disruption of the synapses in AD disease progression. GAP43 was measured in ADNI CSF samples by the clinical neurochemistry lab at the Sahlgrenska University Hospital, Sweden, and subsequent data were analyzed by many groups. This biomarker increased over diagnostic classes[210] and was strongly associated with CSF p-tau181 levels,[210–212] but less so with Aβ PET positivity[212] and not at all with CSF Aβ42.[210,211] Higher baseline levels or more rapidly increasing levels of CSF GAP43 were associated with greater hypometabolism,[210–212] atrophy,[210–212] cognitive decline,[210–212] and MCI to AD dementia progression.[211,212] This discrepancy between the association of CSF GAP43 with Aβ may be because CSF Aβ42 positivity occurs earlier than Aβ PET positivity, positioning Aβ PET positivity closer to synaptic dysfunction. Aβ

PET positivity worsened cognitive outcomes in GAP43-positive and -negative participants.[212] These studies suggest a pathway in which synaptic dysfunction driven by increased tau pathology (and to a lesser extent, Aβ deposition) results in neurodegeneration and cognitive decline.

Not all synaptic dysfunction resulting in cognitive decline is attributable to AD pathology. An ADNI study of CU and MCI participants investigated associations of five putative CSF biomarkers of synaptic damage and loss (chromogranin A, fatty acid binding protein, neuronal pentraxin 2 [NPTX2], secretogranin, and neurosecretory protein VGF) with AD pathology, atrophy, and cognitive decline.[213] Of these proteins, NPTX2 had the greatest within-subject declines over 3 years and correlated most strongly with cognitive decline. Although CSF NPTX2 declined more in participants with a positive baseline CSF p-tau181/Aβ42 ratio than in those with a negative ratio, the greatest decline was seen in participants with stable or slightly declining CSF p-tau181. Therefore, CSF NPTX2, unlike CSF GAP43, may not be associated with accelerated cognitive decline that occurs because of AD pathology. This biomarker, which modulates postsynaptic AMPA-type glutamate receptors, may therefore reflect other neurodegenerative processes and represent a novel therapeutic target for intervention.

Like all CSF biomarkers, these require invasive lumbar puncture and blood biomarkers are therefore preferable. However, peripheral levels of brain proteins often do not reflect levels in the brain due to the difficulty of diffusion through the BBB. The use of brain-derived EVs that can be easily measured using techniques such as flow cytometry circumvents this problem. A study using ADNI as a replication cohort examined levels of N-methyl D-aspartate receptor 2A (NMDAR2A) in brain-derived EVs.[71] NMDAR2A is a glutamate receptor that is the therapeutic target of memantine and so acts as a marker of synaptic function. Levels of NMDAR2A were lower in AD compared to CU participants and were able to discriminate between these two groups with an AUC of 0.81 in the ADNI validation cohort.

A GWAS of the CSF biomarkers NfL (axonal damage), neurogranin (synaptic degeneration), and YLK-40 (astroglial activation) used ADNI as a validation cohort.[214] This study identified rs1548884 in *TMEM106B*, an established risk gene for frontotemporal lobe dementia (FTLD), as associated with CSF NfL. A further 124 SNPs were in strong linkage disequilibrium with this variant, 80 of which reached a genome-wide significance, pointing to the importance of this gene. In contrast, established AD risk genes including *APOE* were not associated with CSF NfL. ADNI whole genome sequencing data confirmed two novel rare variants associated with AD.[215] The first locus was in *DLG2*, encoding a synaptic scaffold protein thought to be downregulated early in AD, and the second was in *DTNB*, encoding a neuronal-associated protein possibly involved in postsynaptic function.

3.2.8 | Trans-synaptic spread of tau

In AD, tau pathology usually spreads through the brain in a set pattern reflected in Braak staging.[216] From an initial hub in the EC, tau pathology spreads from the MTL via synaptic connections to the neocortex, where it becomes closely linked to neurodegeneration.[217] Recent ADNI studies of structural and functional connectivity based on MRI tractography, cerebral blood flow (CBF), and rs-fMRI combined with Aβ, tau, and FDG PET have provided insights into the trans-synaptic spread of tau across these networks.

Tau PET data suggest that tau exhibited neuron to neuron transmission along topological connections of the structural connectome of WM from hub to non-hub regions, rather than along spatially adjacent connections.[218] This pattern of spread may be associated with alterations in properties of WM. The pattern of association between WM microstructural alterations characterized by greater diffusivity and lower axonal packing density and tau PET resembled Braak stages.[219] The degree of myelination in WM tracts also correlated with tau PET spread, with lower levels of myelin sheath being associated with a greater susceptibility of WM tracts to tau accumulation.[220] Demyelination increased with disease severity and correlated with worse CSF Aβ42, plasma NfL, and accelerated cognitive decline.[221]

The trans-synaptic spread of tau is also associated with brain functional networks. Tau deposition was higher within functional connectivity networks.[222] Clusters of tau deposition within functional connectivity networks correlating with Braak stages were associated not only with later Braak stages, but with earlier Braak stages, suggesting that tau spread may be bidirectional.[222] Brain functional networks become more diffuse and desegregated with aging.[223] In A+ ADNI participants with a given level of MTL tau in the EC, higher functional network segregation was associated with slower rate of tau accumulation in cortical regions corresponding to Braak stages III and IV[223] (Figure S29 in supporting information). This relationship was also found in participants with different initial tau epicenters that resulted in patterns of spread divergent from Braak staging.[223] A mediation analysis study in non-demented participants from the Baltimore Longitudinal Study of Aging (BLSA) and ADNI investigated the extent to which Aβ versus tau deposition in the MTL drove tau propagation to the neocortex.[217] Greater MTL tau in the EC was associated with greater neocortical tau in the inferior temporal gyrus (ITG), and this association was stronger in A+ participants.[217] Greater Aβ in the EC was associated with greater ITG tau both directly and indirectly via EC tau (Figure 7). Neurodegeneration markers were directly affected by ITG tau, but only indirectly affected by EC Aβ. This synergistic interaction between MTL Aβ and tau to influence tau neocortical propagation and subsequent neurodegeneration suggests that removal of either pathology would be insufficient to prevent neurodegeneration.

3.2.9 | Genomic basis of neurodegeneration and cognitive decline

As AD is a complex disease, neurodegeneration results from the convergence of multiple pathways. The expression of eight variants in top AD risk alleles in blood was associated with neurodegeneration.[156] Three of these were also associated with brain amyloidosis and one was also associated with tauopathy, implying that a range of common and distinct mechanisms underlie neurodegeneration. ADNI genomics and

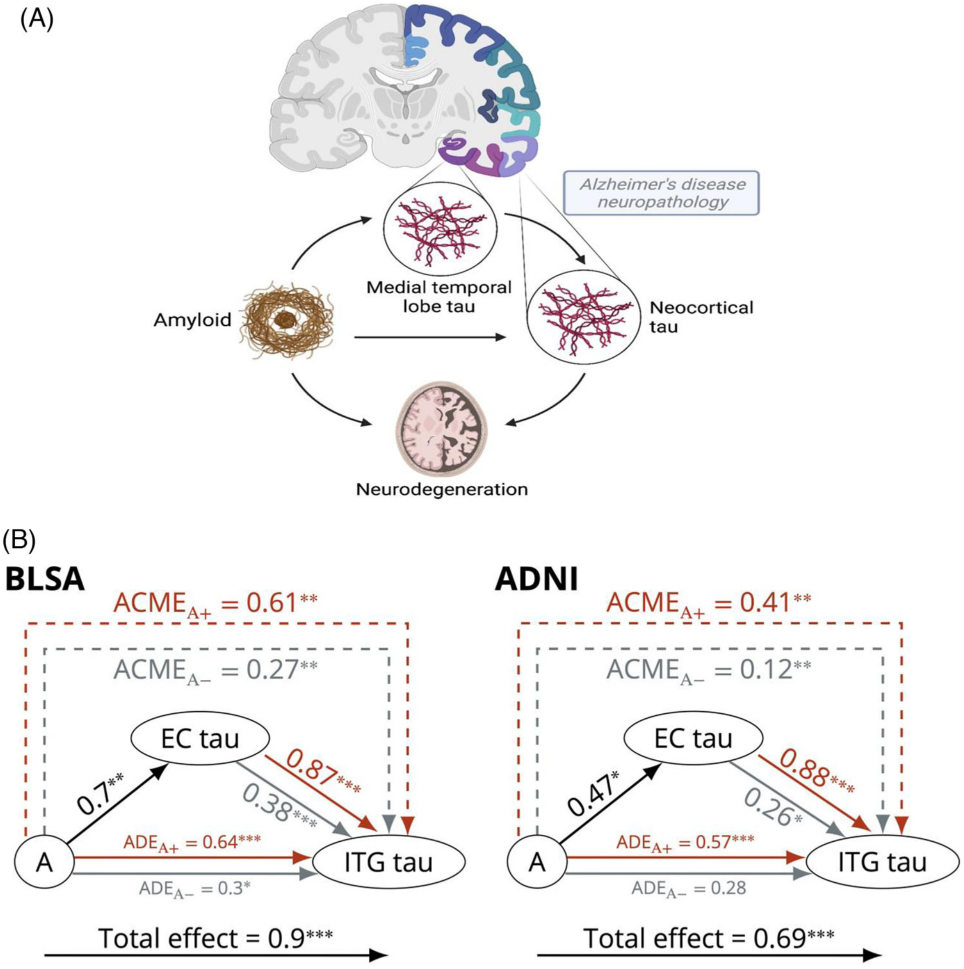

FIGURE 7 Tau neocortical progression is influenced by synergistic interaction of medial temporal lobe amyloid and tau. (A), Schematic of relationship among amyloid, medial temporal lobe tau, neocortical tau, and neurodegeneration. (B), Causal mediation analysis results for the model investigating the relationships among amyloid groups (+/–) and tau in the EC and the ITG in the BLSA and ADNI. The effect of amyloid on ITG tau is mediated by EC tau. Arrow from A to EC tau indicates the linear regression coefficient, and the gray and red arrows from EC tau to ITG tau indicate the linear regression coefficients for the amyloid negative and positive groups, respectively. Both the mediator and the outcome models were adjusted for age, sex, *APOE* ε4 positivity, years of education, and 10-year cardiovascular disease risk. ***$P < 0.001$, **$P < 0.01$, *$P < 0.05$. A, amyloid; ACME, average causal mediation effect; ADNI, Alzheimer's Disease Neuroimaging Initiative; *APOE*, apolipoprotein E; BLSA, Baltimore Longitudinal Study of Aging; EC, entorhinal cortex; ITG, inferior temporal gyrus. Reproduced from Bilgel et al.[217]

proteomics data combined with imaging modalities used in recent studies, often as part of larger consortia such as CHARGE, have revealed contributors to neurodegeneration and cognition, and determined underlying mechanisms.

In most cases of AD, the amygdala and hippocampus are sites of very early atrophy in disease progression. When machine learning was used to reprioritize hits from a GWAS of hippocampal and amygdala atrophy in AD, genes from several Aβ-related pathways predominated:[224] (1) alteration of synaptic structure and function through effects on the cytoskeleton, (2) changes in intracellular calcium levels resulting in excitotoxicity, (3) apoptotic signaling via protein misfolding in the endoplasmic reticulum, and (4) transcriptional regulation (Figure S30 in supporting information). A possible causal association mechanism underlying hippocampal volume was identified using ADNI multiomics data from blood and hippocampal tissues combined with causal association tests.[225] A non-coding SNP, rs1053218, was proposed to induce a specific DNA methylation change that hyperactivated *ANKRD37*, a gene involved in cell response to hypoxia, resulting in low hippocampal volume. The exact mechanism by which *ANKRD37* may exert its effect on hippocampal volume is unknown, but hypoxia can impair BBB function, accelerate Aβ accumulation, and increase tau hyperphosphorylation. These studies underscore the complexity of mechanisms underlying neurodegeneration.

As described in Section 2.5.5, biomarkers of neurodegeneration are not highly concordant as they reflect different, partially overlapping processes. An established neurodegeneration marker is temporoparietal hypometabolism on FDG PET. A cross-sectional study of symptomatic participants from ADNI and the University of California San Francisco found that hypometabolism in this region was associated with both local cortical thickness and tau PET SUVR but not Aβ PET.[226] Remote brain regions with strong structural connections, such as the MTL, did not strongly influence temporoparietal hypometabolism,

suggesting that hypometabolism in this region primarily reflects local tau deposition and atrophy.[226] Networks of hypometabolism became progressively disrupted during disease progression in a longitudinal study of CU participants,[227] with the decreasing network modularity and strength of connections particularly pronounced in women. Eight major AD risk alleles (*APOE*, *SORL1*, *CD33*, *FERMT2*, *TREM2*, *MEF2C*, *CLU*, and *BIN1)* significantly correlated with these metabolic networks, most strongly in the MTL. Four of these genes are involved in immune response, suggesting that inappropriate immune activation may underlie hypometabolism.

A GWAS of regional cortical thickness combined with gene expression analysis identified two novel susceptibility loci underlying brain atrophy.[228] *ST18* encodes a zinc finger transcription factor that is highly expressed in the neocortex and, in fibroblasts, pierced influenced the expression of TNF-α, IL-1α, and IL-6. *NF1A* is highly expressed in astrocytes, implying a role in neuroinflammation.

A genome-wide association meta-analysis conducted in > 53,000 non-demented adults in the CHARGE consortium aimed to identify genetic variants underlying verbal short-term memory and learning.[229] The study identified four novel loci: (1) an intronic SNP in *CDH18*, expressed in the brain and involved in maintenance of neuronal and synaptic structure and neuronal cell adhesion; (2) 14 SNPs in high linkage disequilibrium in 3p21.1, a transcriptionally active region expressed in brain tissues and containing *NT5DC2* and *STAB1*; (3) 37 SNPs in an intergenic region in 13q21; and (4) an SNP in the *APOE–TOMM40–APOC1* locus at 19q13.3. Many of these common SNPs have been linked to other neurocognitive conditions such as schizophrenia, learning difficulties, and anorexia nervosa. ADNI data were also included in gene-based GWAS of longitudinal episodic memory in seven consortia that notably included an ethnically diverse population.[230] The study identified *DCDC2* as associated with the maintenance of episodic memory in *APOE* ε4 non-carriers. Finally, a GWAS of the Trail Making Test Part A conducted in ADNI identified a novel locus in *INSC*, a gene involved in cytoskeleton organization, which was also associated with other cognitive measures. The loci identified by the studies may represent new avenues of investigation for facilitating precision medicine interventions.

3.2.10 | Insights into cognitive decline

Subjective cognitive decline (SCD), that is, self-reported worse cognitive performance, is measured in ADNI using self-report and informant report ECog. While SCD has been associated with an increased risk of AD, studies of its association with underlying AD pathology have been mixed. This may be due to differences in definitions and assessments of SCD between cohorts as rates of Aβ positivity varied widely (10% to 76% at age 70) in SCD participants from cohorts in the 20 Amyloid Biomarker Study, which included ADNI.[231] Specific characteristics of SCD (memory complaints, attention/concentration complaints, and informant confirmed complaints) were associated with Aβ positivity only in research settings including ADNI and not in memory clinics.[231] In the ADNI cohort alone, self-reported word finding complaints such as "forgetting the names of objects" measured in the ECog-Language subscale predicted lower CSF Aβ42 levels and correlated with regional atrophy.[232]

Memory awareness, defined as the difference between subjective and objective cognitive decline, decreases with increasing cognitive impairment.[233,234] In the IMAP cohort with replication in ADNI, subjective memory decline remained similar across increasingly impaired diagnostic groups whereas objective memory decline increased.[233] In CU participants, greater subjective memory decline was associated with greater neurodegeneration but the opposite was true in later diagnostic stages (Figure S31 in supporting information).[233] Informant reports of complaints across the four domains of ECog were lower than self-reported complaints in CU participants, in good agreement in MCI participants, and higher in AD participants.[234] Anosognosia (lack of awareness of impairment) was associated with greater tau but not Aβ deposition in impaired subjects.[235] These reports suggest that anosognosia increases over time with increasing tau deposition and neurodegeneration and that subjective memory complaints should be interpreted differently in those with normal cognition compared to those with dementia.

An objective measure of subtle cognitive impairment (SCI) is included in the NIA-AA staging framework for preclinical AD. In ADNI CU participants, baseline scores of the modified PACC, which measures cognitive changes that do not manifest as conspicuous impairment, were associated with baseline and longitudinal Aβ deposition, suggestive of tau deposition, and associated with greater clinical progression (Figure S32 in supporting information).[236] Subtle deficits in multiple cognitive domains may pose an additional risk of future decline. ADNI CU participants with deficits in both memory and executive function performance on MoCA[237] were associated with greater global Aβ burden and entorhinal cortical and hippocampal atrophy than those with only memory deficits.[238]

These studies support the idea that subtle cognitive changes, both subjective and objective, occur very early in disease progression and are associated with AD pathology.

3.3 | Contribution of cerebrovascular disease to disease progression

Cerebrovascular disease is a well-known risk factor for AD and is found as a co-pathology in 70% of people diagnosed with AD dementia. Risk of cardiovascular disease is associated with cerebrovascular dysfunction, and high scores on the Framingham General Cardiovascular Disease Risk Score were associated with worse baseline and longitudinal measures of glucose metabolism, executive function and other cognitive measures, and clinical progression.[239] Recent ADNI studies have continued to investigate the effects of cerebrovascular disease on disease progression using measures of CBF, an indicator of neurovascular function detected using arterial spin labeling MRI, and WMH burden, an MRI marker of small vessel cerebrovascular disease. However, these studies are limited because participants with moderate to

severe cerebrovascular disease were excluded in ADNI-1, ADNI-GO, ADNI-2, and ADNI-3.

Impaired CBF was associated with AD risk alleles.[240] An AD genetic risk score was consistently associated with worse regional CBF over the lifespan. Moreover, regions of high expression of AD risk genes overlapped with regions of worse cerebral hypoperfusion in areas affected early in the disease progression.[240] Lower baseline entorhinal, but not hippocampal, CBF predicted entorhinal cortical thinning, faster memory decline, and increasing WMH levels in non-demented participants.[241] CBF alterations at the site of early disease may precede downstream events including the development of WMH. Alternatively, early synapse loss may lead to reduced CBF. The cause–effect relationship of these factors remains unclear.

ADNI, like many AD cohorts, restricts the amount of vascular copathology in its participants. To address this limitation, one study investigated the association of WMH burden with neurodegeneration and cognition in ADNI MCI participants stratified by WMH burden, and in MCI participants from an external "real-world" cohort across the AD spectrum recruited from stroke prevention clinics.[242] Total WMH volume was associated with poor cognition, especially EF and semantic fluency, and this association was mediated primarily by cortical thickness in AD typical regions. To a much lesser degree, this association was mediated by Aβ (Figure S33 in supporting information). The authors suggested that this latter Aβ-mediated pathway reflects exacerbation of AD-related pathology via reduced Aβ clearance and/or induction of neuroinflammatory responses, as described in Sections 3.2.2 and 3.2.3. Additional support for this less prominent pathway comes from a study of CU participants[243] in which lower baseline CSF Aβ42 together with higher WMH were associated with worse memory but not executive function. Small vessel disease early in AD progression combined with abnormal Aβ may therefore promote an AD-typical disease pathway characterized by memory impairment.

In contrast, a study that followed CU, A+ MCI, and A+ AD participants for up to 13 years[244] reported that WMH burden was not only associated with memory impairment but also EF and global cognition deficits, with a maximal effect in MCI. This may represent a mixture of Aβ-dependent and -independent pathways for the effect of small vessel disease. The associations of WMH burden with deficits in different cognitive domains may be explained by their differential effect on regional cortical thickness. Global WMH were associated with lower thickness of frontotemporal regions in CU participants and cingulate regions in MCI participants, independent of regional Aβ deposition.[245] Regional WMH, when covaried for cortical atrophy patterns in A+ participants with AD dementia, accounted for the majority of the canonical AD pattern of cortical thinning except for parahippocampal and entorhinal regions and the precuneus.[246] These studies support a major contribution of WMH burden on neurodegeneration in AD.

These studies suggest that vascular dysregulation and hypoperfusion early in disease progression may be an important AD pathophysiological mechanism, influenced by AD risk alleles. This contributes to Aβ deposition, cortical atrophy, the development of regional WMH, and memory impairment early in disease progression. At the MCI stage, WMH in different regions contribute to impairments of other cognitive domains and global cognitive impairment, independent of Aβ deposition. This Aβ-dependent and Aβ-independent dichotomy of the effects of cerebrovascular disease on AD strongly supports its treatment as a therapeutic strategy.

3.4 | Biological subtypes of AD

Patients on the AD spectrum (i.e., A+), often diverge from the "typical" AD pathway described in Section 3.2. They differ in rate of decline, distribution of tau and neurodegeneration, and impairment of specific cognitive domains. What is the biological basis of these deviations? This section will discuss types of biological heterogeneity and what factors underlie these differences in disease progression. AD subtypes have been previously identified using ADNI data from a variety of modalities, primarily imaging and cognition (reviewed in Veitch et al.[8,9]). Recent studies have refined these subtypes and identified novel subtypes using CSF proteomics, lipid profiles, mitochondrial function, and comorbidities.

3.4.1 | Amyloid-based biological subtypes

Aβ may not follow a universal trajectory of accumulation assumed to begin in medial cortical regions and to spread sequentially to cortical association regions followed by occipital striatal regions. A Subtype and Stage Inference (SuStaIn) model analysis of Aβ PET scans from five cohorts including ADNI[247] recapitulated the stereotypical pattern of spread when the model was set to recover one trajectory. However, an optimized model identified three spatiotemporal subtypes based on regions of initial cortical Aβ accumulation (Figure S34 in supporting information). Approximately half of participants were assigned a subtype with an initial site of cortical deposition in the orbitofrontal region. This frontal subtype was characterized by having the highest proportion of *APOE* ε4 allele carriers, and higher Aβ and tau burden. Approximately one quarter were assigned a parietal subtype with initial Aβ deposition in the precuneus. This subtype was characterized by intermediate *APOE* ε4 allele carriage and lower age. The remainder belonged to an occipital group with the lowest *APOE* ε4 allele frequency and highest proportion of participants with dementia. The Aβ deposition trajectories of all subtypes converged at late stages of disease despite the initial differences. The association of the subtypes with cognitive impairment and underlying pathophysiology remains to be determined.

3.4.2 | Tau-based biological subtypes

The SuStain model was similarly applied to tau PET scans from five cohorts including ADNI.[248] This study identified four subtypes characterized by different spatiotemporal patterns of tau deposition (Figure S35 in supporting information). These subtypes were consistent within individuals across disease progression and different PET tracers. One

third of participants had a limbic predominant–pattern of tau progression resembling Braak staging with an estimated center of tau spreading in the EC. This limbic predominant–subtype was characterized by better global cognition than other subtypes. Slightly fewer participants were assigned a subtype with an estimated tau spreading center in the fusiform gyrus. This posterior-predominant subtype was characterized by occipital lobe binding and slower cognitive decline than other subtypes and may include the posterior cortical atrophy variant of AD. Approximately one fifth of participants had a lateral temporal subtype with an estimated center for tau spreading in the ITG and a pronounced lateralization of tau spreading from the temporal to parietal and frontal cortices. This subtype was characterized by faster cognitive decline in global cognition but not memory and may include the logopenic primary progressive aphasia AD variant. Finally, an MTL-sparing subtype with early precuneus tau binding spreading in a right-sided manner to the temporoparietal and frontal cortices was identified in 18% of participants. These individuals were younger, had worse executive function and a higher overall tau burden, and were less likely to be *APOE* ε4 allele carriers. The latter subtype may typify early-onset disease. A combined tau PET and rs-fMRI study of early versus late sporadic disease[249] examined the association of tau PET uptake with hub regions of brain functional networks. Younger age of onset, but not disease severity, was associated with a hippocampal-sparing tau PET pattern with early deposition in frontoparietal hub regions vital for cognitive function. Greater tau deposition in these hub regions was associated with faster subsequent tau accumulation and memory decline, suggesting preferential tau spread along the many connections from a key hub region.

How do tau subtypes relate to atrophy subtypes? For comparison, data-driven clusters were derived from both tau PET and structural MRI in participants with biomarker-defined AD.[250] Four clusters of tau deposition on a gradient of severity were identified. The largest cluster, Cluster I, represented early tau deposition and contained four subclusters largely congruent with those identified by Vogel et al.[248] Clusters II to IV had incrementally greater cognitive impairment and progression. Cluster II was distinguished by a high frequency of the *APOE* ε4 allele. Participants in Cluster IV were substantially younger than other clusters and had the largest brain age gap and fastest progression, likely representing a young onset group with faster tau deposition. All tau clusters were more highly associated with clinical measures than the three atrophy-defined clusters, which were described as having limbic predominant, diffuse, and hippocampal-sparing patterns of atrophy. These atrophy-based clusters were modulated to a greater degree by WMH volume and to a lesser degree by *APOE* genotype than tau-based clusters. The authors suggest that these patterns of tau spread are closely tied to clinical progression whereas those of atrophy may more closely reflect the influence of co-pathologies. Tau and atrophy-based clusters overlapped incompletely with the greatest degree of overlap between the hippocampal-sparing tau subcluster and the hippocampal-sparing atrophy cluster. This is consistent with another study demonstrating that tau PET was associated with neurodegeneration primarily within the MTL in CU participants.[251]

3.4.3 | Neurodegeneration-based biological subtypes

AD subtypes based on neurodegeneration consistently identify a "typical AD" subtype as well as a subtype that spares neurodegeneration in "typical" AD regions. The "typical" subtype derived from patterns of hypometabolism, found in approximately half of AD dementia participants, showed classic posterior temporoparietal hypometabolism with lesser MTL hypometabolism[252] (Figure S36 in supporting information). Variations in this "typical" AD pattern of hypometabolism were associated with specific impairments to memory, language, and visuospatial ability in a study of AD dementia participants stratified by primary cognitive domain impairment.[253] A second major subtype was characterized by predominant hypometabolism in limbic regions, severe memory impairments but fewer impairments in other cognitive domains, and slower progression to AD. A third minor group had a more frontal pattern of hypometabolism, and more severe EF dysfunction.[252] The limbic predominant and "typical" AD subtypes were also found in MCI participants[252,254] in whom the limbic-predominant subtype was associated with slower progression to AD and a non-AD AT(N) profile of biomarkers.[254]

Atrophy-based subtypes have been extensively studied (for previous ADNI studies, see Veitch et al.[8,9]) and validated in clinical settings after identification in research cohorts.[255] A systematic review[256] proposed that atrophy exists over two orthogonal dimensions: typicality, ranging from a "typical" AD limbic-predominant subtype to a hippocampal-sparing subtype; and severity, spanning minimal atrophy to typical AD dementia. The question of how these dimensions relate to AD pathology and additional co-pathologies was investigated using *ante mortem* MRI atrophy subtyping together with *post mortem* neuropathological analysis of participants with MCI and AD dementia.[257] Although the typicality and severity dimensions were not significantly associated, the limbic predominant subtype and typical AD subtype were more associated in a regional-specific manner with all *post mortem* pathologies considered (Aβ plaques, NFTs, TDP-43, and α-syn) than the hippocampal-sparing and minimal-atrophy subtypes (Figure S37 in supporting information). The authors suggest that despite lying on different dimensions, the limbic predominant and typical AD subtypes may be biologically more susceptible to a variety of pathologies than the hippocampal-sparing subtype, which may represent a different biological pathway. The minimal atrophy subtype may represent an early stage of an undetermined biological pathway.

The consistent reporting of subtypes with predominantly cortical or limbic atrophy may reflect underlying dissociable MTL functional networks. Disruption of functional connectivity networks is a feature of AD (for previous ADNI studies, see Veitch et al.[8,9]). The anterior temporal network may underlie limbic-predominant subtypes whereas the default mode network (DMN) may underlie cortical-predominant subtypes, with these networks having differential susceptibility to accumulation of co-pathologies.[258] Significant nodal and global changes in functional connectivity were reported in four cluster-defined AD atrophy subtypes in ADNI and German Center for

Neurodegenerative Diseases Longitudinal Cognitive Impairment and Dementia Study.[69] Subtypes with MTL predominant or diffuse atrophy had worse CSF biomarkers and pronounced cognitive decline corresponding to reduced intranetwork connectivity in the default mode, dorsal attention, visual, and limbic networks, and also with reduced global efficiency and regional clustering. These subtypes may represent a spectrum of "typical" AD. A limbic-predominant subtype with less prominent cognitive decline despite worse CSF biomarkers was characterized by substantial nodal network failure but only slight perturbations to global networks. A mild atrophy subtype with less cognitive decline showed pronounced global network failure.

Subtypes identified from clustering analysis of rs-fMRI data from the multicenter Alzheimer's Disease Imaging Consortium and replicated in ADNI had some similarities with atrophy-defined subtypes.[259] One subtype resembling "typical" AD was characterized by decreased functional connectivity in the DMN, widespread cortical thinning, and fastest cognitive decline. Another subtype, characterized by mild and diffuse functional connectivity disruption, slow decline, and local atrophy, resembled the mild atrophy subtype. A third subtype had decreased functional connectivity in the anterior cingulate cortex but increased connectivity in the prefrontal cortex, preserved hippocampal volume, and word learning but impaired visuospatial ability and severe atrophy in the anterior cingulate cortex. The authors suggest that this corresponds to the posterior subtype identified by tau PET.[248] A fourth subtype had lower word learning ability and visuospatial learning together with patterns of decreased connectivity and cortical volume loss consistent with corticobasal syndrome.

3.4.4 | The influence of co-pathologies

At autopsy, the presence of co-pathologies is so common that "pure" AD is not the most prevalent form. For instance, 42% of autopsied ADNI AD dementia cases had α-syn, found in Lewy bodies; 21% had TDP-43, found in limbic-predominant age-related TDP-43 encephalopathy (LATE); 18% had agyrophilic grain disease; and 6% had hippocampal sclerosis.[260] These pathologies may modulate the susceptibility of biological subtypes to neurodegeneration. Clustering analysis applied to tau PET and FDG PET revealed six groups that differed in degree and region of neurodegeneration, but not tau deposition.[258] The largest group had the expected hypometabolism for the level of tau. Compared to this canonical group, three groups, deemed resilient to tau deposition, had less than expected hypometabolism (two in cortical and one in limbic regions) and lower cognitive decline. Two groups, deemed susceptible to tau deposition, had worse hypometabolism than expected (one in cortical and one in limbic regions) and greater cognitive decline. The susceptible groups had a greater number of vascular risk factors. The limbic susceptible group had a pattern of hypometabolism suggestive of TDP-43. In contrast, the cortical susceptible group also had a greater burden of α-syn and other imaging markers indicative of Lewy body disease (Figure 8). The association of a pattern of MTL-sparing longitudinal cortical neurodegeneration with dementia with Lewy bodies (DLB) was also reported in a study of participants with autopsy-confirmed AD and AD with concomitant Lewy body disease.[261]

Similar canonical, resilient, and susceptible groups were identified from the degree of mismatch between tau deposition and cortical thickness.[262] Susceptible groups had greater WMH burden, and the authors suggested that the pattern of high temporal/limbic atrophy in some susceptible groups is consistent with the typical sites of TDP-43 deposition and with hippocampal sclerosis. Therefore, additional co-pathologies may contribute to heterogeneity in neurodegeneration beyond that expected from tau deposition. They may predominantly modulate neurodegeneration in regions beyond the MTL. The hippocampus, amygdala, and parahippocampal gyrus were strongly associated with clinically diagnosed dementia after accounting for not only AD neuropathology but also Lewy bodies and TDP-43.[263] Hippocampal sclerosis was the exception, impacting cognition via hippocampal atrophy.

The importance of co-pathologies in AD was underscored by a meta-analysis of cohorts including ADNI that examined links between known AD risk variants and non-AD pathologies.[264] Variants in several genes were found to be significantly associated with LATE neuropathologic changes (*GRN* and *TMEM106B*) and hippocampal sclerosis (*TNIP1* and *WNT3p*), and others trended toward association (*SORL1* and *TPCN1* with LATE neuropathologic change, *USP6NL* and *BIN1* with Lewy body pathology).

3.4.5 | Atypical AT(N) biomarker-defined groups

Several recent ADNI studies have investigated two groups defined by atypical AT(N) biomarker profiles. The first is SNAP having tau deposition and/or neurodegeneration in the absence of Aβ deposition (A–[T/N]+).[265] The second is characterized by neurodegeneration in the absence of tau despite being A+ (A+T–N+). SNAP participants with *ante mortem* MRI and autopsy data from ADNI and UPenn had a greater number of neuropathological diagnoses such as FTLD, Lewy body disease, progressive supranuclear palsy, and agyrophilic grain disease than those with biomarker-defined AD.[266] A subset of SNAP with elevated tau in the absence of Aβ deposition is consistent with primary age-related tauopathy.[267] These individuals had AD-characteristic neurodegeneration along with a higher WMH burden. The authors suggest that subthreshold Aβ may interact with cerebrovascular disease to produce an AD-like pattern of decline. In contrast, in A+T–N+ MCI participants had lower episodic memory loss and greater hippocampal volume at baseline, slower decline across all cognitive domains, and less widespread cortical thinning restricted to the MTL than A+T+(N)+ individuals. These characteristics had more in common with A–T–N+ MCI participants, suggesting that a non-AD neuropathological process is driving neurodegeneration in T– MCI subjects. The authors suggest that non-AD pathologies such as cerebrovascular disease, LATE, or Lewy body disease may drive the majority of neurodegeneration in these individuals on top of a background of preclinical AD.[239]

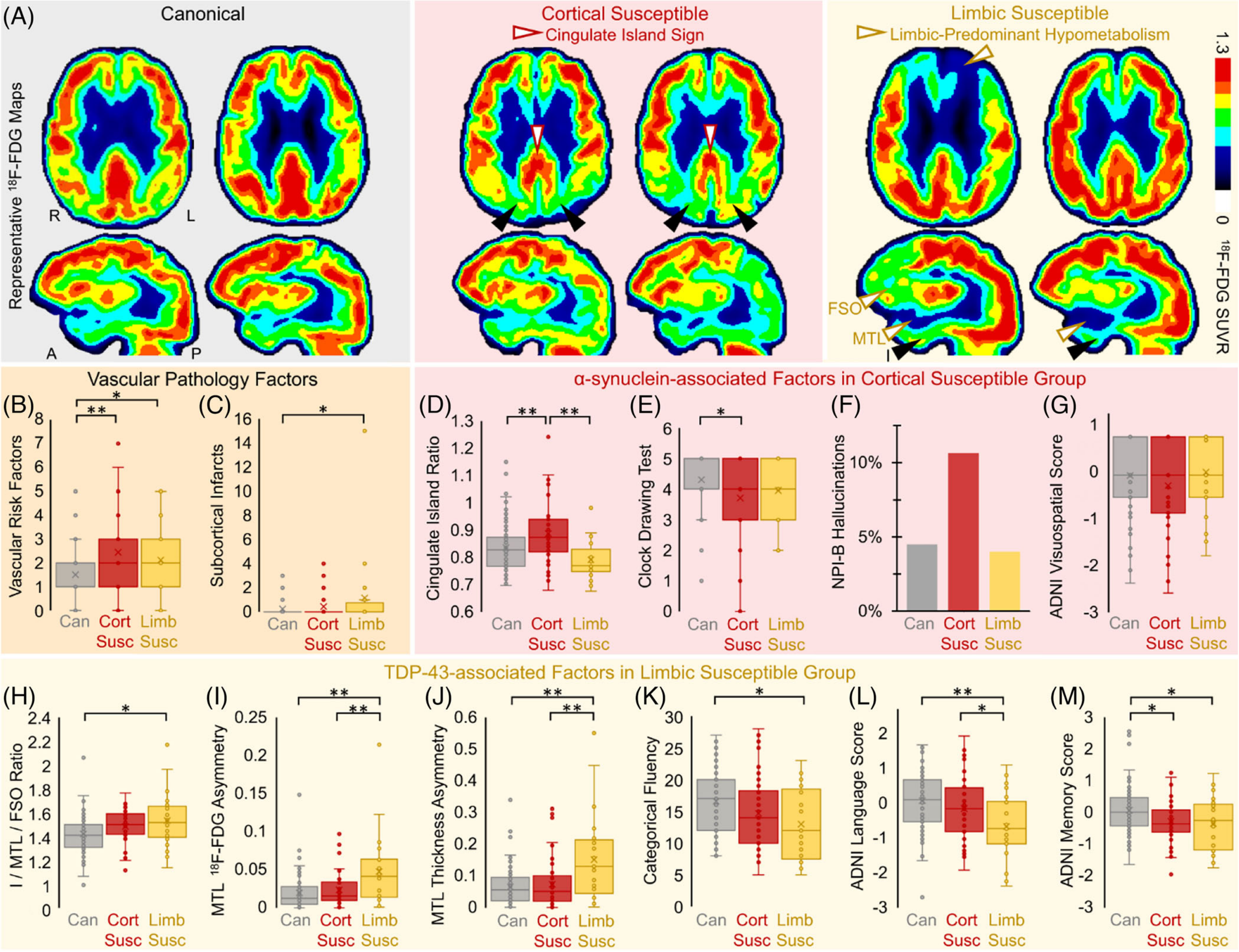

FIGURE 8 The dissociation of tau pathology and neuronal hypometabolism is related to co-pathologies. (A), Representative ^{18}F-FDG SUVR images from six patients. Susceptible patients shown here have imaging findings consistent with co-pathology (sagittal views are slices through the right hemisphere). The cortical susceptible group (middle) had participants with cingulate island sign, the sparing of posterior cingulate cortex (white arrowheads) relative to cuneus (black arrowheads). The limbic susceptible group (right) had participants with MTL and FSO ^{18}F-FDG hypometabolism (white arrowheads) relative to inferior temporal gyrus (I, black arrowheads). Vascular pathology features in susceptible groups included greater (B) vascular risk factors and (C) subcortical infarcts. The cortical susceptible group (D) had higher cingulate island ratio across groups, (E) had significantly worse clock drawing scores, and trended toward (F) greater proportion of participants with hallucinations on the Neuropsychiatric Inventory (NPI) item B and (G) worse ADNI visuospatial scores than the other groups. The limbic susceptible group had larger (H) I/MTL/FSO ^{18}F-FDG ratio and worse MTL asymmetry in (I) ^{18}F-FDG SUVR and (J) thickness, and significantly worse (K) categorical fluency, (l) language, and (M) memory *z* scores. Box plots show data points as dots, mean as an X symbol, median as the middle box line, first quartile (Q1) and third quartiles (Q3) as box edges (denoting the IQR), whiskers as the minimum/maximum points and outliers based on thresholds $<$Q1 − 1.5(IQR) or $>$Q3 + 1.5(IQR). Cognitive test comparisons included Aβ status, education, sex, and age as covariates. Significant differences in pairwise comparisons by two-tailed likelihood ratio tests are denoted by $^*P < 0.05$, $^{**}P < 0.005$. Aβ, amyloid beta; ADNI, Alzheimer's Disease Neuroimaging Initiative; FDG, ^{18}F-fluorodeoxyglucose; FSO, frontal supraorbital; IQR, interquartile range; MTL, medial temporal lobe; SUVR, standardized uptake value ratio. Reproduced under open access from Duong et al.[258]

3.4.6 | AD subtypes derived from early metabolic processes

Pathophysiological processes beyond the Aβ and tau pathways or those of co-pathologies may underlie AD heterogeneity. Early disruptions to BBB function, lipid metabolism, and mitochondrial function typify disease progression, as described in Sections 3.2.2, 3.2.4, and 3.2.5. Subtypes based on distinct CSF proteomic profiles, previously identified in AD participants,[268] were detected in A− CU participants.[269] A neuronal hyperplasticity subtype with elevated levels of BACE1 had increasing CSF p-tau181 and decreasing CSF Aβ42, and a BBB dysfunction subtype characterized by neuronal hypoplasticity showed only decreasing CSF Aβ42. The early detection of the subtypes prior to CSF biomarker abnormality suggests that differences in early

hyperplasticity-related processes or BBB dysfunction may be a source of heterogeneity in disease progression. Subgroups defined by SNPs derived from mitochondrial haplogroup J, previously associated with AD risk, had differing rates of progression independently of CSF Aβ42 and p-tau181/t-tau.[270] Two subgroups with exacerbating SNPs had elevated risk of AD, small hippocampal volume, worse glucose metabolism, and greater clinical conversion than other subgroups, including one with protective SNPs. Lipidomic signatures were also associated with differing risk of clinical progression, reflecting underlying metabolic differences.[197] Five clusters of lipids were identified in CU and MCI participants, two of which were associated with a high risk of clinical progression.

3.4.7 | Summary of recent ADNI studies of AD subtypes

Recent ADNI studies have contributed considerably to our understanding of AD biological subtypes. Variability in disease progression appears to arise before AD biomarkers become abnormal, reflected in differences in lipid composition or CSF proteins. Aβ deposition may diverge from the stereotypical pattern of spread with some participants having different sites of initial cortical deposition that later converge. AD subtypes based on tau deposition and atrophy overlap to some degree, with tau deposition more associated with *APOE* ε4 genotype and atrophy influenced to a greater extent by co-pathologies. Biological subtypes are summarized in Figure S38 in supporting information, along the conceptual axes of typicality and severity. Limbic-predominant and hippocampal-sparing subtypes are consistently identified and may be related to *APOE* ε4 genotype and the effect of co-pathologies on atrophy. Other cortical predominant subtypes may represent atypical non-amnestic AD presentations.

3.5 | Resilience to cognitive decline

Just as factors such as presence of comorbidities and *APOE* ε4 genotype can exacerbate neurodegeneration and cognitive decline beyond what would be expected for a given level of AD pathology, resilience can maintain cognition or brain structure in the face of AD pathology.[256] This section will describe investigations of the effects and mechanisms of resilience using ADNI data.

3.5.1 | Effects of resilience

Cognitive reserve,[271] defined as the difference between actual and expected cognitive function for a level of AD pathology, had a differential effect depending on diagnostic status.[272] It was associated with a lower rate of progression in CU and MCI participants, but higher rate of decline in participants with AD dementia. The acceleration of decline later in disease progression may signal the point at which cognitive reserve mechanisms are "overrun" by AD pathology after initially delaying cognitive impairment. Cognitive resilience (defined in a similar way as cognitive reserve) and brain resilience (defined by hippocampal GM measures) to Aβ deposition were studied in CU individuals from the Chinese Sino Longitudinal Study on Cognitive Decline cohort and ADNI.[273] Both measures were associated with younger age, female sex, and better cognitive performance, and predicted longitudinal cognitive decline.

3.5.2 | Mechanisms of resilience

An understanding of mechanisms underlying cognitive resilience is important for clinical management of AD and for understanding disease trajectory in patients. Features of functional and structural networks may underlie cognitive resilience. A typical brain without disease has tightly connected functional networks with high intra-network connectivity and low inter-network connectivity.[274] This high system segregation is correlated with higher global cognitive performance.[274] In DIAN participants with autosomal dominant AD and in ADNI A+ participants, higher functional network segregation was associated with lower than expected episodic memory or global cognitive decline for a given temporal lobe tau burden.[274] Moreover, higher functional network segregation was associated with slower tau accumulation relative to baseline entorhinal tau burden.[223] The rs-fMRI metric reflecting segregation of functional networks may therefore be a biomarker of cognitive resilience. The protective effect of this functional network topology was highest in CU participants and diminished in MCI and AD participants, and was greatest in *APOE* ε4 non-carriers.[275] Other mechanisms of cognitive resilience to AD pathology identified in recent ADNI studies include redundancy in nodes of posterior hippocampal functional networks[276] and higher structural network efficiency.[277]

Recent ADNI studies have pointed to a genetic component of resilience. In CU participants from a multi-cohort study including ADNI, there was a dose–response of the *APOE* ε2 allele for larger GM volumes in areas related to cognitive resilience, and an opposite effect for the *APOE* ε4 allele.[278] The *APOE* ε2 allele may contribute to resilience through the lowering of global Aβ burden. *APOE* ε2 allele carriers had a lower global Aβ burden but no difference in tau burden or accumulation compared to *APOE* ε3/ε3 homozygotes.[279] Similarly, lower hippocampal volume in *APOE* ε2 allele carriers was mediated by baseline CSF Aβ42.[280] A GWAS of cognitive resilience to Aβ burden in four cohorts including ADNI identified an SNP (rs62263260) related to expression of the *SEMA5B* gene, which is involved in synaptic pruning and axonal branching.[280] Synaptic plasticity was also implicated in a second GWAS of cognitive resilience to Aβ burden[281] that identified *CNOT7* linked to synaptic plasticity and hippocampal-dependent learning and memory. This association was not due to cerebrovascular disease or increases in tau deposition. Many other loci may contribute to resilience. A GWAS across eight independent studies including ADNI was conducted in individuals who remained CU despite having the highest genetic risk outside of the *APOE* ε4 allele.[282] Thousands of SNPs were identified and from these, a polygenic resilience score was

constructed. This polygenic resilience score was positively correlated with a PRS. In other words, individuals whose cognition remained intact despite having high genetic risk for AD also had a high polygenic resilience score. The authors suggest that multiple resilience loci interact with AD risk loci to protect against AD pathology.[282]

3.5.3 | Resilience in underrepresented populations

Factors such as socioeconomic status may impact resilience. Education is often used as a proxy for cognitive reserve, and high levels of education are generally recognized as being protective. However, while educational attainment was protective against memory loss in ADNI White participants, this effect was not observed in Black participants.[283] This may reflect a lower quality of education experienced by Black/African-American older adults or a combination of other factors that contribute to ethnocultural health disparities. Caution should therefore be taken in interpreting ADNI studies of cognitive reserve using education as a proxy. This result underscores the need for a more diverse ADNI cohort to further study the differential contributors to resilience in underrepresented populations.

3.6 | Effects of the *APOE* genotype

Recent ADNI studies support a differential effect of the *APOE* ε4 and ε3 alleles compared to the protective ε2 allele in modulating disease progression. The ε4 risk allele affected hippocampal shape in a similar manner to age,[284] and was associated with greater atrophy of the cholinergic basal forebrain.[285] Both these studies suggest involvement of the ε4 allele in sites of early neurodegeneration. In non-demented older adults, higher cortical Aβ burden predicted faster hippocampal atrophy in ε4 carriers only but predicted faster cognitive decline in both ε4 carriers and non-carriers.[286] In contrast, greater WMH predicted faster hippocampal atrophy in ε4 non-carriers only.[286]

APOE ε2 carriers may have alternative pathways of disease progression. Using a discriminative event-based model to estimate the ordering of biomarkers, ε4 carriers had a sequence similar to that described in Section 3.2 in which CSF Aβ42 abnormality precedes that of CSF p-tau181 and hippocampal volume.[287] A similar, but less certain, ordering of biomarkers was estimated in ε3 homozygotes. However, the order estimated for ε2 carriers differed substantially with CSF neurogranin and MMSE predicted as early biomarkers with low confidence, and CSF p-tau181 preceding CSF Aβ42 abnormality.[287] A large overlap of genes was reported between ε3 and ε4 carriers in an omics study including ADNI data.[288] Functions of these genes were consistent with "typical" AD processes and included mitochondrial physiology, Aβ physiology, vesicle mediated transport, and the immune response. *APOE* ε2 carriers had a distinct set of genes with novel functions including chromatin remodeling and regulation.

3.7 | Effects of sex

Sex affects the prevalence and disease course of AD, and the impact of risk factors. Recent ADNI studies have described the influence of sex on modifying disease progression, important interactions with the *APOE* ε4 allele that enhance vulnerability to AD pathology, and the effect of sex-specific resilience on AD pathology.

A clustering study of MCI participants based on multimodal factors identified sex-specific groups of differing prognoses.[289] In the worst prognosis group, there were almost twice as many female than male *APOE* ε4 carriers, suggesting that the ε4 allele has a disproportionately detrimental effect in women. Women had a greater association between whole brain atrophy and functional decline than men, and female ε4 carriers had the greatest functional decline for a given level of whole brain atrophy.[290] The interaction between sex and *APOE* influences the accumulation of AD pathology. Female ε4 carriers had a faster accumulation of Aβ in the striatum, followed by accumulation of tau in limbic regions than male ε4 carriers or female ε4 non-carriers.[291] After correcting for Aβ load, female ε4 carriers compared to non-carriers had significantly higher tau burden in MTL sites corresponding to Braak stages I and II.[292] In contrast, there was no difference in regional tau burden between male ε4 carriers and non-carriers (Figure S39 in supporting information). *APOE* ε4 gene dosage further affects regional tau binding in a sex-specific manner. Male ε4 homozygotes had significantly greater regional tau binding than heterozygotes or non-carriers.[293] However, in women, this increased regional tau deposition was observed for both ε4 heterozygotes and homozygotes compared to non-carriers.[293] Female sex may therefore enhance the deleterious effect of the *APOE* ε4 on tau binding, contributing to the differential risk of AD associated with this allele in men and women. Consensus metabolic signatures differed by sex and *APOE* ε4 allele carriage and were associated with memory and ADAS-Cog,[294] suggesting differences in underlying metabolic processes. Cardiovascular risk may further modify this sex-specific effect in a three-way interaction. In a study of CU participants, high risk of cardiovascular disease was associated with higher regional tau deposition in the EC, inferior temporal cortex, and composite temporal ROIs in female but not male *APOE* ε4 carriers[295] (Figure S40 in supporting information). The authors suggest that treatment of cardiovascular risk factors such as hypertension may be of particular benefit in female *APOE* ε4 carriers.

Differences in resilience may also contribute to the greater vulnerability of women to AD neuropathology. A study of sex differences in genetic architecture of cognitive resilience in four cohorts including ADNI found that resilience is highly heritable.[296] The study identified female-specific genetic architecture that overlapped with autoimmune disorders such as lupus and multiple sclerosis. These disorders also have a higher prevalence in women and this overlap may implicate a role of the immune system in resilience. The authors suggest that increased neuroinflammation in women associated with age-related metabolic shifts may underlie resilience. In men, specific loci were related to heart rate variability, a marker of good heart health

suggesting that cardiovascular-related pathways may underlie resilience in men.

3.8 | Involvement of NPS

NPS such as apathy, depression, delusions, anxiety, and hallucinations are frequently reported in MCI and are associated with greater cognitive and functional impairment. Recent ADNI studies have assessed associations among AD pathology, NPS, and cognitive and functional decline.

Late-life depression was associated with higher rates of domain-specific cognitive deficits in verbal learning and memory compared to non-depressed older adults with matched memory impairment and Aβ burden.[297] These deficits partially overlapped with deficits in cognitive domains associated with AD biomarkers, and the authors suggested that a portion of the cognitive impairment associated with late-life depression could be attributed to AD.[297] In a study including multimodal data from ADNI and two other cohorts, two dimensions of heterogeneity associated with late-life depression were identified.[298] The first dimension had relatively preserved brain anatomy while the second had widespread atrophy and WM disruptions, greater depression and cognitive impairment, and a higher degree of progression to AD dementia.[298] It remains to be determined whether late-life depression is a risk factor for or a prodromal feature of AD.

NPS have complex associations with Aβ pathology. Depressive symptoms are associated with a high risk of cognitive decline and increased progression to AD, with symptoms becoming frequent in MCI.[299] A study of CU participants in the Age Well cohort with replication in ADNI reported that subclinical depressive symptoms were associated with lower hippocampal volume and glucose metabolism in the frontolimbic network, the site of processes related to depressive symptoms.[299] However, these participants did not have increased brain Aβ, leading the authors to postulate that mechanisms such as neuroinflammation and early tau aggregation may link subclinical depressive symptoms with hippocampal degeneration. In contrast, other studies suggest that brain Aβ contributes to the effect of subclinical depression on cognition. In CU participants, the greater cognitive decline associated with higher rates of increase in the Geriatric Dementia Scale was mediated by worse hypometabolism and Aβ accumulation.[300] When ADNI non-demented participants were stratified using AT(N) biomarkers, subclinical depressive symptoms were associated with both memory and executive function in those classified as A+ or SNAP (A–[T/N]+) but not in those with normal biomarkers.[301]

Apathy was associated with faster cognitive and functional decline in non-demented participants, and this was mediated by Aβ deposition in prefrontal regions.[302] In addition, Aβ burden was a risk factor for apathy syndrome, suggesting a bidirectional relationship influencing early disease progression.[302] Aβ pathology also partially mediated the effects of mild behavioral impairment on decline in global cognition and increased risk of clinical conversion.[303]

Tau deposition may also mediate the relationship between NPS and cognitive performance. Tau deposition in frontal, occipital, and medial temporal cortices was associated with psychosis, and accelerated cognitive and functional decline.[304] In MCI participants stratified by Aβ status and the presence or absence of NPS, a greater association between tau deposition and cognition was reported in those with NPS.[305] This association was observed in both A– and A+ participants but was stronger in the presence of Aβ deposition. Similarly, Aβ positivity enhanced the associations between tau binding in the EC and precuneus, and affective NPS such as depression, apathy, and anxiety in MCI and AD participants.[306]

All CSF AT(N) biomarkers (Aβ42, p-tau181, and t-tau) were associated with a distinct class of depression and apathy identified from clustering analysis using the Neuropsychiatric Inventory in participants from ADNI and NACC.[307] Over 5 years, the study identified classes resulting from, rather than contributing to, stable, decreasing, and increasing depression and apathy. Worse CSF biomarkers were associated with the class of increasing depression and apathy characterized by a high prevalence of AD dementia, lower MMSE, and greater use of psychotropic medications. The authors suggest that these NPS, like cognitive decline, result from the AD disease process.

4 | ARE ADNI RESULTS GENERALIZABLE?

Given the influence of ADNI studies on clinical trials of AD therapeutic interventions, the development of prognostic algorithms, and our understanding of disease progression, a critical question is: To what extent are ADNI results generalizable to the wider population, given the lack of ethnocultural and educational diversity in the ADNI cohort? Recent ADNI studies have sought to answer this question, highlighting differences between ethnocultural groups, and have plotted a course for the next iteration of ADNI that aims to address these concerns.

4.1 | Generalizability of ADNI results

The ADNI cohort is not representative of the general elderly population. Compared to US census data, the ADNI cohort underrepresents the Asian (2% vs. 4.7%), Black/African American (5% vs. 10%), and Hispanic/Latinx (4% vs. 9.2%) populations, and those with $\leq$ 12 years of education (16% vs. 43.7%), and overrepresents the non-Hispanic White population (87% vs. 74.6%).[308] Ethnocultural and socioeconomic groups differ in the incidence and prevalence of AD, medical and biological risk factors, clinical and neuropathological features, and survival due to a range of genetic, behavioral, and sociocultural factors.[308] A comparison of associations between risk factors, and neuroimaging outcomes and cognition in ADNI and in a more diverse, randomly selected sample from four communities in North Carolina, Mississippi, Minnesota, and Maryland, comprising the ARIC cohort, explored the extent to which ADNI results are generalizable.[309] The ARIC cohort has six times the number of Black/African-American participants as ADNI (23.7% vs. 4%). One third of associations between predictors and outcomes across all modalities differed between the two cohorts (Figure S41 in supporting information). Because The ARIC

cohort is drawn from limited geographic areas, caution should be taken in extending these findings to the broader US population. However, the association between MTL structures with MMSE determined in ADNI was only partially generalizable to a Japanese cohort,[310] suggesting the issue is widespread. Further comparisons of ADNI results with diverse cohorts are required to determine its extent.

Several studies have investigated effects of ADNI's strict inclusion and exclusion criteria. Cut-points for the ADNI amnestic MCI inclusion criteria based on Story A from the Wechsler Memory Scale Logical Memory II were reported to be higher than demographically adjusted normative data.[311] The authors suggest that ADNI MCI participants may have milder memory problems than the amnestic MCI participants diagnosed using memory test cut-points based on demographically adjusted normative data, limiting the generalizability of results from studies using these participants to the wider community. A critical step in establishing clinical utility of prediction models is external validation in independent and identically distributed samples. A comparison of six clinical AD cohorts including ADNI[312] reported a wide range of probabilities of clinical progression over 10 years (Figure S42 in supporting information), which the authors attributed to differences in recruitment strategies. However, ADNI's inclusion and exclusion criteria may not fully explain issues of generalizability. Although individuals who failed the ADNI screening process were less educated and younger than those who passed, rates of screen fails did not differ between underrepresented groups and overrepresented groups.[308]

4.2 | Studies of ethnocultural differences

The issue of generalizability has also been highlighted in recent investigations of ethnocultural differences in AD risk factors, genetics, and biomarkers, both within the ADNI cohort and compared to cohorts from diverse populations. Analyses within ADNI are limited by small sample sizes of underrepresented populations, leading to variable results. No differences were reported in baseline CSF biomarkers (Aβ42, p-tau181, t-tau) or plasma p-tau181 or NfL in any ethnocultural group within ADNI.[313] However, other studies reported differences in AD biomarkers. Latinx and Asian ADNI participants had 64% and 46%, respectively, reduced odds of Aβ positivity assessed by PET compared to non-Hispanic Whites.[308] Among Black/African-American ADNI participants, levels of CSF p-tau181, t-tau, and NfL were lower compared to non-Hispanic White participants, although CSF Aβ42 levels did not differ (Figure S43 in supporting information).[314] In the same study, Black/African-American participants had significantly lower levels of CSF sTREM2, a biomarker of microglial activation, and had higher frequencies of genetic variants in *TREM2* and *MS4A4A* associated with these lower levels (Figure S43). The authors suggest that these differences in biomarkers may indicate differences in the chain of events leading to neurodegeneration between ethnocultural groups.

AD biomarkers and genetic contributors have been compared between ADNI and Korean and Han Chinese cohorts. CU participants in a Korean cohort had a lower frequency of Aβ positivity (20%) compared to ADNI CU non-Hispanic Whites (32.8%).[315] The *APOE* ε4 allele impacted Aβ status similarly in both cohorts, but polymorphisms in *BDNF* correlated with Aβ status in Koreans only, suggesting genetic differences may underlie differences in Aβ positivity frequencies. Normative data for many cortical and subcortical structures differed between ADNI and OASIS non-Hispanic White participants and those in a second Korean cohort, affecting the performance of predictive algorithms.[316] Beyond the *APOE* ε4 allele, additional predictive associations were reported between alleles in *ABCA7* and *SORL1* and cognitive decline in a Han Chinese population from Taiwan compared to ADNI.[317]

Differences in the prevalence of risk factors for AD have been identified among ethnocultural groups. Black/African-American ADNI participants had greater total and regional WMH burden compared to non-Hispanic White participants, largely attributable to the higher rate of vascular risk factors in Black/African-American groups.[318] A Puerto Rican cohort from Boston had five times the prevalence of T2DM and nearly double that of hypertension compared to ADNI non-Hispanic White participants.[319] Those participants in the Puerto Rican cohort with comorbid T2DM and hypertension had a greater brain age gap, more hippocampal atrophy, reduced regional WM fiber integrity, and lower MMSE scores than those with no comorbidities, and were clinically comparable to the ADNI progressive MCI group. Cerebrovascular risk factors that may be elevated in different ethnocultural groups due to structural and social determinants of health are therefore important treatment targets.

Together, these studies highlight the limitations of the lack of diversity of the ADNI cohort. This prevents an understanding of ethnocultural differences and sociocultural factors involved in disease risk and progression, and of how different groups might respond to therapeutic interventions.

4.3 | The future of ADNI

In September 2022, ADNI transitioned to its next 5-year phase, termed ADNI-4, entirely funded by the National Institute on Aging.[15] ADNI-4 represents a concerted effort to improve the generalizability of ADNI results by enrolling 50% to 60% of its new participants from underrepresented populations. Its enrollment approach is based on strategies tested through the ADNI-3 Diversity Task Force Project and the Brain Health Registry's Community Engaged Digital Alzheimer's Research (CEDAR) study[320] and includes a culturally informed, community-engaged research (CER) for digital and in-person outreach, engagement, and retention efforts as well as several key methodological changes. For instance, ADNI-4 uses relaxed inclusion/exclusion criteria that will allow the enrollment of some middle-aged and older adults with cardiovascular disease (although people with large strokes will continue to be excluded), and lumbar punctures and study partners are now optional. Moreover, ADNI-4 participants will now be systematically paid for their time volunteering in the study and will receive feedback on their study results if they wish to receive it. Last, ADNI-4 includes measures of sociocultural determinants of health (e.g., discrimination, acculturation, socioeconomic status).

A new Engagement Core was formed to lead ADNI-4's new brain health equity aims, which are to make ADNI more ethnoculturally, socioeconomically, and geographically diverse; to examine the biological and sociocultural determinants of brain health equities; and to improve health equity training for study personnel and scientists. In close partnership with ADNI-4's Administrative and Clinical Cores, the Engagement Core aims to enroll an initial, diverse cohort of 20,000 to be screened using an online portal.[15] Of these, 4000 participants including 50% to 60% from underrepresented populations will complete remote blood collection and screening for plasma biomarkers and *APOE*. A final in-clinic cohort of 1000 (500 new and 500 rollover participants), also comprising 50% to 60% from underrepresented populations, will complete further evaluations in a similar vein to previous ADNI phases with some technological improvements. It is hoped that ADNI-4 will improve our understanding of sociocultural contributors to heterogeneity of disease progression and provide a more diverse cohort for external validation of results, setting an example for other clinical research cohorts.

5 | CONCLUSIONS

In 2021 and 2022, ADNI has continued to profoundly impact the development of therapeutic interventions for AD and contribute to our understanding of disease progression. ADNI studies have reported improvements to clinical trial design through more focused participant selection and the detection of treatment effects in early disease. ADNI data have provided the basis for modeling tools for clinical trials, and the development of harmonization methods for PET scans and fluid biomarker cut-points. ADNI plasma samples have been instrumental in the development of plasma p-tau as a blood biomarker for AD. Machine learning-based algorithms have used ADNI AT(N) biomarker data to predict future decline, and studies have investigated whether inflammation biomarkers may add additional prognostic value, acknowledging the critical role of microglial-mediated inflammation in response to Aβ deposition. Several studies demonstrated that different modalities of AT(N) biomarkers are discordant, particularly those of neurodegeneration and to a lesser extent, tau. The development of online sensitive cognitive tests in CU participants for remote screening represents an innovative approach to substantially lower the cost of enrollment into research and clinical cohorts. The development of algorithms for individualized predictions of cognitive decline, and operationalization as online tools represents important steps toward personalized medicine.

Recent ADNI studies have improved our understanding of the multifactorial contributions to disease progression. Considerable evidence from these studies supports a cascade of events central to the amyloid hypothesis, from the disruption of Aβ homeostasis to microglial-induced neuroinflammation to perturbation of signaling pathways and metabolism to tau phosphorylation to disruption of synapses and the transneuronal spread of tau to glucose hypometabolism and neuronal cell death to neurodegeneration and cognitive impairment.

Beyond the Aβ cascade, ADNI studies have detailed myriad additional factors that may account for the observed heterogeneity in clinical manifestation and in underlying distributions of AD pathology. Vascular factors may affect disease progression via multiple mechanisms including perturbation of CBF early in disease, and the exacerbation of regional neurodegeneration and decline in specific cognitive domains by regional WMH burden via distinct Aβ-dependent and -independent pathways. Biological AD subtypes have been identified based on Aβ status, influenced by the *APOE* ε4 allele. A large body of work described subtypes based on regional tau deposition, associated primarily with clinical characteristics and *APOE* ε4 status, and on neurodegeneration, influenced by co-pathologies. A "typical AD" atrophy subtype and one with minimal atrophy may exist along the dimension of severity, while subtypes with predominantly MTL involvement or cortical involvement may exist along the orthogonal dimension of typicality. Subtypes may reflect the combined influence of genetics and co-pathologies such as vascular disease, Lewy body disease, and TDP-43 proteinopathies. Cognitive resilience appears to buffer the effect of co-pathologies and genetic risk in accelerating cognitive decline. ADNI studies have probed mechanisms underlying cognitive resilience including genetic contributions and topologies of structural and functional networks. Distinct pathways of neurodegeneration were described for carriers of the *APOE* ε2 allele and in female *APOE* ε4 carriers who had the fastest overall decline.

Comparisons of associations reported in the ADNI cohort with those in other cohorts revealed that the generalizability of these results is limited. Genetic, biomarker, and risk factor differences in underrepresented populations compared to the non-Hispanic White majority in ADNI emphasized the need for a more diverse cohort. A cohort more representative of the general population is critical for the external validation results, to understand differences in disease progression and response to therapeutic interventions, and to better understand the implications of health inequities that result from a high prevalence of modifiable risk factors in underrepresented populations. ADNI-4, the current 5-year study, aims to address the lack of diversity in the ADNI cohort to increase the generalizability and veracity of results.

AFFILIATIONS

[1]Department of Veterans Affairs Medical Center, Northern California Institute for Research and Education (NCIRE), San Francisco, California, USA

[2]Department of Veterans Affairs Medical Center, Center for Imaging of Neurodegenerative Diseases, San Francisco, California, USA

[3]Department of Radiology and Biomedical Imaging, University of California, San Francisco, California, USA

[4]Department of Medicine, University of California, San Francisco, California, USA

[5]Department of Psychiatry and Behavioral Sciences, University of California, San Francisco, California, USA

[6]Department of Neurology, University of California, San Francisco, California, USA

[7]Alzheimer's Therapeutic Research Institute, University of Southern California, San Diego, California, USA

[8]Division of Biostatistics, Department of Public Health Sciences, University of California, Davis, California, USA

[9]Division of Genetics, Department of Medicine, Brigham and Women's Hospital, Broad Institute Ariadne Labs and Harvard Medical School, Boston, Massachusetts, USA

[10]Department of Radiology, Mayo Clinic, Rochester, Minnesota, USA

[11]Helen Wills Neuroscience Institute, University of California Berkeley, Berkeley, California, USA

[12]Knight Alzheimer's Disease Research Center, Washington University School of Medicine, Saint Louis, Missouri, USA

[13]Department of Neurology, Washington University School of Medicine, Saint Louis, Missouri, USA

[14]Department of Pathology and Immunology, Washington University School of Medicine, Saint Louis, Missouri, USA

[15]Department of Radiology and Imaging Sciences and the Indiana Alzheimer's Disease Research Center, Indiana University School of Medicine, Indianapolis, Indiana, USA

[16]Center for Computational Biology and Bioinformatics, Indiana University School of Medicine, Indianapolis, Indiana, USA

[17]Wisconsin Alzheimer's Disease Research Center and Department of Medicine, University of Wisconsin School of Medicine and Public Health, Madison, Wisconsin, USA

[18]Department of Neurology, Mayo Clinic, Rochester, Minnesota, USA

[19]Department of Psychology, Latin American and Latino Studies Institute, African and African American Studies, Fordham University, New York, New York, USA

[20]Department of Neurology, Icahn School of Medicine at Mount Sinai, New York, New York, USA

[21]Department of Medical and Molecular Genetics, Indiana University School of Medicine, Indianapolis, Indiana, USA

[22]Department of Pathology and Laboratory Medicine and the PENN Alzheimer's Disease Research Center, Center for Neurodegenerative Research, Perelman School of Medicine, University of Pennsylvania, Philadelphia, Pennsylvania, USA

[23]Laboratory of Neuro Imaging, Institute of Neuroimaging and Informatics, Keck School of Medicine of University of Southern California, Los Angeles, California, USA

ACKNOWLEDGMENTS

This work was supported by NIH grant U19 -AG 024904 funded by the National Institute on Aging to Dr. Michael Weiner. This funding source had no involvement in the writing of this review.

CONFLICT OF INTEREST STATEMENT

Dr. Ashford, Dr. Beckett, Dr. Jack, Dr. Miller, Dr. Morris, Dr. Nho, Dr. Okonkwo, Dr. Perrin, Dr. Toga, Dr. Tosun, and Dr. Veitch have no conflicts to declare. Dr. Aisen has research grants from NIH, Lilly, and Eisai, and consults with Merck, Roche, Genentech, Abbvie, Biogen, ImmunoBrain Checkpoint, and Arrowhead. Dr. Green has received compensation for advising the following companies: Allelica, Atria, Fabric, Genome Web, Genomic Life, Grail, Verily, and VinBigData and is co-founder of Genome Medical and Nurture Genomics. Dr. Harvey serves as a Statistical Advisor for *PLOS ONE*. Dr. Jagust serves as a consultant to Eisai, Roche, Biogen, Prothena, and Lilly. Dr. Landau has received travel funding from the Alzheimer's Association, served on the Scientific Advisory Board and DSMB for KeifeRx, and has received speaker fees from Eisai, Inc. Dr. Nosheny receives support in the form of grants to UCSF from NIH, The Alzheimer's Association, and Genentech, Inc. Dr. Petersen has consulted for Roche, Inc., Merck, Inc., Biogen, Inc., Eisai, Inc., Nestle, Inc., and Genentech, Inc. Dr. Rivera Mindt serves on the following boards: ALL-FTD External Advisory Board, Brown University Carney Center, Harlem Community and Academic Partnership, Mayo Clinic Alzheimer's Disease Research Center Advisory Board, National Centralized Repository for AD RD (NCRAD) Committee, Natives Engaged in Alzheimer's Research (NEAR) Study Data Safety Monitoring Board, South Texas Alzheimer's Disease Research Center (ADRC) External Advisory Board, University of California, San Francisco Alzheimer's Disease Research Center Advisory Board, University of Texas Rio Grande Valley Resource Center For Minority Aging Research Advisory Board, and University of Washington ADRC External Advisory Board. Dr. Saykin has received support from Avid Radiopharmaceuticals, a subsidiary of Eli Lilly (in kind contribution of PET tracer precursor) and consulting fees from Bayer Oncology (Scientific Advisory Board), Eisai (Scientific Advisory Board), Siemens Medical Solutions USA, Inc. (Dementia Advisory Board), Springer-Nature Publishing (Editorial Office Support as Editor-in-Chief, *Brain Imaging and Behavior*). Dr. Shaw receives support Roche (IIS and in-kind reagents and instrumentation support for CSF AD biomarkers); he has received honoraria from Roche, Biogen, and Fujirebio for participation in teaching programs; and served on Advisory Boards for Roche and Biogen. Dr. Weiner serves on Editorial Boards for *Alzheimer's & Dementia*, *MRI* and *TMRI*. He has served on Advisory Boards for Acumen Pharmaceutical, ADNI, Alzheon, Inc., Biogen, Brain Health Registry, Cerecin, Dolby Family Ventures, Eli Lilly, Merck Sharp & Dohme Corp., National Institute on Aging (NIA), Nestle/Nestec, PCORI/PPRN, Roche, University of Southern California (USC), NervGen. He has provided consulting to Baird Equity Capital, BioClinica, Cerecin, Inc., Cytox, Dolby Family Ventures, Duke University, Eisai, FUJIFILM-Toyama Chemical (Japan), Garfield Weston, Genentech, Guidepoint Global, Indiana University, Japanese Organization for Medical Device Development, Inc. (JOMDD), Medscape, Nestle/Nestec, NIH, Peerview Internal Medicine, Roche, T3D Therapeutics, University of Southern California (USC), and Vida Ventures. He has acted as a speaker/lecturer to The Buck Institute for Research on Aging, China Association for Alzheimer's Disease (CAAD), Japan Society for Dementia Research, and Korean Dementia Society. He holds stock options with Alzheon, Inc., Alzeca, and Anven. The following entities have provided funding for academic travel: University of Southern California (USC), NervGen, ASFNR, and CTAD Congress. Author disclosures are available in the supporting information.

ORCID

Dallas P. Veitch https://orcid.org/0000-0002-9918-0640

REFERENCES

1. Mueller SG, Weiner MW, Thal LJ, et al. Ways toward an early diagnosis in Alzheimer's disease: the Alzheimer's disease neuroimaging initiative (ADNI). *Alzheimers Dement*. 2005;1:55-66.
2. Marek K, Jennings D, Lasch S, et al. The Parkinson progression marker initiative (PPMI). *Prog Neurobiol*. 2011;95:629-635.

3. Franzmeier N, Brendel M, Beyer L, et al. Tau deposition patterns are associated with functional connectivity in primary tauopathies. *Nat Commun*. 2022;13:18.
4. Cadby G, Giles C, Melton PE, et al. Comprehensive genetic analysis of the human lipidome identifies loci associated with lipid homeostasis with links to coronary artery disease. *Nat Commun*. 2022;13:17.
5. Weiner MW, Harvey D, Landau SM, et al. Traumatic brain injury and post-traumatic stress disorder are not associated with Alzheimer's disease pathology measured with biomarkers. *Alzheimers Dement*. 2023;19:884-895.
6. Mackin RS, Insel PS, Landau S, et al. Late-Life depression is associated with reduced cortical amyloid burden: findings from the Alzheimer's disease neuroimaging initiative depression project. *Biol Psychiatry*. 2021;89:757-765.
7. Bethlehem RAI, Seidlitz J, White SR, et al. Brain charts for the human lifespan. *Nature*. 2022;604:525-533.
8. Veitch DP, Weiner MW, Aisen PS, et al. Understanding disease progression and improving Alzheimer's disease clinical trials: Recent highlights from the Alzheimer's disease neuroimaging initiative. *Alzheimers Dement*. 2019;15:106-152.
9. Veitch DP, Weiner MW, Aisen PS, et al. Using the Alzheimer's disease neuroimaging initiative to improve early detection, diagnosis, and treatment of Alzheimer's disease. *Alzheimers Dement*. 2022;18:824-857.
10. Weiner MW, Veitch DP, Aisen PS, et al. 2014 Update of the Alzheimer's Disease Neuroimaging Initiative: a review of papers published since its inception. *Alzheimers Dement*. 2015;11:e1-120.
11. Weiner MW, Veitch DP, Aisen PS, et al. The Alzheimer's disease neuroimaging initiative: a review of papers published since its inception. *Alzheimers Dement*. 2013;9:e111-e194.
12. Weiner MW, Veitch DP, Aisen PS, et al. The Alzheimer's Disease Neuroimaging Initiative: a review of papers published since its inception. *Alzheimers Dement*. 2012;8:S1-68.
13. Weiner MW, Veitch DP, Aisen PS, et al. Recent publications from the Alzheimer's disease neuroimaging initiative: Reviewing progress toward improved AD clinical trials. *Alzheimers Dement*. 2017;13:e1-e85.
14. Weiner MW, Veitch DP, Aisen PS, et al. The Alzheimer's Disease Neuroimaging Initiative 3: continued innovation for clinical trial improvement. *Alzheimers Dement*. 2017;13:561-571.
15. Weiner MW, Veitch DP, Miller MJ, et al. Increasing participant diversity in AD research: Plans for digital screening, blood testing, and a community-engaged approach in the Alzheimer's disease neuroimaging initiative 4. *Alzheimers Dement*. 2023;19:307-317.
16. Jack CR Jr., Bennett DA, Blennow K, et al. A/T/N: an unbiased descriptive classification scheme for Alzheimer disease biomarkers. *Neurology*. 2016;87:539-547.
17. Yasuno F, Minami H. Significant effects of cholinesterase inhibitors on tau pathology in the Alzheimer's disease continuum: an in vivo positron emission tomography study. *Int J Geriatr Psychiatry*. 2021;36:1274-1283.
18. Meng D, Mohammadi-Nejad AR, Sotiropoulos SN, Auer DP, Alzheimer's Disease Neuroimaging I. Anticholinergic drugs and forebrain magnetic resonance imaging changes in cognitively normal people and those with mild cognitive impairment. *Eur J Neurol*. 2022;29:1344-1353.
19. Li J, Zheng C, Ge Q, et al. Association between long-term donepezil treatment and brain regional amyloid and tau burden among individuals with mild cognitive impairment assessed using (18) F-AV-45 and (18) F-AV-1451 PET. *J Neurosci Res*. 2022;100:670-680.
20. Stage E, Svaldi D, Sokolow S, et al. Prescribing cholinesterase inhibitors in mild cognitive impairment-observations from the Alzheimer's disease neuroimaging initiative. *Alzheimers Dement (NY)*. 2021;7:e12168.
21. Deng Z, Jiang J, Wang J, et al. Angiotensin receptor blockers are associated with a lower risk of progression from mild cognitive impairment to dementia. *Hypertension (Dallas, Tex: 1979)*. 2022;79:2159-2169.
22. Weng J, Zhao G, Weng L, Guan J. Aspirin using was associated with slower cognitive decline in patients with Alzheimer's disease. *PLoS One*. 2021;16:e0252969.
23. Pomilio C, Perez NG, Calandri I, et al. Diabetic patients treated with metformin during early stages of Alzheimer's disease show a better integral performance: data from ADNI study. *Geroscience*. 2022;44:1791-1805.
24. Bouter Y, Bouter C. Selective serotonin reuptake inhibitor-treatment does not show beneficial effects on cognition or amyloid burden in cognitively impaired and cognitively normal subjects. *Front Aging Neurosci*. 2022;14:883256.
25. van Dyck CH, Swanson CJ, Aisen P, et al. Lecanemab in early Alzheimer's disease. *N Engl J Med*. 2022;388:9-21.
26. Tahami Monfared AA, Tafazzoli A, Ye W, Chavan A, Zhang Q. Long-term health outcomes of lecanemab in patients with early Alzheimer's disease using simulation modeling. *Neurolo Ther*. 2022;11:863-880.
27. Cullen N, Janelidze S, Palmqvist S, et al. Association of CSF Abeta38 levels with risk of Alzheimer disease-related decline. *Neurology*. 2022;98:e958-e967.
28. Jack CR Jr., Bennett DA, Blennow K, et al. NIA-AA research framework: toward a biological definition of Alzheimer's disease. *Alzheimers Dement*. 2018;14:535-562.
29. Josephs KA, Weigand SD, Whitwell JL. Characterizing Amyloid-positive individuals with normal tau PET levels after 5 years: an ADNI study. *Neurology*. 2022;98:e2282-e2292.
30. Klunk WE, Koeppe RA, Price JC, et al. The Centiloid Project: standardizing quantitative amyloid plaque estimation by PET. *Alzheimers Dement*. 2015;11:1-15. e4.
31. Matthews DC, Lukic AS, Andrews RD, et al. Measurement of neurodegeneration using a multivariate early frame amyloid PET classifier. *Alzheimers Dement (N Y)*. 2022;8:e12325.
32. Jutten RJ, Sikkes SAM, Van der Flier WM, Scheltens P, Visser PJ, Tijms BM. Finding treatment effects in Alzheimer trials in the face of disease progression heterogeneity. *Neurology*. 2021;96:e2673-e2684.
33. Giraldo DL, Sijbers J, Romero E, Initiative AsDN. Quantification of cognitive impairment to characterize heterogeneity of patients at risk of developing Alzheimer's disease dementia. *Alzheimers Dement*. 2021;13:e12237.
34. Berres M, Monsch AU, Spiegel R. Using historical data to facilitate clinical prevention trials in Alzheimer disease? An analysis of longitudinal MCI (mild cognitive impairment) data sets. *Alzheimers Res Ther*. 2021;13:97.
35. Kiselica AM, Benge JF, Alzheimer's Disease neuroimaging I. neuropsychological equivalence of the clinical diagnosis of mild cognitive impairment in the national Alzheimer's coordinating center uniform data set and Alzheimer's disease neuroimaging initiative. *Dement Geriatr Cogn Disord*. 2021;50:231-236.
36. Abi Nader C, Ayache N, Frisoni GB, Robert P, Lorenzi M, Alzheimer's disease neuroimaging I. Simulating the outcome of amyloid treatments in Alzheimer's disease from imaging and clinical data. *Brain Commun*. 2021;3:fcab091.
37. Clément AN, Ribaldi F, Frisoni GB, et al. SimulAD: a dynamical model for personalized simulation and disease staging in Alzheimer's disease. *Neurobiol Aging*. 2022;113:73-83.
38. Royse SK, Minhas DS, Lopresti BJ, et al. Validation of amyloid PET positivity thresholds in centiloids: a multisite PET study approach. *Alzheimers Res Ther*. 2021;13:99.
39. Bullich S, Roe-Vellve N, Marquie M, et al. Early detection of amyloid load using (18)F-florbetaben PET. *Alzheimers Res Ther*. 2021;13:67.

40. Pegueroles J, Montal V, Bejanin A, et al. AMYQ: An index to standardize quantitative amyloid load across PET tracers. *Alzheimers Dement*. 2021;17:1499-1508.
41. Weigand AJ, Maass A, Eglit GL, Bondi MW. What's the cut-point?: a systematic investigation of tau PET thresholding methods. *Alzheimers Res Ther*. 2022;14:49.
42. Dumurgier J, Sabia S, Zetterberg H, et al. Pragmatic, data-driven method to determine cutoffs for CSF biomarkers of Alzheimer disease based on validation against PET imaging. *Neurology*. 2022;99:e669-e678.
43. Shishegar R, Cox T, Rolls D, et al. Using imputation to provide harmonized longitudinal measures of cognition across AIBL and ADNI. *Sci Rep*. 2021;11:23788.
44. Hampton OL, Mukherjee S, Properzi MJ, et al. Harmonizing the preclinical Alzheimer cognitive composite for multicohort studies. *Neuropsychology*. 2023;37:436-449.
45. Salimi Y, Domingo-Fernández D, Bobis-Álvarez C, Hofmann-Apitius M, Birkenbihl C. ADataViewer: exploring semantically harmonized Alzheimer's disease cohort datasets. *Alzheimers Res Ther*. 2022;14:69.
46. Teunissen CE, Verberk IMW, Thijssen EH, et al. Blood-based biomarkers for Alzheimer's disease: towards clinical implementation. *Lancet Neurol*. 2022;21:66-77.
47. Balogun WG, Zetterberg H, Blennow K, Karikari TK. Plasma biomarkers for neurodegenerative disorders: ready for prime time? *Curr Opin Psychiatry*. 2023;36:112-118.
48. Zicha S, Bateman RJ, Shaw LM, et al. Comparative analytical performance of multiple plasma Abeta42 and Abeta40 assays and their ability to predict positron emission tomography amyloid positivity. *Alzheimers Dement*. 2023;19:956-966.
49. Udeh-Momoh C, Zheng B, Sandebring-Matton A, et al. Blood derived amyloid biomarkers for Alzheimer's disease prevention. *J Prev Alzheimers Dis*. 2022;9:12-21.
50. Li Y, Schindler SE, Bollinger JG, et al. Validation of Plasma Amyloid-beta 42/40 for detecting Alzheimer disease amyloid plaques. *Neurology*. 2022;98:E688-E699.
51. Chen SD, Huang YY, Shen XN, et al. Longitudinal plasma phosphorylated tau 181 tracks disease progression in Alzheimer's disease. *Transl Psychiatry*. 2021;11:356.
52. Wang YL, Chen J, Du ZL, et al. Plasma P-tau181 level predicts neurodegeneration and progression to Alzheimer's dementia: a longitudinal study. *Front Neurol*. 2021;12:695696.
53. Lussier FZ, Benedet AL, Therriault J, et al. Plasma levels of phosphorylated tau 181 are associated with cerebral metabolic dysfunction in cognitively impaired and amyloid-positive individuals. *Brain Commun*. 2021;3:fcab073.
54. Shen XN, Huang YY, Chen SD, et al. Plasma phosphorylated-tau181 as a predictive biomarker for Alzheimer's amyloid, tau and FDG PET status. *Transl Psychiatry*. 2021;11:585.
55. Tissot C, A LB, Therriault J, et al. Plasma pTau181 predicts cortical brain atrophy in aging and Alzheimer's disease. *Alzheimers Res Ther*. 2021;13:69.
56. Therriault J, Benedet AL, Pascoal TA, et al. Association of plasma P-tau181 with memory decline in non-demented adults. *Brain Commun*. 2021;3:10.
57. Tropea TF, Waligorska T, Xie SX, et al. Plasma phosphorylated tau181 predicts cognitive and functional decline. *Ann Clin Transl Neurol*. 2023;10:18-31.
58. Grothe MJ, Moscoso A, Ashton NJ, et al. Associations of fully automated CSF and novel plasma biomarkers with Alzheimer disease neuropathology at autopsy. *Neurology*. 2021;97:e1229-e1242.
59. Zettergren A, Lord J, Ashton NJ, et al. Association between polygenic risk score of Alzheimer's disease and plasma phosphorylated tau in individuals from the Alzheimer's disease neuroimaging initiative. *Alzheimers Res Ther*. 2021;13:17.
60. Hazan J, Alston D, Fox NC, Howard R. Practical application of Alzheimer's disease neuroimaging initiative plasma P-tau181 reference data to support diagnosis of Alzheimer's disease. *Int J Geriatr Psychiatry*. 2022;37.
61. Palmqvist S, Tideman P, Cullen N, Alzheimer's Disease Neuroimaging I, et al. Prediction of future Alzheimer's disease dementia using plasma phospho-tau combined with other accessible measures. *Nat Med*. 2021;27:1034-1042.
62. Milà-Alomà M, Ashton NJ, Shekari M, et al. Plasma P-tau231 and P-tau217 as state markers of amyloid-β pathology in preclinical Alzheimer's disease. *Nat Med*. 2022;28:1797-1801.
63. Palmqvist S, Janelidze S, Quiroz YT, et al. Discriminative accuracy of plasma phospho-tau217 for Alzheimer disease vs other neurodegenerative disorders. *JAMA*. 2020;324:772-781.
64. Janelidze S, Berron D, Smith R, et al. Associations of plasma phospho-Tau217 levels with tau positron emission tomography in early Alzheimer disease. *JAMA Neurol*. 2021;78:149-156.
65. Marks JD, Syrjanen JA, Graff-Radford J, et al. Comparison of plasma neurofilament light and total tau as neurodegeneration markers: associations with cognitive and neuroimaging outcomes. *Alzheimers Res Ther*. 2021;13:199.
66. Bangen KJ, Thomas KR, Weigand AJ, et al. Elevated plasma neurofilament light predicts a faster rate of cognitive decline over 5 years in participants with objectively-defined subtle cognitive decline and MCI. *Alzheimers Dement*. 2021;17:1756-1762.
67. Qu Y, Tan CC, Shen XN, et al. Association of plasma neurofilament light with small vessel disease burden in nondemented elderly: A longitudinal study. *Stroke*. 2021;52:896-904.
68. Moscoso A, Grothe MJ, Ashton NJ, et al. Longitudinal associations of blood phosphorylated tau181 and neurofilament light chain with neurodegeneration in Alzheimer disease. *JAMA Neurol*. 2021;78:396-406.
69. Rauchmann BS, Schneider-Axmann T, Perneczky R, Alzheimer's disease neuroimaging I. Associations of longitudinal plasma P-tau181 and NfL with tau-PET, Abeta-PET and cognition. *J Neurol Neurosurg Psychiatry*. 2021;92:1289-1295.
70. Anggono V, Tsai L-H, Götz J. *Glutamate Receptors in Alzheimer's Disease: Mechanisms and Therapies*. Hindawi; 2016:8256196
71. Tian C, Stewart T, Hong Z, et al. Blood extracellular vesicles carrying synaptic function-and brain-related proteins as potential biomarkers for Alzheimer's disease. *Alzheimers Dement*. 2023;19:909-923.
72. Silva CT, Young JI, Zhang L, et al. Cross-tissue analysis of blood and brain epigenome-wide association studies in Alzheimer's disease. *Nat Commun*. 2022;13:4852.
73. Fouladi S, Safaei AA, Arshad NI, Ebadi MJ, Ahmadian A. The use of artificial neural networks to diagnose Alzheimer's disease from brain images. *Multimed Tools Appl*. 2022;81:37681-37721.
74. Chen YR, Qian XL, Zhang YY, et al. Prediction models for conversion from mild cognitive impairment to Alzheimer's disease: a systematic review and meta-analysis. *Front Aging Neurosci*. 2022;14:11.
75. Rowe TW, Katzourou IK, Stevenson-Hoare JO, Bracher-Smith MR, Ivanov DK, Escott-Price V. Machine learning for the life-time risk prediction of Alzheimer's disease: a systematic review. *Brain Commun*. 2021;3:17.
76. Ansart M, Epelbaum S, Bassignana G, et al. Predicting the progression of mild cognitive impairment using machine learning: a systematic, quantitative and critical review. *Med Image Anal*. 2021;67:101848.
77. Grueso S, Viejo-Sobera R. Machine learning methods for predicting progression from mild cognitive impairment to Alzheimer's disease dementia: a systematic review. *Alzheimers Res Ther*. 2021;13:162.
78. Dansson HV, Stempfle L, Egilsdóttir H, et al. Predicting progression and cognitive decline in amyloid-positive patients with Alzheimer's disease. *Alzheimers Res Ther*. 2021;13:151.

79. Pfeil J, Hoenig MC, Doering E, et al. Unique regional patterns of amyloid burden predict progression to prodromal and clinical stages of Alzheimer's disease. *Neurobiol Aging*. 2021;106:119-129.
80. Kim HJ, Oh JS, Lim JS, et al. The impact of subthreshold levels of amyloid deposition on conversion to dementia in patients with amyloid-negative amnestic mild cognitive impairment. *Alzheimers Res Ther*. 2022;14:93.
81. Farrell ME, Jiang S, Schultz AP, et al. Defining the lowest threshold for Amyloid-PET to predict future cognitive decline and amyloid accumulation. *Neurology*. 2021;96:e619-e631.
82. Hu WT, Ozturk T, Kollhoff A, Wharton W, Christina Howell J. Higher CSF sTNFR1-related proteins associate with better prognosis in very early Alzheimer's disease. *Nat Commun*. 2021;12:4001.
83. Biel D, Brendel M, Rubinski A, et al. Tau-PET and in vivo Braak-staging as prognostic markers of future cognitive decline in cognitively normal to demented individuals. *Alzheimers Res Ther*. 2021;13:137.
84. Ossenkoppele R, Reimand J, Smith R, et al. Tau PET correlates with different Alzheimer's disease-related features compared to CSF and plasma P-tau biomarkers. *EMBO Mol Med*. 2021;13:e14398.
85. Therriault J, Vermeiren M, Servaes S, et al. Association of phosphorylated tau biomarkers with amyloid positron emission tomography vs tau positron emission tomography. *JAMA Neurol*. 2023;80:188-199.
86. Guo Y, Huang YY, Shen XN, et al. Characterization of Alzheimer's tau biomarker discordance using plasma, CSF, and PET. *Alzheimers Res Ther*. 2021;13:93.
87. Provost K, Iaccarino L, Soleimani-Meigooni DN, et al. Comparing ATN-T designation by tau PET visual reads, tau PET quantification, and CSF PTau181 across three cohorts. *Eur J Nucl Med Mol Imaging*. 2021;48:2259-2271.
88. Bron EE, Klein S, Papma JM, et al. Cross-cohort generalizability of deep and conventional machine learning for MRI-based diagnosis and prediction of Alzheimer's disease. *NeuroImage Clin*. 2021;31:102712.
89. Pelkmans W, Vromen EM, Dicks E, et al. Grey matter network markers identify individuals with prodromal Alzheimer's disease who will show rapid clinical decline. *Brain Commun*. 2022;4:fcac026.
90. Venkatraghavan V, Vinke EJ, Bron EE, et al. Progression along data-driven disease timelines is predictive of Alzheimer's disease in a population-based cohort. *Neuroimage*. 2021;238:118233.
91. Blazhenets G, Ma Y, Sorensen A, et al. Principal components analysis of brain metabolism predicts development of Alzheimer dementia. *J Nucl Med*. 2019;60:837-843.
92. Blazhenets G, Frings L, Ma Y, et al. Validation of the Alzheimer disease dementia conversion-related pattern as an ATN Biomarker of neurodegeneration. *Neurology*. 2021;96:e1358-e1368.
93. Yang F, Jiang J, Alberts I, et al. Combining PET with MRI to improve predictions of progression from mild cognitive impairment to Alzheimer's disease: an exploratory radiomic analysis study. *Ann Transl Med*. 2022;10:513.
94. Li Y, Haber A, Preuss C, et al. Transfer learning-trained convolutional neural networks identify novel MRI biomarkers of Alzheimer's disease progression. *Alzheimers Dement (Amst)*. 2021;13:e12140.
95. Fletcher E, Gavett B, Crane P, et al. A robust brain signature region approach for episodic memory performance in older adults. *Brain*. 2021;144:1089-1102.
96. Casanova R, Hsu FC, Sink KM, et al. Alzheimer's disease risk assessment using large-scale machine learning methods. *PLoS One*. 2013;8:e77949.
97. Casanova R, Hsu FC, Barnard RT, et al. Comparing data-driven and hypothesis-driven MRI-based predictors of cognitive impairment in individuals from the atherosclerosis risk in communities (ARIC) study. *Alzheimers Dement*. 2022;18:561-571.
98. Pinaya WHL, Scarpazza C, Garcia-Dias R, et al. Using normative modelling to detect disease progression in mild cognitive impairment and Alzheimer's disease in a cross-sectional multi-cohort study. *Sci Rep*. 2021;11:13.
99. Song YH, Yi JY, Noh Y, et al. On the reliability of deep learning-based classification for Alzheimer's disease: multi-cohorts, multi-vendors, multi-protocols, and head-to-head validation. *Front Neurosci*. 2022;16:851871.
100. Lu B, Li HX, Chang ZK, et al. A practical Alzheimer's disease classifier via brain imaging-based deep learning on 85,721 samples. *J Big Data*. 2022;9:22.
101. Bucci M, Chiotis K, Nordberg A, Alzheimer's Disease Neuroimaging I. Alzheimer's disease profiled by fluid and imaging markers: tau PET best predicts cognitive decline. *Mol Psychiatry*. 2021;26:5888-5898.
102. Lin RR, Xue YY, Li XY, et al. Optimal combinations of AT(N) biomarkers to determine longitudinal cognition in the Alzheimer's disease. *Front Aging Neurosci*. 2021;13:14.
103. Visser PJ, Reus LM, Gobom J, et al. Cerebrospinal fluid tau levels are associated with abnormal neuronal plasticity markers in Alzheimer's disease. *Mol Neurodegener*. 2022;17:27.
104. Llano DA, Devanarayan P, Devanarayan V, Alzheimer's disease neuroimaging I. CSF peptides from VGF and other markers enhance prediction of MCI to AD progression using the ATN framework. *Neurobiol Aging*. 2023;121:15-27.
105. Ezzati A, Abdulkadir A, Jack CR Jr., et al. Predictive value of ATN biomarker profiles in estimating disease progression in Alzheimer's disease dementia. *Alzheimers Dement*. 2021;17:1855-1867.
106. Vromen EM, de Boer SCM, Teunissen CE, et al. Biomarker A+T-: is this Alzheimer's disease or not? A combined CSF and pathology study. *Brain*. 2023;146:1166-1174.
107. Brum WS, de Bastiani MA, Bieger A, et al. A three-range approach enhances the prognostic utility of CSF biomarkers in Alzheimer's disease. *Alzheimers Dement*. 2022;8:e12270.
108. Smith R, Cullen NC, Pichet Binette A, et al. Tau-PET is superior to phospho-tau when predicting cognitive decline in symptomatic AD patients. *Alzheimers Dement*. 2023;19:2497-2507.
109. O'Shea DM, Thomas KR, Asken B, et al. Adding cognition to AT(N) models improves prediction of cognitive and functional decline. *Alzheimers Dement*. 2021;13:e12174.
110. Morrison C, Dadar M, Shafiee N, Villeneuve S, Louis Collins D, for Alzheimer's disease neuroimaging I. Regional brain atrophy and cognitive decline depend on definition of subjective cognitive decline. *NeuroImage Clinical*. 2022;33:102923.
111. Howell T, Neuhaus J, Glymour MM, Weiner MW, Nosheny RL. Validity of online versus in-clinic self-reported everyday cognition scale. *J Prev Alzheimers Dis*. 2022;9:269-276.
112. Bock JR, Hara J, Fortier D, Lee MD, Petersen RC, Shankle WR. Application of digital cognitive biomarkers for Alzheimer's disease: identifying cognitive process changes and impending cognitive decline. *J Prev Alzheimers Dis*. 2021;8:123-126.
113. Edgar CJ, Siemers E, Maruff P, et al. Pilot evaluation of the unsupervised, at-home cogstate brief battery in ADNI-2. *J Alzheimers Dis*. 2021;83:915-925.
114. Pyun JM, Park YH, Lee KJ, et al. Predictability of polygenic risk score for progression to dementia and its interaction with APOE epsilon4 in mild cognitive impairment. *Transl Neurodegener*. 2021;10:32.
115. Liu H, Lutz M, Luo S, Alzheimer's Disease Neuroimaging I. Association between polygenic risk score and the progression from mild cognitive impairment to Alzheimer's disease. *J Alzheimers Dis*. 2021;84:1323-1335.
116. Yang J, Wang Z, Fu Y, et al. Prediction value of the genetic risk of type 2 diabetes on the amnestic mild cognitive impairment conversion to Alzheimer's disease. *Front Aging Neurosci*. 2022;14:964463.
117. Leonenko G, Baker E, Stevenson-Hoare J, et al. Identifying individuals with high risk of Alzheimer's disease using polygenic risk scores. *Nat Commun*. 2021;12:10.
118. Iturria-Medina Y, Carbonell F, Assadi A, et al. Integrating molecular, histopathological, neuroimaging and clinical neuroscience data with NeuroPM-box. *Commun Biol*. 2021;4:20.

119. Hao W, Lenhart S, Petrella JR. Optimal anti-amyloid-beta therapy for Alzheimer's disease via a personalized mathematical model. *PLoS Comput Biol*. 2022;18:e1010481.
120. Wang M, Sajobi TT, Ismail Z, et al. A pragmatic dementia risk score for patients with mild cognitive impairment in a memory clinic population: Development and validation of a dementia risk score using routinely collected data. *Alzheimers Dement (N Y)*. 2022;8:e12301.
121. Koval I, Bone A, Louis M, et al. AD course map charts Alzheimer's disease progression. *Sci Rep*. 2021;11:16.
122. Kuhnel L, Bouteloup V, Lespinasse J, et al. Personalized prediction of progression in pre-dementia patients based on individual biomarker profile: a development and validation study. *Alzheimers Dement*. 2021;17:1938-1949.
123. Betthauser TJ, Bilgel M, Koscik RL, et al. Multi-method investigation of factors influencing amyloid onset and impairment in three cohorts. *Brain*. 2022;145:4065-4079.
124. Powell F, Tosun D, Raj A, Initiative AsDN. Network-constrained technique to characterize pathology progression rate in Alzheimer's disease. *Brain Commun*. 2021;3:fcab144.
125. Raj A, LoCastro E, Kuceyeski A, Tosun D, Relkin N, Weiner M. Network diffusion model of progression predicts longitudinal patterns of atrophy and metabolism in Alzheimer's disease. *Cell Rep*. 2015;10:359-369.
126. Cullen NC, Leuzy A, Palmqvist S, et al. Individualized prognosis of cognitive decline and dementia in mild cognitive impairment based on plasma biomarker combinations. *Nature Aging*. 2021;1:114-123.
127. Sato K, Mano T, Ihara R, et al. Cohort-specific optimization of models predicting preclinical Alzheimer's disease, to enhance screening performance in the middle of preclinical Alzheimer's disease clinical studies. *JPAD-J Prev Alzheimers Dis*. 2021;8:503-512.
128. Tosun D, Veitch D, Aisen P, et al. Detection of β-amyloid positivity in Alzheimer's disease neuroimaging initiative participants with demographics, cognition, MRI and plasma biomarkers. *Brain Commun*. 2021;3:fcab008.
129. Wu J, Dong Q, Gui J, et al. Predicting brain amyloid using multivariate morphometry statistics, sparse coding, and correntropy: validation in 1,101 individuals from the ADNI and OASIS databases. *Front Neurosci*. 2021;15:669595.
130. Giorgio J, Jagust WJ, Baker S, Landau SM, Tino P, Kourtzi Z. A robust and interpretable machine learning approach using multimodal biological data to predict future pathological tau accumulation. *Nat Commun*. 2022;13:1887.
131. Yang F, Chowdhury SR, Jacobs HIL, et al. Longitudinal predictive modeling of tau progression along the structural connectome. *Neuroimage*. 2021;237:118126.
132. Sun YQ, Wang M, Zhao YX, et al. A Pathway-specific polygenic risk score is associated with tau pathology and cognitive decline. *J Alzheimers Dis*. 2022;85:1745-1754.
133. Rubinski A, Frerich S, Malik R, et al. Polygenic effect on tau pathology progression in Alzheimer's disease. *Ann Neurol*. 2023;93:819-829.
134. Kim J, Park Y, Park S, et al. Prediction of tau accumulation in prodromal Alzheimer's disease using an ensemble machine learning approach. *Sci Rep*. 2021;11:5706.
135. Selkoe DJ, Hardy J. The amyloid hypothesis of Alzheimer's disease at 25 years. *EMBO Mol Med*. 2016;8:595-608.
136. Gonneaud J, Baria AT, Pichet Binette A, et al. Accelerated functional brain aging in pre-clinical familial Alzheimer's disease. *Nat Commun*. 2021;12:5346.
137. Huang W, Li X, Li H, et al. Accelerated brain aging in amnestic mild cognitive impairment: relationships with individual cognitive decline, risk factors for Alzheimer disease, and clinical progression. *Radiol Artif Intell*. 2021;3:e200171.
138. Taylor A, Zhang F, Niu X, et al. Investigating the temporal pattern of neuroimaging-based brain age estimation as a biomarker for Alzheimer's disease related neurodegeneration. *Neuroimage*. 2022;263:119621.
139. Milicic L, Vacher M, Porter T, et al. Comprehensive analysis of epigenetic clocks reveals associations between disproportionate biological ageing and hippocampal volume. *Geroscience*. 2022;44:1807-1823.
140. Sugden K, Caspi A, Elliott ML, et al. Association of pace of aging measured by blood-based DNA methylation with age-related cognitive impairment and dementia. *Neurology*. 2022;99:e1402-e1413.
141. Dong A, Zhang G, Liu J, Wei Z. Latent feature representation learning for Alzheimer's disease classification. *Comput Biol Med*. 2022;150:106116.
142. Kim BH, Vasanthakumar A, Li QS, et al. Integrative analysis of DNA methylation and gene expression identifies genes associated with biological aging in Alzheimer's disease. *Alzheimers Dement*. 2022;14:e12354.
143. Wesenhagen KE, Gobom J, Bos I, et al. Effects of age, amyloid, sex, and APOE ε4 on the CSF proteome in normal cognition. *Alzheimers Dement*. 2022;14:e12286.
144. Adewale Q, Khan AF, Carbonell F, Iturria-Medina Y. Integrated transcriptomic and neuroimaging brain model decodes biological mechanisms in aging and Alzheimer's disease. *eLife*. 2021;10:e62589.
145. Engvig A, Maglanoc LA, Doan NT, Westlye LT, Alzheimer's Disease Neuroimaging I. Data-driven health deficit assessment improves a frailty index's prediction of current cognitive status and future conversion to dementia: results from ADNI. *Geroscience*. 2023;45:591-611.
146. Mintun MA, Lo AC, Duggan Evans C, et al. Donanemab in early Alzheimer's disease. *N Engl J Med*. 2021;384:1691-1704.
147. Morris JC, Weiner M, Xiong C, et al. Autosomal dominant and sporadic late onset Alzheimer's disease share a common in vivo pathophysiology. *Brain*. 2022;145:3594-3607.
148. Guo T, Korman D, Baker SL, Landau SM, Jagust WJ, Alzheimer's Disease Neuroimaging I. Longitudinal cognitive and biomarker measurements support a unidirectional pathway in Alzheimer's disease pathophysiology. *Biol Psychiatry*. 2021;89:786-794.
149. Kim CM, Montal V, Diez I, Orwig W, Alzheimer's Disease Neuroimaging I. Network interdigitations of Tau and amyloid-beta deposits define cognitive levels in aging. *Hum Brain Mapp*. 2021;42:2990-3004.
150. Golriz Khatami S, Salimi Y, Hofmann-Apitius M, et al. Comparison and aggregation of event sequences across ten cohorts to describe the consensus biomarker evolution in Alzheimer's disease. *Alzheimers Res Ther*. 2022;14:55.
[Correction added on September 21, 2023, after first online publication: First author name has been corrected for reference 150.]
151. Jack CR Jr., Knopman DS, Jagust WJ, et al. Tracking pathophysiological processes in Alzheimer's disease: an updated hypothetical model of dynamic biomarkers. *Lancet Neurol*. 2013;12:207-216.
152. Insel PS, Donohue MC, Berron D, Hansson O, Mattsson-Carlgren N. Time between milestone events in the Alzheimer's disease amyloid cascade. *Neuroimage*. 2021;227:117676.
153. Jagust WJ, Landau SM, Alzheimer's Disease Neuroimaging I. Temporal dynamics of beta-amyloid accumulation in aging and Alzheimer disease. *Neurology*. 2021;96:e1347-e1357.
154. Tosun D, Demir Z, Veitch DP, et al. Contribution of Alzheimer's biomarkers and risk factors to cognitive impairment and decline across the Alzheimer's disease continuum. *Alzheimers Dement*. 2022;18:1370-1382.
155. Damotte V, van der Lee SJ, Chouraki V, et al. Plasma amyloid β levels are driven by genetic variants near APOE, BACE1, APP, PSEN2: A genome-wide association study in over 12,000 non-demented participants. *Alzheimers Dement*. 2021;17:1663-1674.

156. Tan MS, Yang YX, Xu W, et al. Associations of Alzheimer's disease risk variants with gene expression, amyloidosis, tauopathy, and neurodegeneration. *Alzheimers Res Ther*. 2021;13:15.
157. Bellenguez C, Kucukali F, Jansen IE, et al. New insights into the genetic etiology of Alzheimer's disease and related dementias. *Nat Genet*. 2022;54:412-436.
158. Xicota L, Gyorgy B, Grenier-Boley B, et al. Association of APOE-Independent Alzheimer disease polygenic risk score with brain amyloid deposition in asymptomatic older adults. *Neurology*. 2022;99:e462-e475.
159. Wang R, Chopra N, Nho K, et al. Human microRNA (miR-20b-5p) modulates Alzheimer's disease pathways and neuronal function, and a specific polymorphism close to the MIR20B gene influences Alzheimer's biomarkers. *Mol Psychiatry*. 2022;27:1256-1273.
160. Xiong W, Cai J, Li R, Wen C, Tan H, Database AsDNI. Rare variant analysis and molecular dynamics simulation in Alzheimer's disease identifies exonic variants in FLG. *Genes*. 2022;13:838.
161. Femminella GD, Harold D, Scott J, Williams J, Edison P, Alzheimer's Disease Neuroimaging I. The differential influence of immune, endocytotic, and lipid metabolism genes on amyloid deposition and neurodegeneration in subjects at risk of Alzheimer's disease. *J Alzheimers Dis*. 2021;79:127-139.
162. Mullins R, Kapogiannis D. Alzheimer's disease-related genes identified by linking spatial patterns of pathology and gene expression. *Front Neurosci*. 2022;16:908650.
163. Pyun JM, Park YH, Hodges A, et al. Immunity gene IFITM3 variant: Relation to cognition and Alzheimer's disease pathology. *Alzheimers Dement (Amst)*. 2022;14:e12317.
164. Kamagata K, Andica C, Takabayashi K, et al. Association of MRI indices of glymphatic system with amyloid deposition and cognition in mild cognitive impairment and Alzheimer disease. *Neurology*. 2022;99:e2648-e2660.
165. Chandra A, Farrell C, Wilson H, et al. Aquaporin-4 polymorphisms predict amyloid burden and clinical outcome in the Alzheimer's disease spectrum. *Neurobiol Aging*. 2021;97:1-9.
166. Zeng Q, Li K, Luo X, et al. The association of enlarged perivascular space with microglia-related inflammation and Alzheimer's pathology in cognitively normal elderly. *Neurobiol Dis*. 2022;170:105755.
167. Shi X, Zhou N, Sun B, et al. Perivascular space predicts brain hypometabolism of individuals with underlying amyloid pathology. *J Alzheimers Dis*. 2022;90:1329-1337.
168. Wang ML, Yu MM, Wei XE, Li WB, Li YH, Alzheimer's Dis N. Association of enlarged perivascular spaces with A beta and tau deposition in cognitively normal older population. *Neurobiol Aging*. 2021;100:32-38.
169. Wang ML, Zou QQ, Sun Z, et al. Associations of MRI-visible perivascular spaces with longitudinal cognitive decline across the Alzheimer's disease spectrum. *Alzheimers Res Ther*. 2022;14:185.
170. Han F, Chen J, Belkin-Rosen A, et al. Reduced coupling between cerebrospinal fluid flow and global brain activity is linked to Alzheimer disease-related pathology. *PLoS Biol*. 2021;19:e3001233.
171. Xu W, Tan CC, Zou JJ, Cao XP, Tan L, Alzheimers Disease Neuroimaging I. Insomnia moderates the relationship between amyloid-beta and cognitive decline in late-life adults without dementia. *J Alzheimers Dis*. 2021;81:1701-1710.
172. Kim H, Levine A, Cohen D, et al. The role of amyloid, tau, and apoe genotype on the relationship between informant-reported sleep disturbance and Alzheimer's disease risks. *J Alzheimers Dis*. 2022;87:1567-1580.
173. Bubu OM, Umasabor-Bubu OQ, Turner AD, et al. Self-reported obstructive sleep apnea, amyloid and tau burden, and Alzheimer's disease time-dependent progression. *Alzheimers Dement*. 2021;17:226-245.
174. Park YH, Pyun JM, Hodges A, et al. Dysregulated expression levels of APH1B in peripheral blood are associated with brain atrophy and amyloid-beta deposition in Alzheimer's disease. *Alzheimers Res Ther*. 2021;13:10.
175. Li TR, Lyu DY, Liu FQ, Alzheimer's Disease Neuroimaging I. Cerebrospinal fluid sTREM2 in Alzheimer's disease Is associated with both amyloid and tau pathologies but not with cognitive status. *J Alzheimers Dis*. 2022;90:1123-1138.
176. Chen YH, Lin RR, Huang HF, Xue YY, Tao QQ. Microglial activation, tau pathology, and neurodegeneration biomarkers predict longitudinal cognitive decline in Alzheimer's disease continuum. *Front Aging Neurosci*. 2022;14:848180.
177. Leng F, Zhan Z, Sun Y, et al. Cerebrospinal fluid strem2 has paradoxical association with brain structural damage rate in early- and late-stage Alzheimer's disease. *J Alzheimers Dis*. 2022;88:117-126.
178. Pillai JA, Khrestian M, Bena J, Leverenz JB, Bekris LM. Temporal ordering of inflammatory analytes sTNFR2 and sTREM2 in relation to Alzheimer's disease biomarkers and clinical outcomes. *Front Aging Neurosci*. 2021;13:676744.
179. Wang ZB, Ma YH, Sun Y, Tan L, Wang HF, Yu JT. Interleukin-3 is associated with sTREM2 and mediates the correlation between amyloid-β and tau pathology in Alzheimer's disease. *J Neuroinflammation*. 2022;19:316.
180. Shi X, Zhong X, Zhou H, Zhou N, Hu Y, Ning Y. The association between cerebrospinal ferritin and soluble triggering receptor expressed on myeloid cells 2 along Alzheimer's continuum. *Front Neurol*. 2022;13:961842.
181. Pan R, Luo S, Huang Q, et al. The associations of cerebrospinal fluid ferritin with neurodegeneration and neuroinflammation along the Alzheimer's disease continuum. *J Alzheimers Dis*. 2022;88:1115-1125.
182. Ayton S, Janelidze S, Kalinowski P, et al. CSF ferritin in the clinicopathological progression of Alzheimer's disease and associations with APOE and inflammation biomarkers. *J Neurol Neurosurg Psychiatry*. 2023;94:211-219.
183. Contreras JA, Aslanyan V, Albrecht DS, Mack WJ, Pa J. Higher baseline levels of CSF inflammation increase risk of incident mild cognitive impairment and Alzheimer's disease dementia. *Alzheimers Dement*. 2022;14:e12346.
184. Xu W, Tan CC, Cao XP, Tan L, Alzheimer's Disease Neuroimaging I. Neuroinflammation modulates the association of PGRN with cerebral amyloid-beta burden. *Neurobiol Aging*. 2021;103:52-59.
185. Bernier RA, Banks SJ, Panizzon MS, et al. The neuroinflammatory marker sTNFR2 relates to worse cognition and tau in women across the Alzheimer's disease spectrum. *Alzheimers Dement*. 2022;14:e12284.
186. Clark AL, Weigand AJ, Thomas KR, et al. Elevated inflammatory markers and arterial stiffening exacerbate tau but not amyloid pathology in older adults with mild cognitive impairment. *J Alzheimers Dis*. 2021;80:1451-1463.
187. Wu Q, Kong W, Wang S. Peripheral blood biomarkers CXCL12 and TNFRSF13C associate with cerebrospinal fluid biomarkers and infiltrating immune cells in Alzheimer disease. *J Mol Neurosci*. 2021;71:1485-1494.
188. Li Y, Huang X, Fowler C, et al. Identification of leukocyte surface P2×7 as a biomarker associated with Alzheimer's disease. *Int J Mol Sci*. 2022;23:7687.
189. Huang J, Tao Q, Ang TFA, et al. The impact of increasing levels of blood C-reactive protein on the inflammatory loci SPI1 and CD33 in Alzheimer's disease. *Transl Psychiatry*. 2022;12:523.
190. Tao Q, Alvin Ang TF, Akhter-Khan SC, et al. Impact of C-reactive protein on cognition and Alzheimer disease biomarkers in homozygous apolipoprotein E varepsilon4 carriers. *Neurology*. 2021;97:e1243-e1252.

191. Patel D, Zhang X, Farrell JJ, Lunetta KL, Farrer LA. Set-based rare variant expression quantitative trait loci in blood and brain from Alzheimer disease study participants. *Genes (Basel)*. 2021;12:419.
192. Neumann A, Kucukali F, Bos I, et al. Rare variants in IFFO1, DTNB, NLRC3 and SLC22A10 associate with Alzheimer's disease CSF profile of neuronal injury and inflammation. *Mol Psychiatry*. 2022;27:1990-1999.
193. Horgusluoglu E, Neff R, Song WM, et al. Integrative metabolomics-genomics approach reveals key metabolic pathways and regulators of Alzheimer's disease. *Alzheimers Dement*. 2022;18:1260-1278.
194. Novotny BC, Fernandez MV, Wang C, et al. Metabolomic and lipidomic signatures in autosomal dominant and late-onset Alzheimer's disease brains. *Alzheimers Dement*. 2023;19:1785-1799.
195. Nho K, Kueider-Paisley A, Arnold M, et al. Serum metabolites associated with brain amyloid beta deposition, cognition and dementia progression. *Brain Commun*. 2021;3:fcab139.
196. Shanks HR, Onuska KM, Barupal DK, Schmitz TW. Initiative AsDN, consortium AsDM. Serum unsaturated phosphatidylcholines predict longitudinal basal forebrain degeneration in Alzheimer's disease. *Brain Commun*. 2022;4:fcac318.
197. Sakr F, Dyrba M, Brauer A, Teipel S, Alzheimer's Disease Neuroimaging I. Association of lipidomics signatures in blood with clinical progression in preclinical and prodromal Alzheimer's disease. *J Alzheimers Dis*. 2022;85:1115-1127.
198. Baloni P, Arnold M, Buitrago L, et al. Multi-Omic analyses characterize the ceramide/sphingomyelin pathway as a therapeutic target in Alzheimer's disease. *Commun Biol*. 2022;5:13.
199. Ashleigh T, Swerdlow RH, Beal MF. The role of mitochondrial dysfunction in Alzheimer's disease pathogenesis. *Alzheimers Dement*. 2023;19:333-342.
200. Paliwal D, McInerney TW, Pa J, et al. Mitochondrial pathway polygenic risk scores are associated with Alzheimer's disease. *Neurobiol Aging*. 2021;108:213-222.
201. Xu X, Wang H, Bennett DA, Zhang QY, Wang G, Zhang HY. Systems genetic identification of mitochondrion-associated Alzheimer's disease genes and implications for disease risk prediction. *Biomedicines*. 2022;10:20.
202. Stage E, Risacher SL, Lane KA, et al. Association of the top 20 Alzheimer's disease risk genes with [(18)F]flortaucipir PET. *Alzheimers Dement (Amst)*. 2022;14:e12308.
203. Franzmeier N, Ossenkoppele R, Brendel M, et al. The BIN1 rs744373 Alzheimer's disease risk SNP is associated with faster Abeta-associated tau accumulation and cognitive decline. *Alzheimers Dement*. 2022;18:103-115.
204. Pelgrim TAD, Beran M, Twait EL, Geerlings MI, Vonk JMJ. Cross-sectional associations of tau protein biomarkers with semantic and episodic memory in older adults without dementia: a systematic review and meta-analysis. *Ageing Res Rev*. 2021;71:101449.
205. Guo Y, Yang YX, Zhang YR, et al. Genome-wide association study of brain tau deposition as measured by (18)F-flortaucipir positron emission tomography imaging. *Neurobiol Aging*. 2022;120:128-136.
206. Belloy ME, Eger SJ, Le Guen Y, et al. KL*VS heterozygosity reduces brain amyloid in asymptomatic at-risk APOE*4 carriers. *Neurobiol Aging*. 2021;101:123-129.
207. Neitzel J, Franzmeier N, Rubinski A, et al. KL-VS heterozygosity is associated with lower amyloid-dependent tau accumulation and memory impairment in Alzheimer's disease. *Nat Commun*. 2021;12:3825.
208. Picard C, Nilsson N, Labonté A, et al. Apolipoprotein B is a novel marker for early tau pathology in Alzheimer's disease. *Alzheimers Dement*. 2022;18:875-887.
209. Khan AF, Adewale Q, Baumeister TR, et al. Personalized brain models identify neurotransmitter receptor changes in Alzheimer's disease. *Brain*. 2022;145:1785-1804.
210. Qiang Q, Skudder-Hill L, Toyota T, Wei W, Adachi H. CSF GAP-43 as a biomarker of synaptic dysfunction is associated with tau pathology in Alzheimer's disease. *Sci Rep*. 2022;12:17392.
211. Lan G, Cai Y, Li A, et al. Association of presynaptic loss with Alzheimer's disease and cognitive decline. *Ann Neurol*. 2022;92:1001-1015.
212. Ohrfelt A, Benedet AL, Ashton NJ, et al. Association of CSF GAP-43 with the rate of cognitive decline and progression to dementia in amyloid-positive individuals. *Neurology*. 2023;100:e275-e285.
213. Libiger O, Shaw LM, Watson MH, et al. Longitudinal CSF proteomics identifies NPTX2 as a prognostic biomarker of Alzheimer's disease. *Alzheimers Dement*. 2021;17:1976-1987.
214. Hong S, Dobricic V, Ohlei O, et al. TMEM106B and CPOX are genetic determinants of cerebrospinal fluid Alzheimer's disease biomarker levels. *Alzheimers Dement*. 2021;17:1628-1640.
215. Prokopenko D, Morgan SL, Mullin K, et al. Whole-genome sequencing reveals new Alzheimer's disease-associated rare variants in loci related to synaptic function and neuronal development. *Alzheimers Dement*. 2021;17:1509-1527.
216. Braak H, Braak E. Staging of Alzheimer's disease-related neurofibrillary changes. *Neurobiol Aging*. 1995;16:271-278.
217. Bilgel M, Wong DF, Moghekar AR, Ferrucci L, Resnick SM. Initiative AsDN. Causal links among amyloid, tau, and neurodegeneration. *Brain Commun*. 2022;4:fcac193.
218. Cui W, Yan C, Yan Z, et al. BMNet: A new region-based metric learning method for early alzheimer's disease identification With FDG-PET Images. *Front Neurosci*. 2022;16:831533.
219. Wen Q, Risacher SL, Xie L, et al. Tau-related white-matter alterations along spatially selective pathways. *Neuroimage*. 2021;226:117560.
220. Rubinski A, Franzmeier N, Dewenter A, et al. Higher levels of myelin are associated with higher resistance against tau pathology in Alzheimer's disease. *Alzheimers Res Ther*. 2022;14:139.
221. Moscoso A, Silva-Rodríguez J, Aldrey JM, et al. (18)F-florbetapir PET as a marker of myelin integrity across the Alzheimer's disease spectrum. *Eur J Nucl Med Mol Imaging*. 2022;49:1242-1253.
222. Seemiller J, Bischof GN, Hoenig MC, et al. Indication of retrograde tau spreading along Braak stages and functional connectivity pathways. *Eur J Nucl Med Mol Imaging*. 2021;48:2272-2282.
223. Steward A, Biel D, Brendel M, et al. Functional network segregation is associated with attenuated tau spreading in Alzheimer's disease. *Alzheimers Dement*. 2023;19:2034-2046.
224. Brabec JL, Lara MK, Tyler AL, Mahoney JM. System-level analysis of Alzheimer's disease prioritizes candidate genes for neurodegeneration. *Front Genet*. 2021;12:625246.
225. Xu J, Xia X, Li Q, et al. A causal association of ANKRD37 with human hippocampal volume. *Mol Psychiatry*. 2022;27:4432-4445.
226. Strom A, Iaccarino L, Edwards L, et al. Cortical hypometabolism reflects local atrophy and tau pathology in symptomatic Alzheimer's disease. *Brain*. 2022;145:713-728.
227. Ye F, Funk Q, Rockers E, Shulman JM, Masdeu JC, Pascual B. In Alzheimer-prone brain regions, metabolism and risk-gene expression are strongly correlated. *Brain Commun*. 2022;4:fcac216.
228. Kim BH, Nho K, Lee JM, Alzheimer's Disease Neuroimaging I. Genome-wide association study identifies susceptibility loci of brain atrophy to NFIA and ST18 in Alzheimer's disease. *Neurobiol Aging*. 2021;102:200 e1-e11.
229. Lahti J, Tuominen S, Yang Q, et al. Genome-wide meta-analyses reveal novel loci for verbal short-term memory and learning. *Mol Psychiatry*. 2022;27:4419-4431.
230. Gao Y, Felsky D, Reyes-Dumeyer D, et al. Integration of GWAS and brain transcriptomic analyses in a multiethnic sample of 35,245 older adults identifies DCDC2 gene as predictor of episodic memory maintenance. *Alzheimers Dement*. 2022;18:1797-1811.

231. Janssen O, Jansen WJ, Vos SJB, et al. Characteristics of subjective cognitive decline associated with amyloid positivity. *Alzheimers Dement*. 2022;18:1832-1845.
232. Montembeault M, Stijelja S, Brambati SM, Alzheimers Dis N. Self-reported word-finding complaints are associated with cerebrospinal fluid amyloid beta and atrophy in cognitively normal older adults. *Alzheimers Dement (Amst)*. 2022;14.
233. Kuhn E, Perrotin A, Tomadesso C, et al. Subjective cognitive decline: opposite links to neurodegeneration across the Alzheimer's continuum. *Brain Commun*. 2021;3:fcab199.
234. Cacciamani F, Godefroy V, Brambati SM, Migliaccio R, Epelbaum S, Montembeault M. Differential patterns of domain-specific cognitive complaints and awareness across the Alzheimer's disease spectrum. *Front Aging Neurosci*. 2022;14:811739.
235. Gagliardi G, Kuppe M, Lois C, Hanseeuw B, Vannini P, Alzheimer's Disease Neuroimaging I. Pathological correlates of impaired self-awareness of memory function in Alzheimer's disease. *Alzheimers Res Ther*. 2021;13:118.
236. Shen XN, Kuo K, Yang YX, et al. Subtle cognitive impairment as a marker of Alzheimer's pathologies and clinical progression in cognitively normal individuals. *Alzheimers Dement (Amst)*. 2021;13:e12198.
237. Cersonsky TEK, Mechery S, Carper MM, et al. Using the montreal cognitive assessment to identify individuals with subtle cognitive decline. *Neuropsychology*. 2022;36:373-383.
238. Cersonsky TEK, Mechery S, Thompson L, et al. Related amyloid burden and cortical atrophy in individuals with subtle cognitive decline. *J Neuroimaging*. 2022;32:1075-1079.
239. McCollum LE, Das SR, Xie L, et al. Oh brother, where art tau? Amyloid, neurodegeneration, and cognitive decline without elevated tau. *NeuroImage Clin*. 2021;31:102717.
240. Chandler H, Wise R, Linden D, Williams J, Murphy K, Lancaster TM. Alzheimer's genetic risk effects on cerebral blood flow across the lifespan are proximal to gene expression. *Neurobiol Aging*. 2022;120:1-9.
241. Bangen KJ, Thomas KR, Sanchez DL, et al. Entorhinal perfusion predicts future memory decline, neurodegeneration, and white matter hyperintensity progression in older adults. *J Alzheimers Dis*. 2021;81:1711-1725.
242. Ottoy J, Ozzoude M, Zukotynski K, et al. Vascular burden and cognition: mediating roles of neurodegeneration and amyloid PET. *Alzheimers Dement*. 2023;19:1503-1517.
243. Wong FCC, Saffari SE, Yatawara C, Ng KP, Kandiah N. Influence of white matter hyperintensities on baseline and longitudinal amyloid-β in cognitively normal individuals. *J Alzheimers Dis*. 2021;84:91-101.
244. Kamal F, Morrison C, Maranzano J, Zeighami Y, Dadar M. Topographical differences in white matter hyperintensity burden and cognition in aging, MCI, and AD. *Geroscience*. 2023;45:1-16.
245. Tan CH, Chew J, Zhang L, Gulyás B, Chen C. Differential effects of white matter hyperintensities and regional amyloid deposition on regional cortical thickness. *Neurobiol Aging*. 2022;115:12-19.
246. Riphagen JM, Suresh MB, Salat DH. Initiative AsDN. The canonical pattern of Alzheimer's disease atrophy is linked to white matter hyperintensities in normal controls, differently in normal controls compared to in AD. *Neurobiol Aging*. 2022;114:105-112.
247. Collij LE, Salvado G, Wottschel V, et al. Spatial-temporal patterns of beta-amyloid accumulation: a subtype and stage inference model analysis. *Neurology*. 2022;98:e1692-e1703.
248. Vogel JW, Young AL, Oxtoby NP, et al. Four distinct trajectories of tau deposition identified in Alzheimer's disease. *Nat Med*. 2021;27:871-881.
249. Frontzkowski L, Ewers M, Brendel M, et al. Earlier Alzheimer's disease onset is associated with tau pathology in brain hub regions and facilitated tau spreading. *Nat Commun*. 2022;13:4899.
250. Toledo JB, Liu H, Grothe MJ, et al. Disentangling tau and brain atrophy cluster heterogeneity across the Alzheimer's disease continuum. *Alzheimers Dement*. 2022;8:e12305.
251. Young CB, Winer JR, Younes K, et al. Divergent cortical tau positron emission tomography patterns among patients with preclinical Alzheimer disease. *JAMA Neurol*. 2022;79:592-603.
252. Levin F, Ferreira D, Lange C, et al. Data-driven FDG-PET subtypes of Alzheimer's disease-related neurodegeneration. *Alzheimers Res Ther*. 2021;13:49.
253. Groot C, Risacher SL, Chen JQA, et al. Differential trajectories of hypometabolism across cognitively-defined Alzheimer's disease subgroups. *NeuroImage Clin*. 2021;31:102725.
254. Tondo G, Carli G, Santangelo R, et al. Biomarker-based stability in limbic-predominant amnestic mild cognitive impairment. *Eur J Neurol*. 2021;28:1123-1133.
255. Archetti D, Young AL, Oxtoby NP, et al. Inter-cohort validation of sustain model for Alzheimer's disease. *Front Big Data*. 2021;4:661110.
256. Ferreira D, Nordberg A, Westman E. Biological subtypes of Alzheimer disease: a systematic review and meta-analysis. *Neurology*. 2020;94:436-448.
257. Mohanty R, Ferreira D, Frerich S, et al. Neuropathologic features of antemortem atrophy-based subtypes of Alzheimer disease. *Neurology*. 2022;99:e323-e333.
258. Duong MT, Das SR, Lyu X, et al. Dissociation of tau pathology and neuronal hypometabolism within the ATN framework of Alzheimer's disease. *Nat Commun*. 2022;13:1495.
259. Chen P, Yao H, Tijms BM, et al. Four distinct subtypes of Alzheimer's disease based on resting-state connectivity biomarkers. *Biol Psychiatry*. 2023;93:759-769.
260. Cairns NJ, Perrin RJ, Franklin EE, et al. Neuropathologic assessment of participants in two multi-center longitudinal observational studies: the Alzheimer Disease Neuroimaging Initiative (ADNI) and the Dominantly Inherited Alzheimer Network (DIAN). *Neuropathology*. 2015;35:390-400.
261. Constant AB, Basavaraju R, France J, Honig LS, Marder KS, Provenzano FA. Longitudinal patterns of cortical atrophy on MRI in patients with Alzheimer disease with and without Lewy body pathology. *Neurology*. 2022;99:e1843-e1852.
262. Das SR, Lyu XY, Duong MT, et al. Tau-atrophy variability reveals phenotypic heterogeneity in Alzheimer's Disease. *Ann Neurol*. 2021;90:751-762.
263. Woodworth DC, Sheikh-Bahaei N, Scambray KA, et al. Dementia is associated with medial temporal atrophy even after accounting for neuropathologies. *Brain Commun*. 2022; 4:fcac052.
264. Katsumata Y, Shade LM, Hohman TJ, et al. Multiple gene variants linked to Alzheimer's-type clinical dementia via GWAS are also associated with non-Alzheimer's neuropathologic entities. *Neurobiol Dis*. 2022:174:105880.
265. Jack Jr. CR, Knopman DS, Chételat G, Dickson D, et al. Suspected non-Alzheimer disease pathophysiology—concept and controversy. *Nat Rev Neurol*. 2016;12:117-124.
266. Wisse LEM, de Flores R, Xie L, et al. Pathological drivers of neurodegeneration in suspected non-Alzheimer's disease pathophysiology. *Alzheimers Res Ther*. 2021;13:100.
267. Guo T, Landau SM, Jagust WJ, Alzheimer's disease neuroimaging I. Age, vascular disease, and Alzheimer's disease pathologies in amyloid negative elderly adults. *Alzheimers Res Ther*. 2021;13:174.
268. Tijms BM, Gobom J, Reus L, et al. Pathophysiological subtypes of Alzheimer's disease based on cerebrospinal fluid proteomics. *Brain*. 2020;143:3776-3792.
269. Tijms BM, Gobom J, Teunissen C, et al. CSF proteomic Alzheimer's disease-predictive subtypes in cognitively intact amyloid negative individuals. *Proteomes*. 2021;9:36.

270. Liu H, Zhang Y, Zhao H, Du Y, Liu X, Initiative AsDN. The heterogeneity among subgroups of haplogroup J influencing Alzheimer's disease risk. *J Adv Res*. 2021;33:117-126.
271. Stern Y. Cognitive reserve in ageing and Alzheimer's disease. *Lancet Neurol*. 2012;11:1006-1012.
272. Lee DH, Seo SW, Roh JH, et al. Effects of cognitive reserve in Alzheimer's disease and cognitively unimpaired individuals. *Front Aging Neurosci*. 2022;13:784054.
273. Lin L, Sun Y, Wang X, Su L, Wang X, Han Y. Resilience to plasma and cerebrospinal fluid amyloid-beta in cognitively normal individuals: findings from two cohort studies. *Front Aging Neurosci*. 2021;13:610755.
274. Ewers M, Luan Y, Frontzkowski L, et al. Segregation of functional networks is associated with cognitive resilience in Alzheimer's disease. *Brain*. 2021;144:2176-2185.
275. Li T, Wang B, Gao Y, et al. APOE epsilon4 and cognitive reserve effects on the functional network in the Alzheimer's disease spectrum. *Brain Imaging Behav*. 2021;15:758-771.
276. Langella S, Sadiq MU, Mucha PJ, Giovanello KS, Dayan E. Lower functional hippocampal redundancy in mild cognitive impairment. *Transl Psychiatry*. 2021;11:61.
277. Fischer FU, Wolf D, Tüscher O, Fellgiebel A. Structural network efficiency predicts resilience to cognitive decline in elderly at risk for Alzheimer's disease. *Front Aging Neurosci*. 2021;13:637002.
278. Salvado G, Ferreira D, Operto G, et al. The protective gene dose effect of the APOE epsilon2 allele on gray matter volume in cognitively unimpaired individuals. *Alzheimers Dement*. 2022;18:1383-1395.
279. Salvado G, Grothe MJ, Groot C, et al. Differential associations of APOE-epsilon2 and APOE-epsilon4 alleles with PET-measured amyloid-beta and tau deposition in older individuals without dementia. *Eur J Nucl Med Mol Imaging*. 2021;48:2212-2224.
280. Seto M, Mahoney ER, Dumitrescu L, et al. Exploring common genetic contributors to neuroprotection from amyloid pathology. *Brain Commun*. 2022;4:fcac066.
281. Ramanan VK, Lesnick TG, Przybelski SA, et al. Coping with brain amyloid: genetic heterogeneity and cognitive resilience to Alzheimer's pathophysiology. *Acta neuropathologica communications*. 2021;9:48.
282. Hou J, Hess JL, Armstrong N, et al. Polygenic resilience scores capture protective genetic effects for Alzheimer's disease. *Transl Psychiatry*. 2022;12:296.
283. Rahmani A, Najand B, Sonnega A, Akhlaghipour G, Mendez MF, Assari S. Intersectional effects of race and educational attainment on memory function of middle-aged and older adults with Alzheimer's disease. *J Racial Ethn Health Disparities*. 2022:EPUB ahead of print.
284. Martí-Juan G, Sanroma-Guell G, Cacciaglia R, et al. Nonlinear interaction between APOE ε4 allele load and age in the hippocampal surface of cognitively intact individuals. *Hum Brain Mapp*. 2021;42:47-64.
285. Lai YLL, Chen K, Lee TW, et al. The Effect of the APOE-e4 allele on the cholinergic circuitry for subjects with different levels of cognitive impairment. *Frontiers in Neurology*. 2021;12:17.
286. Shi D, Xie S, Li A, et al. APOE-ε4 modulates the association among plasma Aβ(42)/Aβ(40), vascular diseases, neurodegeneration and cognitive decline in non-demented elderly adults. *Transl Psychiatry*. 2022;12:128.
287. Venkatraghavan V, Klein S, Fani L, et al. Analyzing the effect of APOE on Alzheimer's disease progression using an event-based model for stratified populations. *Neuroimage*. 2021;227:117646.
288. Madrid L, Moreno-Grau S, Ahmad S, et al. Multiomics integrative analysis identifies APOE allele-specific blood biomarkers associated to Alzheimer's disease etiopathogenesis. *Aging*. 2021;13:9277-9329.
289. Katabathula S, Davis PB, Xu R. Alzheimer's disease neuroimaging I. Sex-specific heterogeneity of mild cognitive impairment identified based on multi-modal data analysis. *J Alzheimers Dis*. 2023;91:233-243.
290. Sapkota S, Ramirez J, Yhap V, Masellis M, Black SE. Initiative AsDN. Brain atrophy trajectories predict differential functional performance in Alzheimer's disease: Moderations with apolipoprotein E and sex. *Alzheimers Dement*. 2021;13:e12244.
291. Kim JP, Chun MY, Kim SJ, et al. Distinctive temporal trajectories of alzheimer's disease biomarkers according to sex and APOE genotype: importance of striatal amyloid. *Front Aging Neurosci*. 2022;14:829202.
292. Wang YT, Pascoal TA, Therriault J, et al. Interactive rather than independent effect of APOE and sex potentiates tau deposition in women. *Brain Commun*. 2021;3:fcab126.
293. Yan SZ, Zheng CJ, Paranjpe MD, et al. Sex modifies APOE epsilon 4 dose effect on brain tau deposition in cognitively impaired individuals. *Brain*. 2021;144:3201-3211.
294. Chang R, Trushina E, Zhu K, et al. Predictive metabolic networks reveal sex- and APOE genotype-specific metabolic signatures and drivers for precision medicine in Alzheimer's disease. *Alzheimers Dement*. 2023;19:518-531.
295. Tsiknia AA, Reas E, Bangen KJ, et al. Sex and APOE ε4 modify the effect of cardiovascular risk on tau in cognitively normal older adults. *Brain Commun*. 2022;4:fcac035.
296. Eissman JM, Dumitrescu L, Mahoney ER, et al. Sex differences in the genetic architecture of cognitive resilience to Alzheimer's disease. *Brain*. 2022;145:2541-2554.
297. Rhodes E, Insel PS, Butters MA, et al. The impact of amyloid burden and APOE on rates of cognitive impairment in late life depression. *J Alzheimers Dis*. 2021;80:991-1002.
298. Wen J, Fu CHY, Tosun D, et al. Characterizing heterogeneity in neuroimaging, cognition, clinical symptoms, and genetics among patients with late-life depression. *JAMA Psychiatry*. 2022;79:464-474.
299. Touron E, Moulinet I, Kuhn E, et al. Depressive symptoms in cognitively unimpaired older adults are associated with lower structural and functional integrity in a frontolimbic network. *Mol Psychiatry*. 2022;27:5086-5095.
300. Wang ZT, Shen XN, Ma YH, et al. Associations of the rate of change in geriatric depression scale with amyloid and cerebral glucose metabolism in cognitively normal older adults: a longitudinal study. *J Affect Disord*. 2021;280:77-84.
301. Rubin-Norowitz M, Lipton RB, Petersen K, Ezzati A, Alzheimer's Disease Neuroimaging I. Association of depressive symptoms and cognition in older adults without dementia across different biomarker profiles. *J Alzheimers Dis*. 2022;88:1385-1395.
302. Sun L, Li W, Li G, Xiao S. Prefrontal Aβ pathology influencing the pathway from apathy to cognitive decline in non-dementia elderly. *Transl Psychiatry*. 2021;11:534.
303. Sun Y, Xu W, Chen KL, Shen XN, Tan L, Yu JT. Mild behavioral impairment correlates of cognitive impairments in older adults without dementia: mediation by amyloid pathology. *Transl Psychiatry*. 2021;11:577.
304. Gomar JJ, Tan G, Halpern J, Gordon ML, Greenwald B, Koppel J. Increased retention of tau PET ligand [(18)F]-AV1451 in Alzheimer's disease psychosis. *Transl Psychiatry*. 2022;12:82.
305. Ge X, Qiao Y, Choi J, et al. Enhanced association of tau pathology and cognitive impairment in mild cognitive impairment subjects with behavior symptoms. *J Alzheimers Dis*. 2022;87:557-568.
306. Tommasi NS, Gonzalez C, Briggs D, et al. Affective symptoms and regional cerebral tau burden in early-stage Alzheimer's disease. *Int J Geriatr Psychiatry*. 2021;36:1050-1058.
307. Banning LCP, Ramakers I, Rosenberg PB, Lyketsos CG, Leoutsakos JS, Alzheimer's Disease Neuroimaging I. Alzheimer's disease biomarkers as predictors of trajectories of depression and apathy in cognitively normal individuals, mild cognitive impairment, and Alzheimer's disease dementia. *Int J Geriatr Psychiatry*. 2021;36:224-234.

308. Ashford MT, Raman R, Miller G, et al. Screening and enrollment of underrepresented ethnocultural and educational populations in the Alzheimer's Disease Neuroimaging Initiative (ADNI). *Alzheimers Dement*. 2022;18:2603-2613.
309. Gianattasio KZ, Bennett EE, Wei J, et al. Generalizability of findings from a clinical sample to a community-based sample: a comparison of ADNI and ARIC. *Alzheimers Dement*. 2021;17:1265-1276.
310. Yamashita K, Kuwashiro T, Ishikawa K, et al. Right entorhinal cortical thickness is associated with Mini-Mental State Examination scores from multi-country datasets using MRI. *Neuroradiology*. 2022;64:279-288.
311. Duff K, Alzheimer's Disease Neuroimaging I. amnestic MCI in ADNI: Maybe not enough memory impairment? *Neurology*. 2021;97:595-596.
312. Birkenbihl C, Salimi Y, Frohlich H, Japanese Alzheimer's Dis N, Alzheimer's Dis N. Unraveling the heterogeneity in Alzheimer's disease progression across multiple cohorts and the implications for data-driven disease modeling. *Alzheimers Dement*. 2022;18:251-261.
313. Windon C, Iaccarino L, Mundada N, et al. Comparison of plasma and CSF biomarkers across ethnoracial groups in the ADNI. *Alzheimers Dement*. 2022;14:e12315.
314. Schindler SE, Cruchaga C, Joseph A, et al. African Americans have differences in CSF soluble TREM2 and associated genetic variants. *Neurol Genet*. 2021;7:e571.
315. Kim J, Jung SH, Choe YS, et al. Ethnic differences in the frequency of β-amyloid deposition in cognitively normal individuals. *Neurobiol Aging*. 2022;114:27-37.
316. Choi YY, Lee JJ, Choi KY, et al. Multi-racial normative data for lobar and subcortical brain volumes in old age: korean and caucasian norms may be incompatible with each other(†). *Front Aging Neurosci*. 2021;13:675016.
317. Hsieh TJ, Lee WJ, Liao YC, et al. Association between Alzheimer's disease genes and trajectories of cognitive function decline in Han Chinese in Taiwan. *Aging*. 2021;13:17237-17252.
318. Morrison C, Dadar M, Manera AL, Collins DL. Racial differences in white matter hyperintensity burden in older adults. *Neurobiol Aging*. 2023;122:112-119.
319. Guan Y, Ebrahimzadeh SA, Cheng CH, et al. Association of diabetes and hypertension with brain structural integrity and cognition in the boston puerto rican health study cohort. *Neurology*. 2022;98:1534-1544.
320. Mindt MR, Okonkwo O, Weiner MW, et al. Improving generalizability and study design of Alzheimer's disease cohort studies in the United States by including under-represented populations. *Alzheimers Dement*. 2023;19:1549-1557.

SUPPORTING INFORMATION

Additional supporting information can be found online in the Supporting Information section at the end of this article.

How to cite this article: Veitch DP, Weiner MW, Miller M, et al. The Alzheimer's Disease Neuroimaging Initiative in the era of Alzheimer's disease treatment: A review of ADNI studies from 2021 to 2022. *Alzheimer's Dement*. 2024;20:652–694. https://doi.org/10.1002/alz.13449
